# Epidemiological trends and future projections of ischemic stroke in children and adolescents: a global analysis from 1990 to 2021

**DOI:** 10.3389/fneur.2025.1662610

**Published:** 2025-10-22

**Authors:** Yi Liu, Jianhua Hu, Xiaofen Zhu

**Affiliations:** Department of Neurology, KeCheng People’s Hospital, Quzhou, Zhejiang, China

**Keywords:** Global Burden of Disease, ischemic stroke, children and adolescents, inequality, prediction

## Abstract

**Background:**

Ischemic stroke in children and adolescents is a significant public health concern. This study aims to describe the epidemiological characteristics of ischemic stroke in children and adolescents from 1990 to 2021.

**Methods:**

Data on ischemic stroke in children and adolescents from 1990 to 2021 were taken from the 2021 Global Burden of Disease (GBD) study. The annual percentage change (AAPC) was used to assess trends in disease burden by region, age, and gender. To examine regional disparities, the Slope Index of Inequality (SII) and Concentration Index (ConcI) were applied. Additionally, the Bayesian Age-Period-Cohort (BAPC) model was employed to predict the disease burden for the next 15 years.

**Results:**

From 1990 to 2021, ischemic stroke in children and adolescents increased globally in incidence and prevalence, while mortality and disability-adjusted life years (DALYs) decreased. The disease burden is higher in low-income countries, with females having higher incidence rates and males bearing a greater burden in mortality and DALYs. Environmental factors like high temperatures are linked to increased risk. By 2036, incidence and prevalence may rise, particularly in the 15–19 age group, although mortality and DALYs are expected to continue decreasing.

**Conclusion:**

Ischemic stroke in this group shows an “increasing case number but decreasing mortality” trend, highlighting medical progress but ongoing risks. Challenges include gender differences, regional inequalities, and environmental risks. Targeted prevention strategies are needed to reduce the global disease burden.

## 1 Introduction

Based on findings from the Global Burden of Disease (GBD) Study and recent epidemiological evidence, stroke continues to rank among the primary causes of mortality and disability globally (1). In 2021, the global burden included 11.9 million incident stroke cases, resulting in 7.3 million deaths and 160.5 million disability-adjusted life years (DALYs) (2). Ischemic stroke, caused by cerebral artery obstruction, constitutes roughly 85% of all stroke instances (3). While historically considered a neurological disorder of older adults, growing research evidence indicates its occurrence in adolescents and children (4).

The period from childhood to late adolescence involves rapid developmental transitions, including brain maturation, school engagement, and shifts in social determinants of health (5, 6). Ischemic stroke in this population can induce severe neurological complications, often leading to permanent lifelong functional deficits (4, 7). Existing literature has highlighted the clinical severity and substantial burden of pediatric stroke (8). However, comprehensive data on incidence rates—stratified by age subgroup, gender, and regional disparities—remain a critical gap in current knowledge.

To systematically assess the global and regional burden of pediatric stroke, this study aims to analyze age-standardized incidence, prevalence, and years lived with disability (YLDs) of ischemic stroke among 0–19-year-olds across 204 countries from 1990 to 2021. Subgroup analyses by age, gender, and Social Demographic Index (SDI) will be conducted at global, regional, and national levels. Projections of DALYs through 2036 are also included. These insights seek to inform effective prevention strategies, strengthen public health systems, alleviate youth stroke burdens, and promote overall well-being.

## 2 Methods

### 2.1 Data source

This study utilized data from the Global Burden of Disease (GBD) 2021 study to obtain information on ischemic stroke among children and adolescents aged 0–19 years. Data were accessed via the GBD Results Tool.^1^ The dataset included incidence, prevalence, mortality, and disability-adjusted life years (DALYs) for the age groups 0–4, 5–9, 10–14, and 15–19 years, stratified by region and sex. Incidence and prevalence were estimated using the DisMod-MR 2.1 tool (Bayesian meta-regression), while mortality was evaluated using the Cause of Death Ensemble Model (CODEm) framework. Detailed GBD study design and methodology have been extensively described in previous publications (9). Additionally, we used the Socio-Demographic Index (SDI) provided by IHME to reflect the differences in development levels across regions, aiming to analyze the correlation between disease burden and development levels. A higher SDI value, closer to 1, indicates a higher level of development in the region (10).

### 2.2 Cross-country social inequalities analysis

This study uses the Slope Index of Inequality (SII) and Concentration Index (ConcI) to assess the distribution inequality of ischemic stroke burden among children and adolescents across countries. The SII is calculated by performing a linear regression between disease burden and the SDI, while the ConcI uses the Lorenz curve to evaluate relative inequality (2). Smaller absolute values for both indices indicate less variation in disease burden across regions.

### 2.3 Disease prediction

To study changes in disease burden more thoroughly, we applied the Bayesian Age-Period-Cohort (BAPC) model, which incorporates age, period, and cohort effects within a Bayesian framework. The model uses the Integrated Nested Laplace Approximation (INLA) for efficient estimation, avoiding convergence issues of traditional Markov Chain Monte Carlo methods. Its flexibility and robustness make it suitable for projecting long-term disease burden trends (11, 12). The BAPC model has been extensively validated and applied in epidemiological research. With this model, we forecasted the variations in disease burden anticipated over the forthcoming 15-year period, specifically from 2022 to 2036.

### 2.4 Statistical analysis

The age-standardized rate (ASR) was computed per 100,000 individuals as follows (2) (Supplementary Table S1):


$$\mathrm{ASR}=\frac{\sum_{i=1}^{A}a_{i}w_{i}}{\sum_{i=1}^{A}w_{i}}*100,000$$

*a _i_*: the age-specific rate in the *i*th age group; *ω*: the number of people in the corresponding *i*th age group among the standard population; *A*: the number of age groups.

In addition, we constructed the analytical framework using Joinpoint software (version 5.1.0.0; National Cancer Institute, Rockville, Maryland, United States). Up to five joinpoints were set, and the optimal model was selected using the Monte Carlo permutation method. The average annual percentage change (AAPC) with its 95% confidence interval (CI) was estimated to evaluate trends in disease burden (13). All statistical evaluations and visual representations of results were performed using R software (version 4.4.1), with statistical significance established at *p* < 0.05. The indicators analyzed included age-standardized incidence rate (ASIR), age-standardized prevalence rate (ASPR), age-standardized mortality rate (ASMR), and age-standardized DALYs rate (ASDR), with all rates standardized per 100,000 population. We conducted an external validation of the US results in our GBD-based analysis using the publicly available CDC WONDER Underlying Cause of Death database. Given the differing data sources and modeling frameworks between CDC WONDER and GBD, we deliberately restricted the external comparison to children and adolescents stroke ages 0–14 years. Data source & case definition: CDC WONDER Underlying Cause of Death, ICD-10 I60-64 and I69.0–69.4 as underlying cause; population 0–14 years; 1999–2020; GBD Cause of death or injury, United States of America; stroke; 0–14 years; 1999–2020.

## 3 Results

### 3.1 Global trends

From 1990 to 2021, there has been an upward trend in the incidence and prevalence of ischemic stroke among children and adolescents globally. Meanwhile, mortality and DALYs have shown a declining trend (Supplementary Tables S2, S3; Supplementary Figure S1). By 2021, the number of ischemic stroke cases in individuals under 20 years old was approximately 173,630 (95% UI: 97,266–295,831), representing a 5.58% increase from 164,456 cases (95% UI: 93,288–277,393) in 1990. The trend in prevalence is similar to that of incidence, with the number of prevalent cases increasing by approximately 6.02% compared to 1990. However, the numbers of deaths and DALYs have significantly decreased compared to 1990. In 2021, ischemic stroke in children and adolescents resulted in 3,537 deaths (95% UI: 2,735–4,583) and 524,997 DALYs (95% UI: 428,959–636,139), showing a notable decline compared to 1990. This improvement may be attributed to advancements in medical technology.

**Figure 1:**
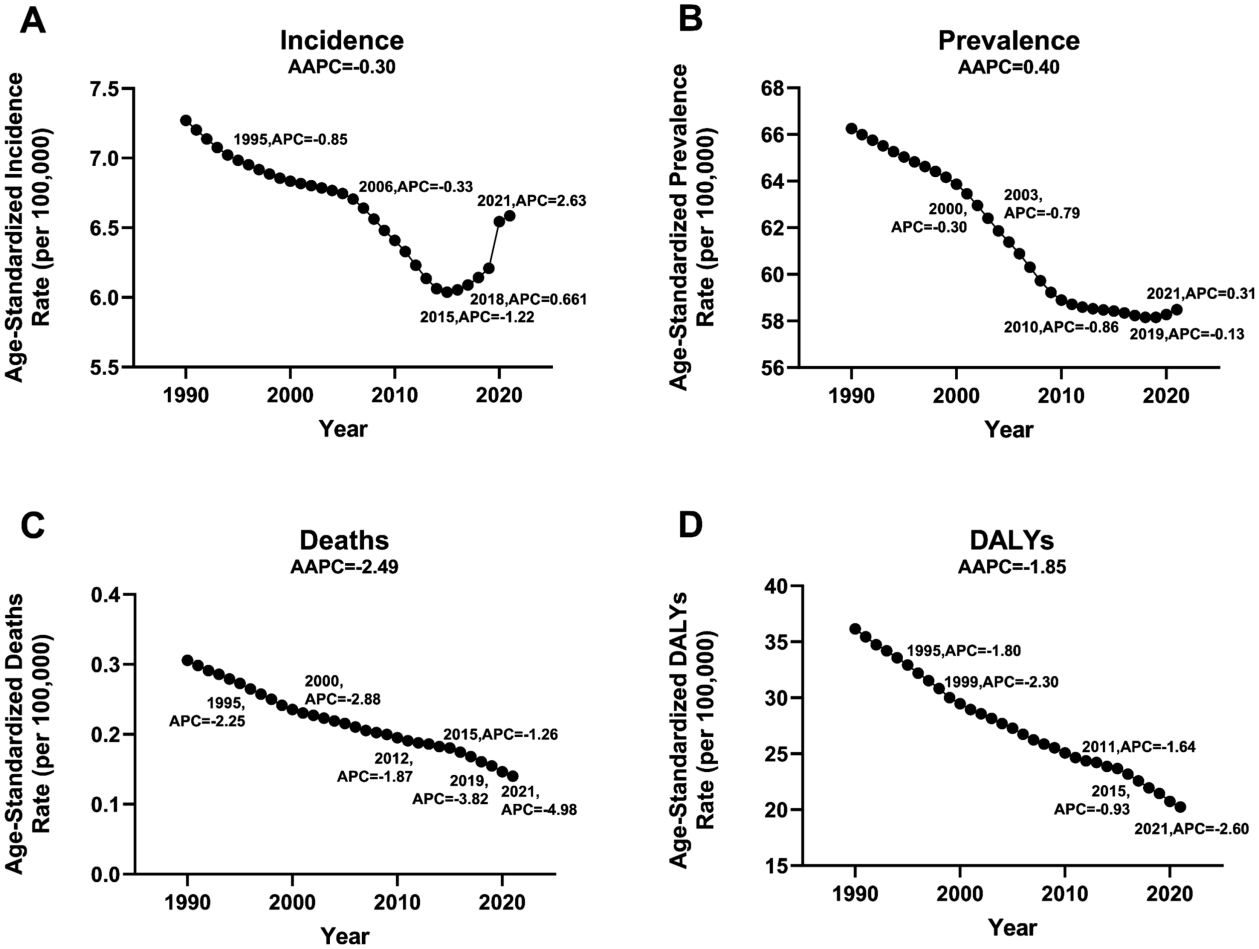
Jointpoint regression analysis of global incidence, prevalence, deaths, and DALYs of ischemic stroke among children and adolescents aged 0 to 19 years from 1990 to 2021. **(A)** Incidence, **(B)** Prevalence, **(C)** Deaths, **(D)** DALYs.

For the population under 20 years old, the age-standardized burden of ischemic stroke has declined compared to 1990. Among these, the decrease in ASMR and ASDR is particularly significant, with the AAPC in ASMR being −2.53 (95% CI: −2.60 to −2.45), and the AAPC in ASDR being −1.84 (95% CI: −1.91 to −1.76). Based on joinpoint analysis, we found that although the overall trend in incidence was declining, there has been a gradual increase since 2015. Particularly after 2018, the increase has been most pronounced, with the APC in ASIR being 2.60 (95% CI: 1.90–3.30; Figure 1; Supplementary Tables S2, S3; Supplementary Figure S1).

### 3.2 Sex and age groups

From the perspective of absolute disease burden, in 2021, the incidence (92,986 cases, 95% UI: 54,148–156,728) and prevalence (854,430 cases, 95%UI: 712,078–1,030,568) among females were higher than those among males (incidence: 74,801 cases, 95%UI: 40,874–128,032; prevalence: 662,753 cases, 95%UI: 557,031–778,257; Supplementary Tables S2, S3; Supplementary Figure S1). However, in terms of the number of deaths and DALYs, males showed a greater burden (number of deaths: females 1,425, 95% UI: 1,132–2,047 vs. males 2,112, 95%UI: 1,380-2,862; number of DALYs: females 260,274, 95%UI: 210,372–314,777 vs. males 264,723, 95%UI: 197,282–334,643; Supplementary Tables S2, S3; Supplementary Figure S1).

In terms of age-standardized burden, in 2021, females had a higher burden than males, except for ASMR. However, from 1990 to 2021, males showed a greater decrease in ASIR and ASPR (AAPC of ASIR: males −0.31, 95% CI: −0.40 to −0.23 vs. females −0.24, 95% CI: −0.41 to −0.08; AAPC of ASPR: males −0.51, 95% CI: −0.54 to −0.49 vs. females −0.30, 95% CI: −0.31 to −0.29; Supplementary Tables S1, S2; Supplementary Figure S1). In terms of ASMR and ASDR, females showed a more significant decline (AAPC of ASMR: males −1.91, 95% CI: −1.99 to −1.82 vs. females −3.20, 95% CI: −3.34 to −3.07; AAPC of ASDR: males −1.58, 95% CI: −1.64 to −1.53 vs. females −2.08, 95% CI: −2.16 to −2.01; Supplementary Tables S2, S3; Supplementary Figure S1).

Additionally, in an in-depth analysis of disease burden across different age groups, adolescents aged 15–19 had the highest incidence of ischemic stroke in 2021 (ASIR 7.39, 95%UI: 3.51–12.83; Supplementary Tables S4, S5). Children under 5 years old experienced the largest decline in incidence from 1990 to 2021 (AAPC −3.23, 95% CI: −3.32 to −3.14). In most age groups, the disease burden for females was higher than for males, including incidence, prevalence, and DALYs (Supplementary Tables S4, S5). Furthermore, we observed that the trend changes across different age groups were consistent with the overall disease trend. Notably, after 2015, the ASIR burden began to rise across all age groups, with females experiencing a greater increase than males from 2015 to 2021(Supplementary Table S6).

### 3.3 Trends by SDI

The majority of incident cases, prevalence, deaths, and DALYs are predominantly concentrated in regions with low and low-middle SDI levels (Supplementary Tables S2, S3). Except for high-income North America, ASIR and ASPR show a negative correlation with SDI levels in most regions. Additionally, ASMR and ASDR initially display a positive correlation with SDI, which subsequently transitions to a negative correlation (Supplementary Figure S2). At the national level in 2021, ASIR and ASPR generally show a negative correlation with SDI levels. However, ASMR and ASDR demonstrate a positive correlation with SDI at lower SDI levels, and as SDI increases, this correlation shifts to negative overall (Supplementary Figure S3).

### 3.4 Region trends

**Figure 2:**
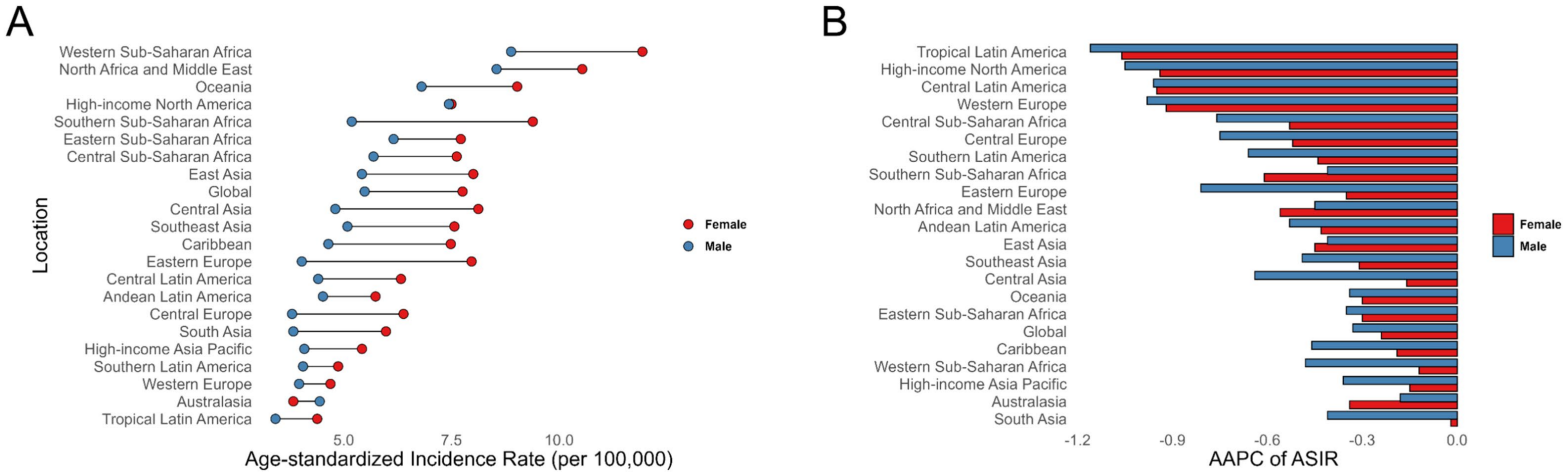
Age-standardized incidence rates **(A)**, along with the average annual percent change from 1990 to 2021 **(B)**, for ischemic stroke among children and adolescents across 21 regions.

In our analysis, we followed the Global Burden of Disease (GBD) classification system, which divides the world into 21 regions (14). In terms of regions, Oceania, Western Sub-Saharan Africa, and High-Income North America have the highest incidence rates, with ASIR of 10.36, 9.57, and 7.76, respectively (Figure 2; Supplementary Table S7). Among these, High-Income North America and Western Sub-Saharan Africa also have the highest ASPR (Supplementary Table S7; Supplementary Figure S4). Regarding ASMR and ASDR, Oceania, Western Sub-Saharan Africa, North Africa and the Middle East, and the Caribbean are the four regions with the heaviest burdens, significantly surpassing other regions. Additionally, in most regions, the disease burden for females exceeds that for males across ASIR, ASPR, ASMR, and ASDR (Supplementary Table S7; Supplementary Figure S4).

From 1990 to 2021, there has been an overall declining trend in disease burden across 21 regions. Tropical Latin America (AAPC of ASIR −1.15, 95% CI: −1.30 to −1.00; AAPC of ASPR −0.91, 95% CI: −0.94 to −0.89), High-Income North America (AAPC of ASIR −0.98, 95% CI: −1.10 to −0.86; AAPC of ASPR −0.75, 95% CI: −0.97 to −0.53), and Central Latin America (AAPC of ASIR −0.96, 95% CI: −1.09 to −0.83; AAPC of ASPR −0.84, 95% CI: −0.86 to −0.81) experienced the largest decreases in ASIR and ASPR for ischemic stroke among children and adolescents(Figure 2; Supplementary Table S7; Supplementary Figure S4). Western Europe (AAPC of ASMR −7.91, 95% CI: −8.62 to −7.19), Australasia (AAPC of ASMR −6.52, 95% CI: −7.63 to −5.40), and High-Income Asia Pacific (AAPC of ASMR −6.45, 95% CI: −6.95 to −5.96) had the largest decreases in ASMR (Supplementary Table S7; Supplementary Figure S4). Andean Latin America (AAPC of ASDR −3.80, 95% CI: −4.16 to −3.43), Central Sub-Saharan Africa (AAPC of ASDR −3.72, 95% CI: −3.84 to −3.59), and North Africa and the Middle East (AAPC of ASDR −3.37, 95% CI: −3.52 to −3.23) saw the largest declines in ASDR (Supplementary Table S7; Supplementary Figure S4).

### 3.5 Country trends

**Figure 3:**
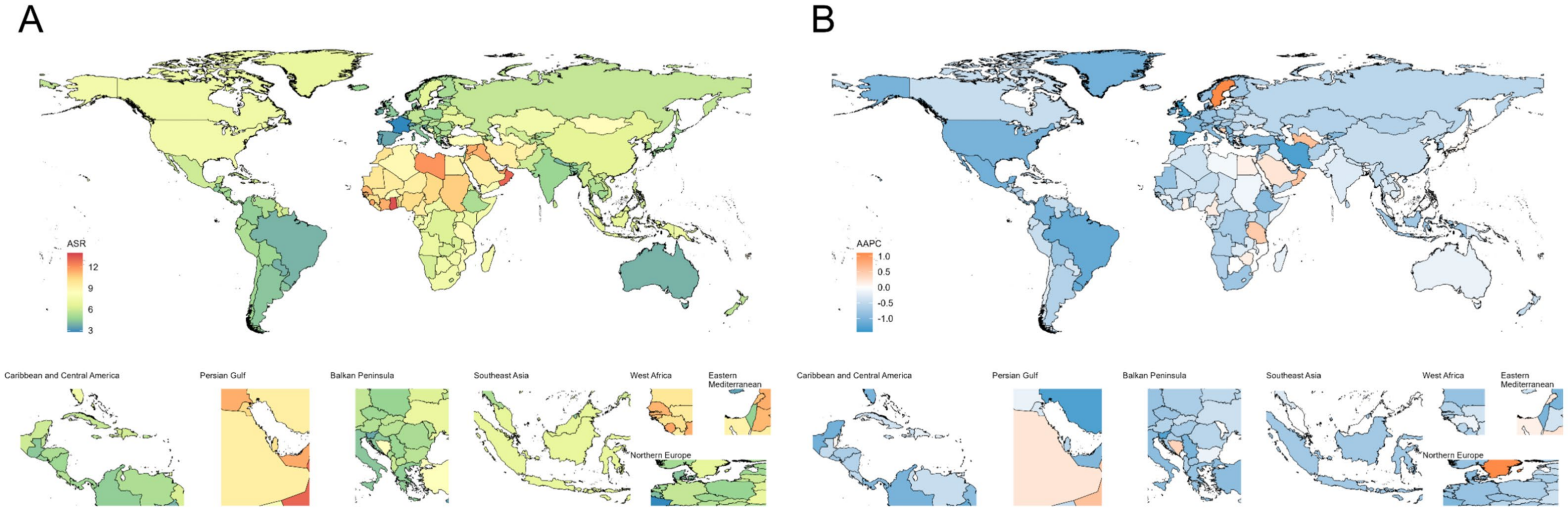
Age-standardized incidence rates **(A)**, along with their average annual percent change **(B)** from 1990 to 2021, for ischemic stroke among children and adolescents across countries.

In 2021, the burden of ischemic stroke among children and adolescents was primarily concentrated in African countries (Figure 3; Supplementary Table S8; Supplementary Figure S5). Nauru had the highest incidence rate (ASIR 13.95, 95% UI: 8.20–22.24) and prevalence rate (ASPR 148.04, 95% UI: 134.19–163.13). Niue (ASMR 1.15, 95% UI: 0.74–1.84; ASDR 121.30, 95% UI: 83.15–182.68), Libya (ASMR 1.12, 95% UI: 0.60–2.14; ASDR 109.45, 95% UI: 64.10–198.48), and Sierra Leone (ASMR 0.87, 95% UI: 0.36–1.60; ASDR 88.84, 95% UI: 42.92–152.61) were the three countries with the heaviest burdens in terms of deaths and DALYs (Supplementary Table S8; Supplementary Figure S5).

From 1990 to 2021, the burden of ischemic stroke among children and adolescents showed a general declining trend in most countries and regions. The United Kingdom was one of the countries with the fastest decline in incidence and prevalence rates, with an AAPC of ASIR at −1.43 (95% CI: −1.63 to −1.23) and an AAPC of ASPR at −1.22 (95% CI: −1.24 to −1.20; Supplementary Table S8; Supplementary Figure S5). Norway experienced the fastest decline in mortality burden, with an AAPC of −10.05 (95% CI: −12.01 to −8.05), while Portugal saw the fastest decline in DALYs burden, with an AAPC of −5.53 (95% CI: −5.90 to −5.16; Supplementary Table S8; Supplementary Figure S5).

### 3.6 Risk factors

**Figure 4:**
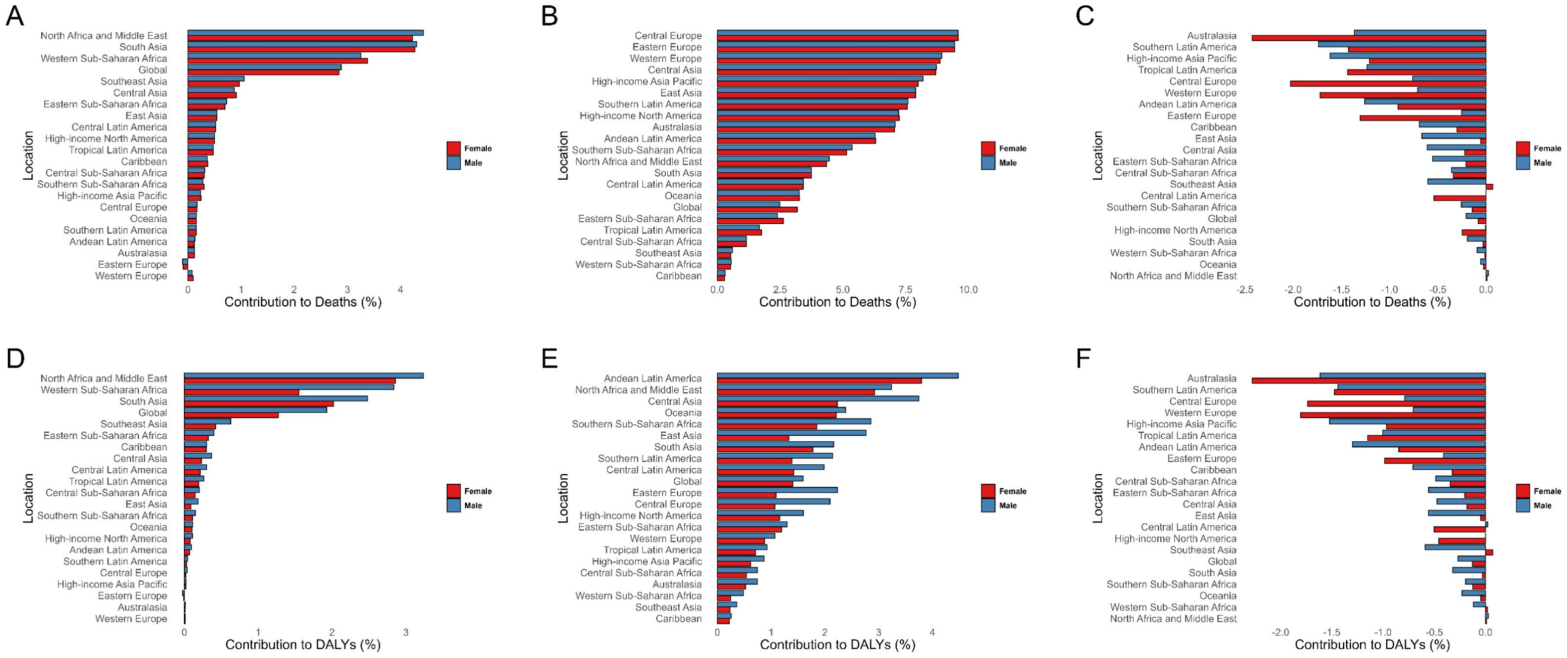
Percentage contribution of risk factors in ischemic stroke among children and adolescents globally and across 21 regions in 2021 by sex. **(A–C)** Percentage contribution of risk factors in ischemic stroke-related deaths. **(C–E)** Percentage contribution of risk factors in ischemic stroke DALYs. High temperature **(A,D)**, low temperature **(B,E)**, and alcohol use **(C,F)**. DALYs, Disability-adjusted life years.

Figure 4 illustrates the associations of high temperatures, low temperatures, and alcohol consumption to ischemic stroke among the population aged 0 to 19. Globally, high and low temperatures show notable associations with ischemic stroke burden in this age group. The burden of ischemic stroke associated with high temperatures exhibits distinct regional characteristics, primarily concentrated in North Africa and the Middle East, South Asia, and Western Sub-Saharan Africa. In contrast, the burden of ischemic stroke deaths associated with low temperatures is predominantly seen in Europe. Andean Latin America, North Africa and the Middle East, and Central Asia are the regions with the highest contribution to ischemic stroke DALYs due to low temperatures. Additionally, regarding the association of low temperatures to ischemic stroke DALYs, we observed that the impact is generally greater on males than on females. From 1990 to 2021, the association of low temperatures to the disease burden has shown a declining trend, while the impact of high temperatures has increased. Further analysis of disease burden across different age groups also revealed this trend (Supplementary Table S9). The contribution of high temperatures to ischemic stroke in those under 20 may further increase.

### 3.7 Inequality analysis

In the analysis of the burden of ischemic stroke among the population under 20 years old across 204 countries and regions, we observed inequalities related to both absolute and relative SDI. Countries and regions with low SDI levels bear a greater disease burden. Except for the incidence rate (SII was −2.747 in 1990 [95% CI: −3.629, −1.865] and −2.761 in 2021 [95% CI: −3.645, −1.878]), which showed a slight increase, the absolute inequality in disease burden among different SDI regions decreased from 1990 to 2021 in terms of prevalence (SII was −22.610 in 1990 [95% CI: −33.028, −12.184] and −19.629 in 2021 [95% CI: −28.981, −10.276]), mortality (SII was −0.360 in 1990 [95% CI: −0.427, −0.293] and −0.199 in 2021 [95% CI: −0.227, −0.712]), and DALYs (SII was −34.085 in 1990 [95% CI: −41.281, −26.890] and −19.454 in 2021 [95% CI: −23.639, −15.268]; Table 1; Supplementary Figure S6).

**Table 1:** The slope index of inequality and the concentration index (ConcI) for ischemic stroke among children and adolescents from 1990 to 2021.

Characteristics	Year	Incidence	Prevalence	Death	DALYs
Slope index of inequality (95% CI)	1990	−2.747 (−3.629, −1.865)	−22.610 (−33.028, −12.184)	−0.360 (−0.427, −0.293)	−34.085 (−41.281, −26.890)
	2021	−2.761 (−3.645, −1.878)	−19.629 (−28.981, −10.276)	−0.199 (−0.227, −0.712)	−19.454 (−23.639, −15.268)
Concentration index (95% CI)	1990	0.185 (0.086, 0.275)	0.145 (0.046, 0.235)	0.337 (0.237, 0.429)	0.286 (0.186, 0.377)
	2021	0.244 (0.149, 0.326)	0.204 (0.111, 0.285)	0.447 (0.356, 0.527)	0.329 (0.238, 0.410)

DALYs, Disability-Adjusted Life Years; CI, confidence interval.

However, the Concentration Index (ConcI) indicates that relative inequality in disease burden between countries and regions of different development levels is further increasing. Whether in terms of incidence (0.185 in 1990 [95% CI: 0.086, 0.275] and 0.244 in 2021 [95% CI: 0.149, 0.326]), prevalence (0.145 in 1990 [95% CI: 0.046, 0.235] and 0.204 in 2021 [95% CI: 0.111, 0.285]), mortality (0.337 in 1990 [95% CI: 0.237, 0.429] and 0.447 in 2021 [95% CI: 0.356, 0.527]), or DALYs (0.286 in 1990 [95% CI: 0.186, 0.377] and 0.329 in 2021 [95% CI: 0.238, 0.410]), relative inequality is on the rise (Table 1; Supplementary Figure S6).

### 3.8 Prediction

Based on the BAPC model, we predict the disease burden of ischemic stroke among children and adolescents. By 2036, the burden measured by ASMR and ASDR is expected to decrease further. It is projected that by 2036, the number of deaths will decline to 1,582 (95% UI: 746 to 2,417), and the ASMR will drop to 0.06 (95% UI: 0.03 to 0.09). Meanwhile, the number of DALYs will decrease to 270,361 (95% UI: 148,958 to 391,763), and the ASDR will fall to 10.4 (95% UI: 5.73 to 15.08; Supplementary Table S10; Supplementary Figure S7).

However, the disease burden in terms of incidence and prevalence is expected to gradually increase. According to our predictions, in 15 years, the number of new ischemic stroke cases will rise to 242,860 (95% UI: 130,866 to 354,855), and the number of prevalent cases will increase to 1,635,804 (95% UI: 1,193,160 to 2,078,449). The ASIR and ASPR are expected to rise to 9.35 (95% UI: 5.04 to 13.66) and 62.95 (95% UI: 45.91 to 79.99), respectively (Supplementary Table S10; Supplementary Figure S7).

External validation results between CDC WONDER and GBD for children and adolescents (0–14 years) stroke death counts and crude mortality rates are in Supplementary Table S11 and Supplementary Figure S8.

## 4 Discussion

This research employed data from the 2021 Global Burden of Disease (GBD) study to systematically evaluate worldwide trends and regional disparities in the prevalence, incidence, mortality, and disability-adjusted life year (DALY) rates of ischemic stroke among children and adolescents under 20 years old. The study also explored their associations with the Social Demographic Index (SDI) at the national level, providing a comprehensive overview of the burden distribution and trends for pediatric ischemic stroke.

Key findings indicated that globally, the incidence and prevalence of ischemic stroke among children and adolescents increased between 1990 and 2021, while mortality rates and DALYs showed significant declines. This phenomenon is likely closely associated with improvements in healthcare, the widespread availability of advanced imaging techniques, and greater awareness of early intervention. These advancements have not only reduced mortality and disability rates but also increased the number of survivors and enabled earlier detection and treatment of cases. This may help explain why incidence and prevalence have risen, whereas mortality and DALYs have continued to decline.

After 2015, the ASIR among individuals under 20 years increased, with a more pronounced rise in females. This trend is likely driven by multiple factors. On one hand, the GBD database methods have been improved since 2019, with hospital and outpatient data processed more accurately and regional differences in healthcare access better adjusted (15). This has increased case detection, especially among females as healthcare availability improved. On the other hand, studies show that adolescent BMI continues to rise, suggesting limited effectiveness of current interventions (16, 17). In addition, over 80% of adolescents have insufficient physical activity, with a higher proportion among females (18). This may partly explain the greater increase in incidence and prevalence in girls. At the same time, males bear a heavier burden in mortality and DALYs, likely due to unhealthy behaviors such as smoking and alcohol consumption that accelerate atherosclerosis. Females may benefit from the neuroprotective effects of estrogen, resulting in lower mortality and DALY burdens. Low- and middle-income countries (LMICs) bore a heavier burden, with more rapid increases in incidence rates. Projections for the next 15 years suggest the global burden of pediatric ischemic stroke will continue to rise, reinforcing its growing role as a major public health challenge—particularly in LMICs.

The study revealed variations in stroke burden across GBD regions, countries, and SDI quartiles. Between 1990 and 2021, age-standardized incidence rates (ASIR), DALYs, and mortality among those under 20 declined overall, aligning with prior GBD findings (19–21). This decline was observed across all SDI regions. In 2021, national-level ASIR and age-standardized prevalence rates (ASPR) were generally inversely correlated with SDI, highlighting socioeconomic disparities. Mortality and DALY burdens were highest in low-SDI regions and lowest in high-SDI regions, likely driven by improved healthcare access and socioeconomic conditions in wealthier areas. Advances in medical technology, genetic screening, and early intervention in high-income countries have also contributed to better stroke outcomes (22, 23). In contrast, medium-high SDI regions faced persistent burdens due to limited healthcare access, suboptimal risk factor management, and inadequate secondary prevention (24, 25).

Regionally, the ASIR, ASMR, ASDR and ASPR varied substantially. Tropical Latin America saw the steepest declines, while Australasia showed minimal change. Oceania, western sub-Saharan Africa, and high-income North America had the highest incidences (10.36, 9.57, and 7.7 ASIR, respectively), with similarly elevated ASPR in the latter two regions. Oceania, western sub-Saharan Africa, North Africa/Middle East, and the Caribbean exhibited the highest ASMR and DALY burdens, attributed to genetic predispositions, risk factor prevalence, and healthcare limitations (26–29). Additionally, we observed that in most regions, females have higher ASIR, whereas in Australasia, males exhibit higher ASIR. This may be related to a lower case detection rate of ischemic stroke among females compared to males in this region (9). Although measures have been implemented to reduce disease burden, the detection rate in females remains relatively low (30). At the country level, most nations showed declining burdens from 1990 to 2021, though African countries like Nauru (highest incidence/prevalence), Niue, Libya, and Sierra Leone (highest mortality/DALYs) remained hotspots. The United Kingdom, Norway, and Portugal demonstrated some of the steepest declines in incidence, mortality, and DALYs, respectively (31, 32).

Risk factor analysis identified extreme temperatures and alcohol consumption as key contributors to global pediatric ischemic stroke burdens. High and low temperatures were particularly impactful, aligning with climate change research (33, 34). However, in population studies, the associations between temperature and stroke are primarily driven by adult data, and specific evidence for children is relatively scarce. Further analytical research is needed. Public education on alcohol-related stroke risks and integration of stroke risk assessments into primary care—focused on modifiable risk factors—emerged as critical intervention strategies (35).

Cross-country inequality analyses showed persistent disparities: absolute inequality is gradually decreasing, while relative inequality continues to intensify, with low Social Development Index (SDI) regions bearing a heavier burden (10, 19, 36). This outcome indicates that although there is an overall reduction in burden, low SDI regions have made some absolute improvements, yet their rate of improvement still lags behind that of high SDI regions. From a policy perspective, the narrowing of absolute gaps affirms the effectiveness of current health policies, whereas the widening of relative gaps suggests a need to more precisely allocate resources toward low SDI areas. Projections using the BAPC model suggested that while ASMR and ASDR would continue to decline by 2036, absolute incidence and prevalence would rise due to population growth and evolving risk factors (37, 38). Thus, strategies must balance reducing relative risks with addressing absolute burdens through primary prevention (e.g., managing adolescent obesity and hypertension) and improving access to stroke screening/rehabilitation (39, 40).

### 4.1 Limitations

This study should be interpreted in light of several limitations. First, all estimates are based on the standardized case definitions used in the Global Burden of Disease (GBD) study, which may differ from clinical diagnostic criteria and introduce some degree of misclassification. Second, pediatric ischemic stroke is a relatively rare condition, and in certain low-resource settings the availability and quality of underlying data are limited, leading to greater statistical uncertainty in regional estimates. Third, the attribution of risk factors in GBD relies on model-based approaches and global exposure-outcome pairs, which may not fully capture age-specific or pediatric-specific mechanisms. Together, these limitations suggest that the results should be viewed as best available estimates rather than precise counts, and underscore the need for more high-quality, pediatric-focused epidemiological data.

## 5 Conclusion

From 1990 to 2021, ischemic stroke in children and adolescents has shown an increasing trend in incidence and prevalence, while mortality rates and disability-adjusted life years (DALYs) have declined. This indicates progress in reducing deaths and long-term disabilities, yet highlights an increasing risk of stroke in younger populations. Gender and age analysis reveals that females have higher incidence and prevalence rates, whereas males experience a greater burden in terms of mortality and DALYs. Countries with lower Socio-Demographic Index (SDI) levels face a higher disease burden, underscoring the urgent need to improve healthcare access and socioeconomic conditions. Projections for the next 15 years suggest continued declines in mortality and DALYs, but a potential rise in incidence and prevalence, emphasizing the critical importance of enhancing stroke prevention and risk factor management.

## Data Availability

The original contributions presented in the study are included in the article/Supplementary material, further inquiries can be directed to the corresponding author.

## Footnotes

^1^ https://vizhub.healthdata.org/gbd-results/
