# Supplementary material for: Epidemiological trends and future projections of ischemic stroke in children and adolescents: a global analysis from 1990 to 2021
Source: Front Neurol. 2025 Oct 22;16:1662610. doi: 10.3389/fneur.2025.1662610 (PMC12586142; doi:10.3389/fneur.2025.1662610)
Supplement: Supplementary file 1 [file Table_1.docx]

**Table S1**

| **Age Group** | **Percent (%)** |
| --- | --- |
| ＜5 | 8.860 |
| 5-9 | 8.690 |
| 10-14 | 8.600 |
| 15-19 | 8.470 |
| 20-24 | 8.220 |
| 25-29 | 7.930 |
| 30-34 | 7.610 |
| 35-39 | 7.150 |
| 40-44 | 6.590 |
| 45-49 | 6.040 |
| 50-54 | 5.370 |
| 55-59 | 4.550 |
| 60-64 | 3.720 |
| 65-69 | 2.960 |
| 70-74 | 2.210 |
| 75-79 | 1.520 |
| 80-84 | 0.910 |
| 85-89 | 0.440 |
| 90-94 | 0.150 |
| 95+ | 0.045 |

**Table S2 Global Incidence and prevalence of Ischemic stroke and their AAPCs from 1990 to 2021 by sex and SDI.**

| **Location** | **Sex** | **Incidence (95%UI)** | | | | |  | **Prevalence (95%UI)** | | | | |
| --- | --- | --- | --- | --- | --- | --- | --- | --- | --- | --- | --- | --- |
|  |  | **Number** | | **ASR per 100,000** | | |  | **Number** | | **ASR per 100,000** | | |
|  |  | **1990** | **2021** | **1990** | **2021** | **AAPC (95%CI)** |  | **1990** | **2021** | **1990** | **2021** | **AAPC (95%CI)** |
| Global | Both | 164456 (93288,277393) | 173630 (97266,295831) | 7.27 (3.37,13.09) | 6.6 (3.06,11.97) | -0.29 (-0.44 - -0.14) |  | 1498216 (1245810,1808353) | 1588348 (1340800,1859241) | 68.32 (55.32,82.94) | 60.44 (50.13,71.47) | -0.39 (-0.41 - -0.38) |
|  | Female | 92986 (54148,156728) | 98829 (56414,169187) | 8.43 (3.91,15.08) | 7.75 (3.61,14) | -0.24 (-0.41 - -0.08) |  | 854430 (712078,1030568) | 925595 (782606,1081102) | 79.65 (64.6,96.69) | 72.56 (60.53,85.66) | -0.30 (-0.31 - -0.29) |
|  | Male | 71470 (39515,121349) | 74801 (40874,128032) | 6.17 (2.75,11.29) | 5.52 (2.46,10.15) | -0.31 (-0.40 - -0.23) |  | 643785 (531849,780812) | 662753 (557031,778257) | 57.48 (46.43,69.84) | 49.02 (40.44,58.2) | -0.51 (-0.54 - -0.49) |
| High SDI | Both | 18463 (10406,30950) | 14040 (7372,23881) | 7.38 (3.43,13.25) | 6.07 (2.66,11.15) | -0.61 (-0.65 - -0.57) |  | 202381 (166117,245828) | 170952 (140761,202787) | 79.19 (63.86,96.85) | 71.32 (58.23,85.38) | -0.36 (-0.52 - -0.20) |
|  | Female | 9467 (5306,16068) | 7249 (3838,12327) | 7.77 (3.55,14.07) | 6.45 (2.84,11.99) | -0.58 (-0.60 - -0.56) |  | 105181 (86078,127881) | 90111 (74068,106838) | 84.53 (67.73,103.13) | 77.49 (63.15,93.17) | -0.31 (-0.46 - -0.17) |
|  | Male | 8995 (5065,15133) | 6791 (3599,11569) | 7.01 (3.22,12.61) | 5.7 (2.48,10.58) | -0.64 (-0.73 - -0.56) |  | 97200 (80088,118378) | 80841 (67000,95632) | 74.11 (59.89,90.39) | 65.5 (53.54,78.36) | -0.39 (-0.48 - -0.29) |
| High-middle SDI | Both | 28265 (16226,47488) | 19226 (10361,33439) | 7.62 (3.52,13.68) | 6.35 (2.8,11.72) | -0.58 (-0.70 - -0.45) |  | 274838 (228343,333878) | 180043 (150958,212550) | 73.1 (59.04,89.15) | 58.73 (48.27,70.21) | -0.70 (-0.73 - -0.66) |
|  | Female | 16296 (9479,27687) | 10988 (6061,19253) | 9.05 (4.27,16.34) | 7.62 (3.35,14.18) | -0.54 (-0.68 - -0.40) |  | 158324 (131179,191603) | 103275 (86698,122103) | 86.29 (69.66,105.51) | 70.78 (58.19,84.7) | -0.64 (-0.66 - -0.62) |
|  | Male | 11969 (6717,20180) | 8238 (4282,14372) | 6.28 (2.79,11.5) | 5.19 (2.21,9.79) | -0.61 (-0.72 - -0.51) |  | 116514 (96502,140755) | 76768 (63980,90546) | 60.53 (48.85,73.59) | 47.79 (39.18,57.17) | -0.76 (-0.79 - -0.72) |
| Low SDI | Both | 22603 (13253,37446) | 43550 (25566,72254) | 8.03 (3.84,14.25) | 7.45 (3.63,13.15) | -0.21 (-0.34 - -0.07) |  | 179810 (153018,208453) | 374720 (326706,426023) | 72.21 (60.7,84.34) | 67.37 (57.8,77.4) | -0.22 (-0.23 - -0.22) |
|  | Female | 12652 (7548,20832) | 24879 (14760,40895) | 8.96 (4.32,15.72) | 8.56 (4.21,15.05) | -0.11 (-0.27 - 0.06) |  | 105800 (90744,122156) | 227385 (198427,257314) | 85.58 (72.45,99.29) | 82.43 (71.24,94.09) | -0.12 (-0.13 - -0.12) |
|  | Male | 9951 (5648,16873) | 18670 (10710,31583) | 7.12 (3.27,12.96) | 6.38 (2.99,11.43) | -0.32 (-0.39 - -0.26) |  | 74010 (61990,86317) | 147334 (127285,168512) | 59.26 (49,69.97) | 52.7 (44.62,61.11) | -0.37 (-0.39 - -0.35) |
| Low-middle SDI | Both | 37910 (20744,64320) | 48450 (27340,82425) | 6.45 (2.91,11.86) | 6.34 (2.96,11.55) | -0.02 (-0.17 - 0.13) |  | 312341 (259106,374504) | 422133 (359173,493001) | 56.97 (46.25,68.67) | 55.15 (46,64.97) | -0.11 (-0.12 - -0.10) |
|  | Female | 21505 (12152,36663) | 27907 (16038,47960) | 7.49 (3.44,13.5) | 7.51 (3.52,13.55) | 0.04 (-0.11 - 0.19) |  | 179829 (149811,215295) | 249423 (212734,290333) | 66.93 (54.36,80.64) | 66.86 (56.02,78.54) | -0.01 (-0.02 - 0.00) |
|  | Male | 16405 (8645,28159) | 20544 (11223,34757) | 5.46 (2.34,10.23) | 5.24 (2.36,9.68) | -0.10 (-0.26 - 0.05) |  | 132511 (109074,159942) | 172710 (144779,202064) | 47.41 (38.22,57.43) | 44.03 (36.41,52.15) | -0.24 (-0.26 - -0.22) |
| Middle SDI | Both | 57077 (32578,96302) | 48232 (26510,83193) | 7.46 (3.47,13.36) | 6.44 (2.87,11.78) | -0.45 (-0.61 - -0.29) |  | 527560 (435514,647193) | 439258 (365677,526060) | 69.48 (55.52,85.83) | 57.88 (46.9,69.91) | -0.59 (-0.62 - -0.56) |
|  | Female | 32986 (19216,55584) | 27730 (15571,48348) | 8.85 (4.14,15.84) | 7.71 (3.44,14.1) | -0.42 (-0.59 - -0.25) |  | 304549 (249980,373748) | 254685 (211985,304932) | 82.01 (65.43,101.55) | 69.82 (56.73,84.47) | -0.53 (-0.55 - -0.50) |
|  | Male | 24092 (13125,40954) | 20502 (10788,35419) | 6.13 (2.74,11.25) | 5.26 (2.23,9.86) | -0.49 (-0.64 - -0.34) |  | 223012 (183217,273462) | 184572 (153066,221240) | 57.48 (45.95,70.85) | 46.83 (37.91,56.63) | -0.66 (-0.69 - -0.62) |

**SDI Socio-Demographic Index, AAPC Average Annual Percent Change, ASR Age-Standardized Rate, UI uncertainty interval, CI confidence interval.**

**Table S3 Global Death and DALYs of Ischemic stroke and their AAPCs from 1990 to 2021 by sex and SDI.**

| **Location** | **Sex** | **Death (95%UI)** | | | | |  | **DALYs (95%UI)** | | | | |
| --- | --- | --- | --- | --- | --- | --- | --- | --- | --- | --- | --- | --- |
|  |  | **Number** | | **ASR per 100,000** | | |  | **Number** | | **ASR per 100,000** | | |
|  |  | **1990** | **2021** | **1990** | **2021** | **AAPC (95%CI)** |  | **1990** | **2021** | **1990** | **2021** | **AAPC (95%CI)** |
| Global | Both | 7021 (5350,10203) | 3537 (2735,4583) | 0.3 (0.22,0.44) | 0.14 (0.1,0.18) | -2.53 (-2.60 - -2.45) |  | 826344 (663378,1118301) | 524997 (428959,636139) | 35.85 (27.18,50.51) | 20.12 (15.24,26.05) | -1.84 (-1.91 - -1.76) |
|  | Female | 3545 (2515,5457) | 1425 (1132,2047) | 0.31 (0.22,0.49) | 0.11 (0.09,0.16) | -3.20 (-3.34 - -3.07) |  | 440881 (337246,624198) | 260274 (210372,314777) | 39.43 (28.9,58.02) | 20.55 (16,27.21) | -2.08 (-2.16 - -2.01) |
|  | Male | 3476 (2556,4846) | 2112 (1380,2862) | 0.29 (0.21,0.41) | 0.16 (0.1,0.22) | -1.91 (-1.99 - -1.82) |  | 385463 (303810,503247) | 264723 (197282,334643) | 32.42 (24.06,44.34) | 19.71 (13.64,26.53) | -1.58 (-1.64 - -1.53) |
| High SDI | Both | 386 (355,440) | 69 (61,77) | 0.16 (0.14,0.18) | 0.03 (0.03,0.03) | -4.90 (-5.36 - -4.43) |  | 61648 (52216,72706) | 29920 (22570,37905) | 24.55 (20.01,30.33) | 12.62 (9.37,16.29) | -2.07 (-2.27 - -1.87) |
|  | Female | 173 (156,202) | 31 (28,35) | 0.14 (0.13,0.17) | 0.03 (0.02,0.03) | -4.91 (-5.46 - -4.37) |  | 31032 (25839,37297) | 16450 (12159,21037) | 25.33 (20.21,31.94) | 14.27 (10.56,18.58) | -1.82 (-2.04 - -1.60) |
|  | Male | 213 (193,242) | 38 (33,43) | 0.17 (0.15,0.2) | 0.03 (0.03,0.04) | -4.91 (-5.36 - -4.45) |  | 30616 (26491,35824) | 13469 (10323,17059) | 23.81 (19.58,29.1) | 11.07 (8.31,14.17) | -2.36 (-2.58 - -2.15) |
| High-middle SDI | Both | 845 (709,1090) | 182 (156,206) | 0.23 (0.18,0.3) | 0.06 (0.05,0.07) | -4.16 (-4.66 - -3.66) |  | 118267 (99756,145543) | 45571 (35464,56793) | 31.73 (24.92,41.9) | 15.06 (11.51,19.03) | -2.37 (-2.55 - -2.19) |
|  | Female | 424 (336,622) | 78 (65,90) | 0.24 (0.18,0.36) | 0.06 (0.04,0.07) | -4.60 (-5.14 - -4.05) |  | 65121 (52927,86118) | 25276 (19047,32117) | 36.02 (27.17,51.38) | 17.5 (12.9,22.52) | -2.28 (-2.37 - -2.20) |
|  | Male | 421 (344,503) | 104 (87,122) | 0.22 (0.17,0.28) | 0.07 (0.05,0.08) | -3.78 (-4.11 - -3.46) |  | 53146 (45227,63389) | 20295 (16298,24988) | 27.69 (21.75,34.95) | 12.85 (9.92,16.21) | -2.47 (-2.66 - -2.27) |
| Low SDI | Both | 1265 (794,2134) | 1402 (874,2108) | 0.39 (0.24,0.64) | 0.23 (0.14,0.35) | -1.66 (-1.73 - -1.58) |  | 132975 (94047,208988) | 168114 (121426,230012) | 42.42 (28.86,65.58) | 28.16 (19.37,39.69) | -1.31 (-1.37 - -1.25) |
|  | Female | 522 (321,971) | 469 (305,772) | 0.33 (0.2,0.62) | 0.16 (0.1,0.27) | -2.32 (-2.42 - -2.22) |  | 59604 (41351,98613) | 70520 (54265,97203) | 40.14 (27.27,66.03) | 24.69 (18.1,36) | -1.56 (-1.63 - -1.50) |
|  | Male | 743 (437,1290) | 933 (471,1489) | 0.43 (0.25,0.75) | 0.29 (0.15,0.47) | -1.24 (-1.33 - -1.15) |  | 73371 (47056,120596) | 97593 (58585,144480) | 44.58 (27.91,72.82) | 31.42 (18.24,47.85) | -1.12 (-1.19 - -1.04) |
| Low-middle SDI | Both | 2531 (1599,4153) | 1226 (967,1602) | 0.4 (0.25,0.66) | 0.16 (0.12,0.22) | -2.87 (-3.04 - -2.71) |  | 261245 (180897,402252) | 159935 (131450,193402) | 41.8 (27.96,64.66) | 21.06 (16.23,27.09) | -2.20 (-2.35 - -2.05) |
|  | Female | 1414 (810,2465) | 557 (413,805) | 0.46 (0.26,0.83) | 0.15 (0.11,0.22) | -3.51 (-3.76 - -3.26) |  | 147948 (92836,241071) | 82323 (66330,101995) | 48.96 (30.77,82.03) | 22.23 (17.01,29.75) | -2.51 (-2.69 - -2.33) |
|  | Male | 1117 (705,1626) | 669 (447,871) | 0.34 (0.21,0.52) | 0.17 (0.11,0.24) | -2.12 (-2.31 - -1.94) |  | 113297 (77947,157443) | 77612 (57622,94828) | 34.94 (23.15,50.88) | 19.92 (13.94,26.38) | -1.78 (-1.90 - -1.66) |
| Middle SDI | Both | 1988 (1656,2715) | 655 (569,757) | 0.26 (0.21,0.36) | 0.09 (0.07,0.11) | -3.37 (-3.47 - -3.27) |  | 251453 (211781,319201) | 120923 (98452,146973) | 32.65 (26.02,44.27) | 16.18 (12.74,20.06) | -2.27 (-2.37 - -2.17) |
|  | Female | 1008 (795,1520) | 288 (242,344) | 0.27 (0.21,0.41) | 0.08 (0.07,0.1) | -3.79 (-3.91 - -3.67) |  | 136742 (111689,184916) | 65407 (50560,81674) | 36.55 (28.06,52.42) | 18.15 (13.83,22.87) | -2.26 (-2.32 - -2.21) |
|  | Male | 979 (773,1220) | 367 (301,442) | 0.25 (0.19,0.32) | 0.1 (0.07,0.12) | -3.02 (-3.12 - -2.92) |  | 114711 (95479,138443) | 55517 (45358,66775) | 28.94 (22.48,37.09) | 14.35 (11.24,17.88) | -2.27 (-2.38 - -2.15) |

**SDI Socio-Demographic Index, AAPC Average Annual Percent Change, DALYs Disability-Adjusted Life Years, ASR Age-Standardized Rate, UI uncertainty interval, CI confidence interval.**

**Table S4 Global Incidence and prevalence of Ischemic stroke and their AAPCs from 1990 to 2021 by sex and age groups.**

| **Age Group** | **Sex** | **Incidence (95% UI)** | | | | | | **Prevalence (95% UI)** | | | | | |
| --- | --- | --- | --- | --- | --- | --- | --- | --- | --- | --- | --- | --- | --- |
|  |  | **Number** | |  | **ASR** | | | **Number** | |  | **ASR** | | |
|  |  | **1990** | **2021** |  | **1990** | **2021** | **AAPC (95% CI)** | **1990** | **2021** |  | **1990** | **2021** | **AAPC (95% CI)** |
| <5 | Both | 49805 (29550,81303) | 44274 (25771,74782) |  | 8.03 (4.77,13.11) | 6.73 (3.92,11.36) | -0.54 (-0.67 - -0.41) | 115454 (99180,134565) | 106700 (93601,121536) |  | 18.62 (16,21.71) | 16.21 (14.22,18.47) | -0.44 (-0.47 - -0.41) |
|  | Female | 28417 (16876,45657) | 27044 (16152,44417) |  | 9.46 (5.62,15.2) | 8.5 (5.08,13.96) | -0.32 (-0.44 - -0.20) | 66334 (57183,76947) | 65679 (57960,74666) |  | 22.08 (19.04,25.61) | 20.64 (18.22,23.47) | -0.21 (-0.24 - -0.19) |
|  | Male | 21388 (12410,35914) | 17229 (9352,30919) |  | 6.69 (3.88,11.24) | 5.07 (2.75,9.09) | -0.90 (-1.04 - -0.77) | 49119 (41851,57586) | 41020 (35612,47090) |  | 15.37 (13.1,18.02) | 12.07 (10.47,13.85) | -0.76 (-0.81 - -0.71) |
| 5-9 | Both | 39230 (14240,76510) | 41190 (14421,80367) |  | 6.72 (2.44,13.11) | 6 (2.1,11.7) | -0.33 (-0.57 - -0.09) | 303987 (240887,377438) | 312165 (255057,378661) |  | 52.09 (41.28,64.68) | 45.44 (37.12,55.11) | -0.44 (-0.48 - -0.40) |
|  | Female | 21988 (7810,43073) | 23573 (8295,46550) |  | 7.75 (2.75,15.18) | 7.09 (2.49,14) | -0.24 (-0.51 - 0.03) | 173134 (137400,214255) | 186047 (152829,225114) |  | 61 (48.41,75.49) | 55.94 (45.95,67.69) | -0.28 (-0.30 - -0.26) |
|  | Male | 17242 (5734,33068) | 17617 (5927,33600) |  | 5.75 (1.91,11.03) | 4.97 (1.67,9.48) | -0.45 (-0.66 - -0.24) | 130854 (103412,163257) | 126118 (102544,153084) |  | 43.66 (34.51,54.47) | 35.58 (28.93,43.19) | -0.65 (-0.69 - -0.61) |
| 10-14 | Both | 35921 (14641,68208) | 42042 (18009,80118) |  | 6.71 (2.73,12.73) | 6.31 (2.7,12.02) | -0.17 (-0.34 - -0.01) | 452243 (360618,554677) | 497205 (404916,594075) |  | 84.42 (67.32,103.55) | 74.58 (60.74,89.12) | -0.40 (-0.44 - -0.36) |
|  | Female | 20181 (8275,38191) | 23257 (9729,44318) |  | 7.72 (3.17,14.61) | 7.2 (3.01,13.72) | -0.20 (-0.38 - -0.01) | 256981 (205246,314757) | 289359 (237593,343805) |  | 98.3 (78.51,120.4) | 89.61 (73.57,106.47) | -0.30 (-0.32 - -0.28) |
|  | Male | 15740 (6375,30958) | 18785 (8227,36659) |  | 5.74 (2.32,11.29) | 5.47 (2.39,10.67) | -0.13 (-0.18 - -0.08) | 195263 (155345,239944) | 207846 (167595,251036) |  | 71.2 (56.64,87.49) | 60.47 (48.76,73.04) | -0.52 (-0.54 - -0.50) |
| 15-19 | Both | 39500 (18199,69544) | 46124 (21920,80064) |  | 7.6 (3.5,13.39) | 7.39 (3.51,12.83) | -0.09 (-0.16 - -0.01) | 626531 (512573,752214) | 672278 (563127,784954) |  | 120.62 (98.68,144.82) | 107.74 (90.25,125.8) | -0.36 (-0.38 - -0.34) |
|  | Female | 22400 (10419,39190) | 24954 (11632,43491) |  | 8.77 (4.08,15.34) | 8.22 (3.83,14.32) | -0.20 (-0.28 - -0.12) | 357982 (293259,431116) | 384510 (323362,449482) |  | 140.09 (114.76,168.71) | 126.63 (106.49,148.03) | -0.32 (-0.36 - -0.28) |
|  | Male | 17100 (7579,30612) | 21170 (9764,36591) |  | 6.48 (2.87,11.6) | 6.61 (3.05,11.42) | 0.07 (0.04 - 0.10) | 268550 (219437,321608) | 287768 (240726,336069) |  | 101.77 (83.16,121.87) | 89.84 (75.15,104.91) | -0.40 (-0.42 - -0.38) |

**AAPC Average Annual Percent Change, ASR Age-Standardized Rate, UI uncertainty interval, CI confidence interval.**

**Table S5 Global Death and DALYs of Ischemic stroke and their AAPCs from 1990 to 2021 by sex and age groups.**

| **Age Group** | **Sex** | **Death (95% UI)** | | | | | | **DALYs (95% UI)** | | | | | |
| --- | --- | --- | --- | --- | --- | --- | --- | --- | --- | --- | --- | --- | --- |
|  |  | **Number** | |  | **ASR** | | | **Number** | |  | **ASR** | | |
|  |  | **1990** | **2021** |  | **1990** | **2021** | **AAPC (95% CI)** | **1990** | **2021** |  | **1990** | **2021** | **AAPC (95% CI)** |
| <5 | Both | 4775 (3320,7666) | 1841 (1199,2702) |  | 0.77 (0.54,1.24) | 0.28 (0.18,0.41) | -3.23 (-3.32 - -3.14) | 441840 (314779,698108) | 179218 (121507,254957) |  | 71.27 (50.78,112.61) | 27.23 (18.46,38.74) | -3.07 (-3.16 - -2.99) |
|  | Female | 2351 (1535,4079) | 620 (447,1028) |  | 0.78 (0.51,1.36) | 0.19 (0.14,0.32) | -4.42 (-4.66 - -4.18) | 219866 (147125,374149) | 65585 (50074,102123) |  | 73.19 (48.98,124.55) | 20.61 (15.74,32.09) | -4.04 (-4.27 - -3.80) |
|  | Male | 2425 (1647,3632) | 1221 (657,1867) |  | 0.76 (0.52,1.14) | 0.36 (0.19,0.55) | -2.35 (-2.47 - -2.23) | 221974 (153713,328115) | 113633 (63772,170527) |  | 69.47 (48.1,102.68) | 33.42 (18.76,50.16) | -2.30 (-2.42 - -2.19) |
| 5-9 | Both | 699 (543,983) | 405 (325,484) |  | 0.12 (0.09,0.17) | 0.06 (0.05,0.07) | -2.26 (-2.60 - -1.92) | 104313 (84167,130724) | 79640 (64413,97380) |  | 17.88 (14.42,22.4) | 11.59 (9.38,14.17) | -1.37 (-1.48 - -1.26) |
|  | Female | 342 (246,512) | 135 (104,175) |  | 0.12 (0.09,0.18) | 0.04 (0.03,0.05) | -3.39 (-3.79 - -3.00) | 56695 (44027,73887) | 40465 (30738,50739) |  | 19.98 (15.51,26.03) | 12.17 (9.24,15.26) | -1.57 (-1.72 - -1.41) |
|  | Male | 357 (265,473) | 269 (187,346) |  | 0.12 (0.09,0.16) | 0.08 (0.05,0.1) | -1.41 (-1.54 - -1.28) | 47618 (38376,59273) | 39176 (30634,47863) |  | 15.89 (12.8,19.78) | 11.05 (8.64,13.5) | -1.16 (-1.24 - -1.07) |
| 10-14 | Both | 406 (335,515) | 316 (261,378) |  | 0.08 (0.06,0.1) | 0.05 (0.04,0.06) | -1.46 (-1.66 - -1.26) | 100529 (77889,127928) | 97561 (74083,123766) |  | 18.77 (14.54,23.88) | 14.63 (11.11,18.57) | -0.80 (-0.85 - -0.75) |
|  | Female | 229 (173,315) | 154 (122,203) |  | 0.09 (0.07,0.12) | 0.05 (0.04,0.06) | -1.87 (-2.10 - -1.64) | 59828 (45636,77458) | 57310 (43050,73372) |  | 22.88 (17.46,29.63) | 17.75 (13.33,22.72) | -0.81 (-0.86 - -0.76) |
|  | Male | 177 (145,222) | 161 (123,203) |  | 0.06 (0.05,0.08) | 0.05 (0.04,0.06) | -1.05 (-1.30 - -0.81) | 40701 (31329,51912) | 40251 (31251,51089) |  | 14.84 (11.42,18.93) | 11.71 (9.09,14.86) | -0.77 (-0.83 - -0.70) |
| 15-19 | Both | 1142 (996,1302) | 975 (824,1126) |  | 0.22 (0.19,0.25) | 0.16 (0.13,0.18) | -1.08 (-1.29 - -0.88) | 179662 (147590,215185) | 168578 (137679,203194) |  | 34.59 (28.41,41.43) | 27.02 (22.06,32.56) | -0.80 (-0.87 - -0.72) |
|  | Female | 624 (503,740) | 515 (417,640) |  | 0.24 (0.2,0.29) | 0.17 (0.14,0.21) | -1.14 (-1.33 - -0.95) | 104491 (84929,127903) | 96915 (78712,118217) |  | 40.89 (33.24,50.05) | 31.92 (25.92,38.93) | -0.80 (-0.86 - -0.73) |
|  | Male | 518 (440,633) | 460 (373,557) |  | 0.2 (0.17,0.24) | 0.14 (0.12,0.17) | -1.00 (-1.15 - -0.86) | 75170 (61447,90513) | 71663 (57759,86601) |  | 28.49 (23.29,34.3) | 22.37 (18.03,27.03) | -0.78 (-0.89 - -0.66) |

**AAPC Average Annual Percent Change, DALYs Disability-Adjusted Life Years, ASR Age-Standardized Rate, UI uncertainty interval, CI confidence interval.**

**Table S6 AAPCs of Global Burden of Ischemic Stroke from 2015 to 2021 by Sex and Age Group.**

| **Age Group** | **Sex** | **AAPC (95% CI)** | | | |
| --- | --- | --- | --- | --- | --- |
|  |  | **Incidence** | **Prevalence** | **Death** | **DALYs** |
| <5 | Both | 1.48 (0.74 - 2.23) | 0.26 (0.11 - 0.42) | -5.70 (-6.31 - -5.09) | -5.28 (-5.85 - -4.70) |
|  | Female | 1.81 (1.02 - 2.61) | 0.45 (0.29 - 0.60) | -6.84 (-7.63 - -6.05) | -5.92 (-6.55 - -5.28) |
|  | Male | 0.97 (0.31 - 1.63) | -0.05 (-0.24 - 0.14) | -5.22 (-5.57 - -4.88) | -5.02 (-5.35 - -4.70) |
| 5-9 | Both | 2.01 (0.83 - 3.19) | -0.12 (-0.17 - -0.06) | -5.04 (-5.83 - -4.25) | -2.31 (-2.55 - -2.08) |
|  | Female | 2.37 (1.07 - 3.68) | 0.05 (0.02 - 0.08) | -5.41 (-6.44 - -4.36) | -1.65 (-2.10 - -1.19) |
|  | Male | 1.51 (0.49 - 2.54) | -0.34 (-0.48 - -0.21) | -4.66 (-5.13 - -4.18) | -2.94 (-3.15 - -2.73) |
| 10-14 | Both | 1.62 (0.81 - 2.45) | 0.01 (-0.02 - 0.04) | -3.30 (-3.62 - -2.98) | -0.86 (-0.97 - -0.76) |
|  | Female | 1.89 (1.00 - 2.78) | 0.14 (0.10 - 0.18) | -3.39 (-3.85 - -2.92) | -0.66 (-0.74 - -0.58) |
|  | Male | 1.29 (0.57 - 2.02) | -0.20 (-0.28 - -0.12) | -3.23 (-3.55 - -2.91) | -1.17 (-1.31 - -1.03) |
| 15-19 | Both | 1.16 (0.73 - 1.60) | 0.09 (0.07 - 0.12) | -1.53 (-1.60 - -1.45) | -0.66 (-0.80 - -0.53) |
|  | Female | 1.29 (0.81 - 1.77) | 0.18 (0.16 - 0.21) | -1.49 (-1.60 - -1.38) | -0.54 (-0.63 - -0.45) |
|  | Male | 1.02 (0.48 - 1.57) | -0.04 (-0.05 - -0.03) | -1.56 (-1.71 - -1.42) | -0.80 (-0.91 - -0.68) |

**AAPC Average Annual Percent Change, DALYs Disability-Adjusted Life Years, ASR Age-Standardized Rate, CI confidence interval.**

**Table S7 Ischemic Stroke Burden Across 21 Regions in 2021, and the Average Annual Percent Change in ASR from 1990 to 2021.**

| **Location** | **Sex** | **Incidence** | | | **Prevalence** | | | **Death** | | | **DALYs** | | |  |
| --- | --- | --- | --- | --- | --- | --- | --- | --- | --- | --- | --- | --- | --- | --- |
|  |  | **Number in 2021 (95%UI)** | **ASR in 2021 (per 100,000, 95%UI)** | **Average Annual Percent Change (95% CI)** | **Number in 2021 (95%UI)** | **ASR in 2021 (per 100,000, 95%UI)** | **Average Annual Percent Change (95% CI)** | **Number in 2021 (95%UI)** | **ASR in 2021 (per 100,000, 95%UI)** | **Average Annual Percent Change (95% CI)** | **Number in 2021 (95%UI)** | **ASR in 2021 (per 100,000, 95%UI)** | **Average Annual Percent Change (95% CI)** |  |
| Andean Latin America | Both | 1213 (624,2078) | 5.14 (2.11,9.59) | -0.49 (-0.58 - -0.40) | 10640 (9276,12058) | 45.51 (38.98,52.13) | -0.52 (-0.54 - -0.51) | 21 (17,26) | 0.09 (0.06,0.12) | -4.61 (-5.12 - -4.10) | 2607 (2132,3125) | 11.08 (8.54,14.05) | -3.80 (-4.16 - -3.43) | |
|  | Female | 659 (346,1177) | 5.77 (2.37,10.89) | -0.43 (-0.50 - -0.36) | 5784 (5043,6540) | 51.25 (43.89,58.77) | -0.45 (-0.46 - -0.44) | 10 (8,13) | 0.09 (0.06,0.13) | -4.13 (-4.48 - -3.79) | 1348 (1073,1641) | 11.89 (8.87,15.56) | -3.26 (-3.58 - -2.94) | |
|  | Male | 553 (284,924) | 4.54 (1.79,8.7) | -0.52 (-0.66 - -0.37) | 4856 (4215,5518) | 40.16 (34.32,46.16) | -0.58 (-0.61 - -0.56) | 11 (8,14) | 0.09 (0.06,0.12) | -4.93 (-5.30 - -4.56) | 1259 (1014,1538) | 10.34 (7.63,13.5) | -4.28 (-4.55 - -4.01) | |
| Australasia | Both | 312 (182,465) | 4.16 (1.91,7.25) | -0.23 (-0.26 - -0.19) | 3074 (2672,3493) | 40.44 (34.54,46.13) | -0.26 (-0.28 - -0.24) | 1 (0,1) | 0.01 (0.01,0.01) | -6.52 (-7.63 - -5.40) | 467 (337,609) | 6.17 (4.41,8.08) | -1.93 (-2.12 - -1.73) | |
|  | Female | 139 (72,229) | 3.82 (1.54,7.14) | -0.34 (-0.41 - -0.26) | 1462 (1241,1700) | 39.59 (33.16,46.36) | -0.26 (-0.28 - -0.24) | 0 (0,0) | 0.01 (0,0.01) | -6.09 (-7.19 - -4.98) | 242 (173,321) | 6.57 (4.64,8.74) | -1.38 (-1.73 - -1.02) | |
|  | Male | 173 (105,256) | 4.47 (2.1,7.64) | -0.15 (-0.23 - -0.07) | 1612 (1414,1814) | 41.24 (35.64,46.97) | -0.27 (-0.29 - -0.25) | 0 (0,0) | 0.01 (0.01,0.01) | -7.33 (-9.16 - -5.46) | 225 (165,292) | 5.79 (4.19,7.58) | -2.38 (-2.72 - -2.05) | |
| Caribbean | Both | 912 (513,1491) | 5.98 (2.81,10.79) | -0.32 (-0.47 - -0.18) | 9353 (8304,10426) | 61.14 (53.76,68.88) | -0.32 (-0.34 - -0.30) | 42 (25,71) | 0.28 (0.15,0.5) | -1.51 (-1.81 - -1.21) | 4377 (2865,6878) | 28.8 (17.83,47.8) | -1.42 (-1.66 - -1.17) | |
|  | Female | 556 (331,922) | 7.42 (3.53,13.11) | -0.19 (-0.25 - -0.14) | 5654 (5069,6327) | 75.03 (66.29,84.54) | -0.23 (-0.25 - -0.22) | 25 (16,47) | 0.34 (0.19,0.65) | -1.39 (-1.63 - -1.14) | 2646 (1784,4512) | 35.4 (22.11,62.27) | -1.29 (-1.51 - -1.06) | |
|  | Male | 355 (185,600) | 4.59 (1.92,8.66) | -0.45 (-0.59 - -0.32) | 3699 (3238,4145) | 47.66 (41.42,53.94) | -0.42 (-0.44 - -0.40) | 17 (8,31) | 0.22 (0.09,0.42) | -1.68 (-1.97 - -1.40) | 1732 (918,2894) | 22.4 (10.75,39.53) | -1.59 (-1.85 - -1.32) | |
| Central Asia | Both | 2183 (1211,3717) | 6.5 (2.88,11.99) | -0.36 (-0.44 - -0.29) | 17172 (15039,19388) | 53.68 (46.09,61.65) | -0.40 (-0.41 - -0.38) | 20 (17,24) | 0.07 (0.05,0.08) | -2.42 (-3.32 - -1.51) | 4753 (3768,5905) | 14.97 (11.45,18.82) | -1.28 (-1.72 - -0.83) | |
|  | Female | 1337 (745,2339) | 8.24 (3.75,15.22) | -0.16 (-0.24 - -0.09) | 10584 (9344,11933) | 68.4 (58.96,78.27) | -0.16 (-0.18 - -0.13) | 9 (7,11) | 0.06 (0.05,0.08) | -2.64 (-3.75 - -1.52) | 2766 (2121,3486) | 17.96 (13.12,23.3) | -1.04 (-1.44 - -0.64) | |
|  | Male | 846 (429,1460) | 4.87 (1.91,9.25) | -0.64 (-0.70 - -0.57) | 6588 (5680,7487) | 39.88 (33.97,46.14) | -0.74 (-0.76 - -0.71) | 11 (9,13) | 0.07 (0.06,0.09) | -2.19 (-3.10 - -1.27) | 1987 (1600,2407) | 12.16 (9.55,15.04) | -1.51 (-2.04 - -0.97) | |
| Central Europe | Both | 1208 (601,2121) | 5.1 (2.05,9.72) | -0.60 (-0.74 - -0.46) | 11113 (9278,12942) | 46.11 (38.09,54.59) | -0.51 (-0.53 - -0.49) | 5 (4,5) | 0.02 (0.02,0.02) | -5.06 (-5.72 - -4.39) | 2438 (1760,3115) | 10.14 (7.29,13.28) | -1.84 (-2.08 - -1.60) | |
|  | Female | 739 (376,1304) | 6.44 (2.63,12.12) | -0.51 (-0.64 - -0.37) | 6938 (5822,8073) | 59.39 (49.21,70.07) | -0.41 (-0.43 - -0.40) | 2 (2,2) | 0.02 (0.02,0.02) | -5.00 (-5.66 - -4.34) | 1520 (1084,1980) | 13.04 (9.1,17.55) | -1.45 (-1.68 - -1.21) | |
|  | Male | 469 (225,819) | 3.84 (1.41,7.63) | -0.76 (-0.86 - -0.67) | 4175 (3470,4873) | 33.61 (27.62,40.02) | -0.65 (-0.66 - -0.64) | 3 (2,3) | 0.02 (0.02,0.03) | -5.16 (-5.93 - -4.38) | 918 (683,1173) | 7.42 (5.46,9.59) | -2.36 (-2.66 - -2.07) | |
| Central Latin America | Both | 4580 (2360,7983) | 5.36 (2.21,10.12) | -0.96 (-1.09 - -0.83) | 45411 (37929,53692) | 51.91 (42.6,61.77) | -0.84 (-0.86 - -0.81) | 46 (38,55) | 0.05 (0.04,0.07) | -4.07 (-4.72 - -3.42) | 7465 (6149,8891) | 8.65 (6.91,10.67) | -2.90 (-3.25 - -2.54) | |
|  | Female | 2678 (1417,4797) | 6.36 (2.68,11.91) | -0.95 (-1.07 - -0.83) | 25976 (21629,30751) | 60.17 (49.3,71.67) | -0.81 (-0.82 - -0.80) | 21 (18,25) | 0.05 (0.04,0.06) | -4.25 (-5.58 - -2.89) | 4048 (3287,4906) | 9.47 (7.47,11.77) | -2.64 (-3.29 - -1.98) | |
|  | Male | 1902 (908,3330) | 4.4 (1.67,8.6) | -0.95 (-1.10 - -0.80) | 19435 (16244,23071) | 43.87 (36.01,52.18) | -0.86 (-0.90 - -0.82) | 25 (20,31) | 0.06 (0.05,0.07) | -3.72 (-4.43 - -3.01) | 3417 (2840,4064) | 7.84 (6.33,9.59) | -2.93 (-3.31 - -2.56) | |
| Central Sub-Saharan Africa | Both | 4882 (2666,8339) | 6.65 (2.94,12.21) | -0.62 (-0.76 - -0.49) | 42666 (37457,48385) | 61.8 (53.34,70.66) | -0.65 (-0.67 - -0.63) | 87 (48,137) | 0.12 (0.06,0.2) | -4.87 (-4.97 - -4.78) | 12811 (8904,17518) | 17.57 (11.17,25.75) | -3.72 (-3.84 - -3.59) | |
|  | Female | 2777 (1556,4859) | 7.61 (3.34,14.1) | -0.53 (-0.69 - -0.37) | 24931 (21847,28307) | 72.67 (62.69,83.47) | -0.54 (-0.56 - -0.51) | 38 (20,63) | 0.11 (0.05,0.19) | -4.90 (-5.06 - -4.75) | 6521 (4524,9039) | 18.46 (11.65,28.42) | -3.45 (-3.62 - -3.28) | |
|  | Male | 2105 (1119,3567) | 5.71 (2.43,10.71) | -0.75 (-0.85 - -0.64) | 17735 (15547,20141) | 51.12 (43.96,58.64) | -0.81 (-0.83 - -0.78) | 49 (23,84) | 0.13 (0.05,0.23) | -4.85 (-5.06 - -4.65) | 6290 (3914,9343) | 16.67 (9.58,26.06) | -3.98 (-4.15 - -3.80) | |
| East Asia | Both | 22831 (12070,40012) | 6.64 (2.84,12.41) | -0.41 (-0.54 - -0.29) | 211419 (171911,258376) | 61.61 (48.97,76.61) | -0.69 (-0.71 - -0.66) | 171 (132,209) | 0.05 (0.04,0.07) | -4.12 (-4.39 - -3.85) | 53889 (40476,68844) | 15.85 (11.6,20.59) | -2.03 (-2.19 - -1.87) | |
|  | Female | 12844 (6865,22548) | 8.01 (3.48,14.98) | -0.43 (-0.69 - -0.18) | 120691 (98348,147226) | 75.43 (59.9,93.47) | -0.63 (-0.64 - -0.62) | 63 (48,81) | 0.04 (0.03,0.05) | -4.79 (-5.10 - -4.47) | 29527 (21343,38345) | 18.57 (13.03,24.83) | -1.93 (-2.06 - -1.80) | |
|  | Male | 9986 (4995,17697) | 5.45 (2.26,10.36) | -0.38 (-0.43 - -0.32) | 90728 (73628,111234) | 49.55 (39.13,61.47) | -0.71 (-0.74 - -0.67) | 109 (83,137) | 0.06 (0.04,0.08) | -3.59 (-3.71 - -3.47) | 24362 (18535,31080) | 13.47 (10.06,17.57) | -2.18 (-2.28 - -2.09) | |
| Eastern Europe | Both | 2785 (1468,4803) | 6.02 (2.59,11.23) | -0.53 (-0.66 - -0.40) | 24218 (19560,29690) | 51.82 (40.94,64.57) | -0.44 (-0.46 - -0.42) | 11 (10,12) | 0.02 (0.02,0.03) | -4.31 (-5.59 - -3.01) | 5350 (3846,7017) | 11.54 (8.26,15.46) | -1.39 (-1.75 - -1.03) | |
|  | Female | 1814 (984,3192) | 8.06 (3.54,14.96) | -0.35 (-0.49 - -0.21) | 16237 (13182,19883) | 71.33 (56.34,88.48) | -0.29 (-0.31 - -0.27) | 5 (5,6) | 0.02 (0.02,0.03) | -4.45 (-5.44 - -3.44) | 3560 (2500,4731) | 15.72 (10.87,21.51) | -1.10 (-1.38 - -0.82) | |
|  | Male | 971 (461,1707) | 4.09 (1.46,7.99) | -0.81 (-1.03 - -0.58) | 7980 (6383,9755) | 33.32 (25.94,41.7) | -0.71 (-0.74 - -0.69) | 6 (5,6) | 0.02 (0.02,0.03) | -4.21 (-5.69 - -2.70) | 1789 (1340,2303) | 7.58 (5.62,9.97) | -1.95 (-2.61 - -1.29) | |
| Eastern Sub-Saharan Africa | Both | 15743 (8737,26900) | 6.95 (3.14,12.62) | -0.32 (-0.47 - -0.17) | 138039 (119389,158445) | 63.52 (54.18,73.27) | -0.34 (-0.34 - -0.33) | 215 (162,284) | 0.1 (0.07,0.13) | -2.10 (-2.22 - -1.99) | 35335 (29000,42946) | 15.86 (12.24,20.24) | -1.37 (-1.48 - -1.26) | |
|  | Female | 8686 (4917,14713) | 7.71 (3.49,14.02) | -0.30 (-0.48 - -0.13) | 79681 (69401,91379) | 73.78 (63.17,85) | -0.32 (-0.33 - -0.31) | 113 (72,164) | 0.1 (0.06,0.15) | -2.22 (-2.32 - -2.12) | 20069 (15678,25168) | 18.26 (13.31,24.09) | -1.37 (-1.48 - -1.27) | |
|  | Male | 7058 (3831,12191) | 6.21 (2.71,11.43) | -0.34 (-0.46 - -0.21) | 58357 (50143,67136) | 53.46 (45.13,62.11) | -0.36 (-0.37 - -0.34) | 102 (66,145) | 0.09 (0.05,0.14) | -1.99 (-2.12 - -1.85) | 15266 (11541,19768) | 13.51 (9.64,18.48) | -1.38 (-1.52 - -1.24) | |
| High-income Asia Pacific | Both | 1459 (665,2662) | 4.74 (1.75,9.33) | -0.24 (-0.28 - -0.21) | 15056 (12426,17992) | 46.19 (37.46,55.51) | -0.22 (-0.24 - -0.20) | 3 (3,4) | 0.01 (0.01,0.01) | -6.45 (-6.95 - -5.96) | 2760 (1951,3632) | 8.48 (5.95,11.36) | -1.70 (-1.85 - -1.54) | |
|  | Female | 811 (371,1493) | 5.42 (2.04,10.79) | -0.16 (-0.22 - -0.10) | 8558 (7084,10156) | 54.05 (43.97,64.76) | -0.14 (-0.16 - -0.11) | 2 (1,2) | 0.01 (0.01,0.01) | -5.99 (-6.78 - -5.19) | 1646 (1172,2191) | 10.41 (7.26,14.13) | -1.31 (-1.46 - -1.17) | |
|  | Male | 649 (291,1160) | 4.09 (1.45,8.26) | -0.36 (-0.42 - -0.30) | 6498 (5321,7818) | 38.75 (31.17,46.91) | -0.32 (-0.33 - -0.30) | 2 (1,2) | 0.01 (0.01,0.01) | -6.99 (-7.68 - -6.28) | 1114 (789,1491) | 6.66 (4.68,8.98) | -2.21 (-2.40 - -2.01) | |
| High-income North America | Both | 6552 (3549,11040) | 7.41 (3.29,13.5) | -0.98 (-1.10 - -0.86) | 94943 (76589,114974) | 102.01 (80.78,125.67) | -0.75 (-0.97 - -0.53) | 35 (31,38) | 0.04 (0.04,0.05) | -4.29 (-5.44 - -3.13) | 15812 (11897,20255) | 17.34 (12.81,22.6) | -1.99 (-2.32 - -1.66) | |
|  | Female | 3213 (1720,5444) | 7.44 (3.31,13.69) | -0.92 (-1.07 - -0.78) | 48438 (38680,58823) | 106.5 (84.02,132.17) | -0.61 (-0.85 - -0.37) | 16 (14,17) | 0.04 (0.03,0.04) | -4.39 (-5.77 - -2.98) | 8511 (6314,11024) | 19.04 (13.78,25.09) | -1.87 (-2.40 - -1.34) | |
|  | Male | 3339 (1846,5642) | 7.38 (3.28,13.45) | -1.04 (-1.18 - -0.90) | 46505 (37787,56431) | 97.71 (77.6,119.98) | -0.81 (-0.99 - -0.63) | 19 (17,21) | 0.04 (0.04,0.05) | -4.30 (-5.45 - -3.14) | 7301 (5511,9318) | 15.71 (11.64,20.28) | -2.24 (-2.57 - -1.90) | |
| North Africa and Middle East | Both | 22461 (13754,35906) | 9.57 (4.9,16.58) | -0.50 (-0.63 - -0.38) | 201403 (178996,224164) | 87.1 (76.52,97.99) | -0.64 (-0.67 - -0.61) | 820 (650,1037) | 0.35 (0.26,0.48) | -3.89 (-4.07 - -3.71) | 94399 (77924,116019) | 40.61 (31.93,52.09) | -3.37 (-3.52 - -3.23) | |
|  | Female | 12058 (7468,19271) | 10.63 (5.47,18.43) | -0.55 (-0.68 - -0.42) | 105844 (94013,117804) | 94.69 (82.86,106.56) | -0.69 (-0.72 - -0.66) | 417 (310,562) | 0.37 (0.26,0.54) | -4.43 (-4.65 - -4.20) | 49481 (39423,63278) | 44.04 (33.35,59.55) | -3.79 (-3.94 - -3.63) | |
|  | Male | 10403 (6310,16802) | 8.58 (4.3,14.86) | -0.43 (-0.56 - -0.30) | 95559 (84886,106574) | 80 (70.28,90.19) | -0.58 (-0.60 - -0.56) | 403 (313,504) | 0.34 (0.24,0.47) | -3.30 (-3.46 - -3.13) | 44918 (36706,54182) | 37.39 (28.25,48.83) | -2.89 (-3.03 - -2.76) | |
| Oceania | Both | 518 (313,812) | 7.76 (3.9,13.29) | -0.33 (-0.39 - -0.27) | 5215 (4712,5743) | 86.57 (77.03,96.26) | -0.33 (-0.35 - -0.32) | 28 (15,53) | 0.39 (0.2,0.78) | -0.30 (-0.80 - 0.20) | 3432 (2232,5635) | 49.92 (29.49,87.58) | -0.32 (-0.67 - 0.03) | |
|  | Female | 283 (172,449) | 8.92 (4.42,15.55) | -0.30 (-0.34 - -0.27) | 2816 (2515,3112) | 98.45 (87.15,110.03) | -0.30 (-0.31 - -0.30) | 14 (7,28) | 0.42 (0.19,0.91) | -0.66 (-0.92 - -0.40) | 1803 (1147,3021) | 55.98 (30.49,102.38) | -0.58 (-0.76 - -0.40) | |
|  | Male | 236 (143,359) | 6.71 (3.39,11.63) | -0.34 (-0.43 - -0.26) | 2399 (2155,2651) | 75.8 (67.18,84.58) | -0.36 (-0.37 - -0.34) | 14 (7,28) | 0.36 (0.16,0.74) | 0.13 (-0.51 - 0.78) | 1629 (958,2828) | 44.44 (23.57,82.12) | -0.01 (-0.49 - 0.46) | |
| South Asia | Both | 33773 (16475,59985) | 4.9 (1.97,9.39) | -0.20 (-0.44 - 0.04) | 284158 (229808,349854) | 40.2 (31.62,49.98) | -0.25 (-0.28 - -0.22) | 520 (385,782) | 0.08 (0.05,0.12) | -2.83 (-3.15 - -2.51) | 83653 (66659,106522) | 12.31 (8.92,16.89) | -1.92 (-2.03 - -1.80) | |
|  | Female | 19933 (10176,35531) | 6.03 (2.5,11.41) | -0.02 (-0.24 - 0.21) | 167617 (136191,205616) | 49.34 (38.91,60.92) | -0.08 (-0.11 - -0.06) | 282 (182,511) | 0.09 (0.05,0.16) | -2.98 (-3.30 - -2.66) | 48633 (37787,66714) | 14.77 (10.38,22.07) | -1.88 (-2.02 - -1.75) | |
|  | Male | 13839 (6324,24435) | 3.86 (1.36,7.83) | -0.40 (-0.46 - -0.34) | 116541 (92469,144390) | 31.75 (24.71,39.74) | -0.46 (-0.49 - -0.43) | 238 (168,338) | 0.07 (0.04,0.11) | -2.67 (-3.07 - -2.28) | 35020 (26929,44400) | 10.03 (6.76,13.81) | -1.98 (-2.11 - -1.85) | |
| Southeast Asia | Both | 14560 (8083,24741) | 6.34 (2.84,11.64) | -0.38 (-0.44 - -0.33) | 129665 (109200,153279) | 55.99 (46.04,66.82) | -0.51 (-0.54 - -0.49) | 324 (225,425) | 0.14 (0.1,0.19) | -2.22 (-2.48 - -1.97) | 50784 (39198,61869) | 22.19 (16.13,28.67) | -1.59 (-1.74 - -1.43) | |
|  | Female | 8516 (4816,14752) | 7.63 (3.47,14) | -0.30 (-0.36 - -0.24) | 75776 (63790,89185) | 67.24 (55.42,80.35) | -0.43 (-0.46 - -0.41) | 148 (107,203) | 0.13 (0.09,0.19) | -2.72 (-2.97 - -2.46) | 27265 (21195,34313) | 24.45 (17.69,32.53) | -1.69 (-1.83 - -1.56) | |
|  | Male | 6044 (3213,10352) | 5.13 (2.15,9.51) | -0.47 (-0.55 - -0.38) | 53889 (45377,63771) | 45.33 (37.23,54.29) | -0.60 (-0.62 - -0.58) | 176 (113,233) | 0.15 (0.09,0.21) | -1.77 (-1.99 - -1.54) | 23519 (17392,29206) | 20.04 (13.77,26.23) | -1.44 (-1.52 - -1.37) | |
| Southern Latin America | Both | 868 (413,1582) | 4.46 (1.71,8.71) | -0.57 (-0.63 - -0.50) | 8708 (7551,9974) | 43.1 (36.7,49.89) | -0.36 (-0.38 - -0.34) | 4 (4,5) | 0.02 (0.02,0.03) | -5.73 (-6.74 - -4.71) | 1576 (1202,2034) | 7.86 (5.88,10.25) | -2.59 (-2.74 - -2.44) | |
|  | Female | 463 (223,861) | 4.86 (1.87,9.47) | -0.45 (-0.54 - -0.36) | 4779 (4132,5526) | 48.22 (41.16,55.85) | -0.23 (-0.26 - -0.21) | 2 (2,2) | 0.02 (0.02,0.03) | -6.03 (-6.54 - -5.51) | 893 (657,1186) | 9.08 (6.53,12.23) | -2.42 (-2.57 - -2.27) | |
|  | Male | 405 (191,727) | 4.07 (1.47,8.11) | -0.66 (-0.71 - -0.62) | 3929 (3367,4490) | 38.17 (32.22,44.46) | -0.51 (-0.52 - -0.49) | 2 (2,3) | 0.02 (0.02,0.03) | -5.11 (-5.97 - -4.24) | 683 (526,860) | 6.68 (5.11,8.46) | -2.78 (-3.10 - -2.45) | |
| Southern Sub-Saharan Africa | Both | 2274 (1262,3974) | 7.28 (3.22,13.28) | -0.55 (-0.64 - -0.45) | 21140 (17647,24968) | 68.57 (56.3,82.11) | -0.63 (-0.66 - -0.60) | 26 (20,33) | 0.08 (0.06,0.11) | -1.71 (-2.29 - -1.12) | 4805 (3893,5922) | 15.56 (11.87,20.12) | -1.16 (-1.44 - -0.89) | |
|  | Female | 1454 (807,2472) | 9.38 (4.29,16.88) | -0.59 (-0.70 - -0.49) | 13936 (11645,16427) | 90.98 (75.11,108.58) | -0.68 (-0.72 - -0.65) | 13 (10,18) | 0.09 (0.06,0.13) | -2.00 (-2.99 - -1.00) | 2968 (2327,3729) | 19.34 (14.08,25.64) | -1.27 (-1.63 - -0.90) | |
|  | Male | 820 (398,1440) | 5.22 (2.02,10.11) | -0.40 (-0.58 - -0.23) | 7204 (5933,8602) | 46.48 (37.59,56.22) | -0.44 (-0.46 - -0.42) | 12 (9,16) | 0.08 (0.06,0.11) | -1.25 (-1.61 - -0.89) | 1838 (1490,2256) | 11.84 (8.93,15.08) | -1.00 (-1.18 - -0.81) | |
| Tropical Latin America | Both | 2594 (1242,4542) | 3.89 (1.45,7.64) | -1.15 (-1.30 - -1.00) | 24606 (19799,30205) | 37.01 (29,46.05) | -0.91 (-0.94 - -0.89) | 24 (21,26) | 0.04 (0.03,0.04) | -3.81 (-4.65 - -2.96) | 3928 (3257,4763) | 5.89 (4.78,7.24) | -2.70 (-3.23 - -2.16) | |
|  | Female | 1433 (677,2544) | 4.39 (1.62,8.52) | -1.06 (-1.36 - -0.76) | 14086 (11346,17241) | 43.18 (33.94,53.97) | -0.77 (-0.80 - -0.75) | 12 (10,13) | 0.04 (0.03,0.04) | -3.59 (-4.25 - -2.91) | 2185 (1751,2688) | 6.68 (5.26,8.32) | -2.33 (-2.62 - -2.04) | |
|  | Male | 1161 (556,2069) | 3.42 (1.22,6.87) | -1.14 (-1.19 - -1.09) | 10519 (8383,12929) | 31.07 (24.23,38.7) | -1.08 (-1.10 - -1.05) | 12 (11,14) | 0.04 (0.03,0.04) | -4.23 (-5.07 - -3.38) | 1743 (1487,2066) | 5.13 (4.2,6.22) | -3.24 (-3.97 - -2.51) | |
| Western Europe | Both | 3936 (1898,6761) | 4.31 (1.71,8.15) | -0.95 (-1.10 - -0.81) | 42225 (35759,48911) | 44.68 (37.23,52.38) | -0.68 (-0.71 - -0.65) | 8 (7,9) | 0.01 (0.01,0.01) | -7.91 (-8.62 - -7.19) | 6178 (4499,8088) | 6.57 (4.73,8.7) | -3.02 (-3.13 - -2.91) | |
|  | Female | 2072 (1005,3509) | 4.67 (1.89,8.84) | -0.93 (-1.02 - -0.84) | 23002 (19653,26636) | 50.1 (41.84,58.89) | -0.66 (-0.68 - -0.63) | 4 (4,5) | 0.01 (0.01,0.01) | -7.68 (-8.51 - -6.84) | 3582 (2602,4690) | 7.84 (5.64,10.41) | -2.61 (-2.71 - -2.51) | |
|  | Male | 1864 (899,3252) | 3.96 (1.53,7.65) | -0.98 (-1.10 - -0.85) | 19223 (16153,22277) | 39.56 (32.64,46.54) | -0.71 (-0.75 - -0.68) | 4 (3,4) | 0.01 (0.01,0.01) | -8.46 (-9.23 - -7.67) | 2597 (1904,3398) | 5.37 (3.86,7.07) | -3.49 (-3.64 - -3.33) | |
| Western Sub-Saharan Africa | Both | 27986 (17516,44333) | 10.36 (5.47,17.58) | -0.28 (-0.39 - -0.18) | 248125 (216147,283215) | 99.11 (85.14,113.81) | -0.32 (-0.34 - -0.30) | 1125 (555,1785) | 0.38 (0.19,0.61) | -1.27 (-1.40 - -1.14) | 128179 (79173,183615) | 45.36 (27.39,66.34) | -1.06 (-1.14 - -0.98) | |
|  | Female | 16364 (10357,25642) | 11.78 (6.23,19.81) | -0.14 (-0.26 - -0.02) | 162804 (142241,185310) | 127.6 (110.31,146.17) | -0.18 (-0.20 - -0.17) | 228 (134,421) | 0.16 (0.1,0.31) | -1.29 (-1.40 - -1.18) | 41063 (30839,57695) | 30.77 (22.17,45.96) | -0.75 (-0.82 - -0.67) | |
|  | Male | 11622 (7041,18983) | 9.03 (4.49,15.58) | -0.46 (-0.51 - -0.40) | 85320 (72942,98245) | 70.37 (58.97,82.4) | -0.54 (-0.56 - -0.51) | 897 (380,1439) | 0.6 (0.25,0.98) | -1.30 (-1.42 - -1.17) | 87117 (42675,134156) | 59.45 (29.3,92.62) | -1.22 (-1.34 - -1.11) | |

**DALYs Disability-Adjusted Life Years, ASR Age-Standardized Rate, UI uncertainty interval, CI confidence interval.**

**Table S8 Ischemic Stroke Burden Across 204 countries in 2021, and the Average Annual Percent Change in ASR from 1990 to 2021.**

| **Location** | **Sex** | **Incidence** | | | **Prevalence** | | | **Death** | | | **DALYs** | | |
| --- | --- | --- | --- | --- | --- | --- | --- | --- | --- | --- | --- | --- | --- |
|  |  | **Number in 2021 (95%UI)** | **ASR in 2021 (per 100,000, 95%UI)** | **Average Annual Percent Change (95% CI)** | **Number in 2021 (95%UI)** | **ASR in 2021 (per 100,000, 95%UI)** | **Average Annual Percent Change (95% CI)** | **Number in 2021 (95%UI)** | **ASR in 2021 (per 100,000, 95%UI)** | **Average Annual Percent Change (95% CI)** | **Number in 2021 (95%UI)** | **ASR in 2021 (per 100,000, 95%UI)** | **Average Annual Percent Change (95% CI)** |
| Afghanistan | Both | 1650 (1021,2661) | 9.36 (4.8,16.19) | -0.63 (-0.80 - -0.47) | 13287 (11909,14804) | 81.53 (71.9,91.84) | -0.69 (-0.74 - -0.65) | 90 (54,143) | 0.49 (0.26,0.91) | -1.73 (-2.18 - -1.28) | 4751 (2819,7586) | 51.41 (30.73,86.41) | -1.64 (-2.01 - -1.27) |
|  | Female | 860 (540,1414) | 10.19 (5.12,18.09) | -0.58 (-0.72 - -0.45) | 6820 (6028,7670) | 86.86 (75.69,99.15) | -0.62 (-0.66 - -0.58) | 43 (24,80) | 0.51 (0.24,1.09) | -2.16 (-2.76 - -1.55) | 4490 (2906,7831) | 53.66 (29.97,102.25) | -2.11 (-2.51 - -1.71) |
|  | Male | 790 (485,1246) | 8.59 (4.39,14.81) | -0.67 (-0.84 - -0.51) | 6468 (5766,7192) | 76.53 (67.07,86.17) | -0.76 (-0.80 - -0.73) | 47 (24,78) | 0.48 (0.2,0.94) | -1.08 (-1.55 - -0.61) | 9241 (6079,13884) | 49.24 (25.41,89.04) | -1.09 (-1.48 - -0.70) |
| Albania | Both | 37 (22,63) | 5.94 (2.82,10.49) | -0.61 (-0.72 - -0.49) | 345 (306,388) | 53.19 (46.55,60.18) | -0.66 (-0.69 - -0.63) | 0 (0,0) | 0.03 (0.02,0.05) | -1.88 (-2.91 - -0.83) | 33 (23,44) | 12.32 (8.06,17.66) | -0.92 (-1.22 - -0.62) |
|  | Female | 20 (12,34) | 6.75 (3.11,12.17) | -0.70 (-0.78 - -0.62) | 186 (165,210) | 60.25 (52.42,68.67) | -0.60 (-0.62 - -0.57) | 0 (0,0) | 0.04 (0.02,0.07) | -0.78 (-2.48 - 0.95) | 46 (31,66) | 15.23 (8.57,24.21) | -0.60 (-1.08 - -0.12) |
|  | Male | 17 (10,29) | 5.2 (2.35,9.39) | -0.43 (-0.47 - -0.38) | 159 (141,181) | 46.81 (40.78,53.27) | -0.70 (-0.75 - -0.64) | 0 (0,0) | 0.02 (0.01,0.03) | -3.24 (-4.16 - -2.31) | 78 (55,104) | 9.67 (6.53,13.65) | -1.21 (-1.31 - -1.11) |
| Algeria | Both | 1423 (837,2256) | 8.81 (4.25,15.39) | -0.40 (-0.48 - -0.31) | 12177 (10794,13713) | 80.08 (70.29,90.76) | -0.54 (-0.56 - -0.51) | 25 (16,44) | 0.16 (0.09,0.29) | -4.65 (-4.87 - -4.42) | 1619 (1172,2278) | 24.07 (16.28,35.85) | -3.52 (-3.68 - -3.35) |
|  | Female | 760 (448,1201) | 9.72 (4.71,16.87) | -0.25 (-0.36 - -0.15) | 6364 (5577,7162) | 86.09 (74.95,97.51) | -0.44 (-0.45 - -0.42) | 14 (8,27) | 0.19 (0.09,0.4) | -4.32 (-4.73 - -3.91) | 2124 (1502,3250) | 28.23 (17.27,45.97) | -3.25 (-3.55 - -2.95) |
|  | Male | 662 (380,1053) | 7.93 (3.73,14.04) | -0.56 (-0.67 - -0.45) | 5812 (5127,6575) | 74.36 (64.65,85.08) | -0.64 (-0.68 - -0.60) | 11 (7,19) | 0.13 (0.07,0.25) | -5.03 (-5.23 - -4.83) | 3742 (2769,5384) | 20.11 (13.01,30.52) | -3.82 (-3.96 - -3.68) |
| American Samoa | Both | 2 (1,3) | 8.42 (4.05,14.85) | -0.22 (-0.26 - -0.17) | 19 (17,21) | 93.12 (82.28,105.38) | -0.39 (-0.40 - -0.38) | 0 (0,0) | 0.08 (0.05,0.13) | -1.40 (-2.05 - -0.73) | 2 (1,3) | 24.14 (15.43,34.7) | -0.74 (-0.99 - -0.48) |
|  | Female | 1 (1,1) | 9.68 (4.55,17.02) | -0.27 (-0.31 - -0.22) | 10 (9,12) | 105.17 (92.34,119.3) | -0.50 (-0.51 - -0.49) | 0 (0,0) | 0.11 (0.06,0.19) | -1.02 (-1.73 - -0.31) | 3 (2,4) | 29.55 (16.43,46.42) | -0.76 (-0.96 - -0.56) |
|  | Male | 1 (0,1) | 7.24 (3.35,13.05) | -0.16 (-0.22 - -0.10) | 9 (8,10) | 81.91 (71.74,93.31) | -0.29 (-0.30 - -0.28) | 0 (0,0) | 0.06 (0.03,0.11) | -1.81 (-2.49 - -1.13) | 5 (3,6) | 19.13 (11.22,29.44) | -0.73 (-0.95 - -0.51) |
| Andorra | Both | 1 (0,1) | 4.19 (1.45,8.27) | -0.34 (-0.43 - -0.26) | 7 (6,8) | 42.49 (35.85,49.71) | -0.20 (-0.22 - -0.17) | 0 (0,0) | 0.01 (0,0.01) | -7.87 (-8.66 - -7.07) | 0 (0,1) | 6.29 (4.37,8.63) | -2.80 (-3.29 - -2.31) |
|  | Female | 0 (0,1) | 4.49 (1.57,9.04) | -0.38 (-0.47 - -0.30) | 4 (3,4) | 47.14 (39.51,55.55) | -0.26 (-0.28 - -0.23) | 0 (0,0) | 0.01 (0.01,0.02) | -7.59 (-8.69 - -6.48) | 1 (0,1) | 7.7 (5.05,11.08) | -3.07 (-3.44 - -2.70) |
|  | Male | 0 (0,1) | 3.9 (1.25,7.82) | -0.25 (-0.34 - -0.16) | 3 (3,4) | 38.09 (32.04,44.44) | -0.13 (-0.15 - -0.11) | 0 (0,0) | 0.01 (0,0.01) | -8.13 (-9.43 - -6.82) | 1 (1,1) | 4.96 (3.52,6.7) | -2.57 (-3.12 - -2.02) |
| Angola | Both | 1316 (743,2237) | 7.09 (3.23,12.84) | -0.59 (-0.66 - -0.52) | 11479 (10118,12923) | 67.6 (58.77,77.04) | -0.59 (-0.61 - -0.57) | 29 (13,49) | 0.15 (0.06,0.27) | -4.77 (-5.04 - -4.50) | 1989 (1025,3243) | 20.99 (11.92,33.32) | -3.73 (-3.96 - -3.51) |
|  | Female | 756 (424,1266) | 8.03 (3.65,14.61) | -0.54 (-0.62 - -0.47) | 6781 (5992,7637) | 78.62 (68.14,89.9) | -0.53 (-0.54 - -0.51) | 12 (6,21) | 0.13 (0.06,0.25) | -4.43 (-4.61 - -4.25) | 1924 (1330,2726) | 21.27 (12.13,33.92) | -3.21 (-3.38 - -3.04) |
|  | Male | 561 (303,959) | 6.14 (2.65,11.41) | -0.65 (-0.68 - -0.61) | 4698 (4089,5336) | 56.3 (48.37,64.76) | -0.67 (-0.70 - -0.64) | 17 (6,32) | 0.17 (0.06,0.33) | -4.90 (-5.27 - -4.54) | 3913 (2519,5653) | 20.58 (9.8,36.36) | -4.14 (-4.33 - -3.94) |
| Antigua and Barbuda | Both | 1 (1,2) | 5.9 (2.47,11.14) | -0.13 (-0.18 - -0.08) | 15 (13,17) | 62.93 (54.79,71.37) | -0.16 (-0.17 - -0.16) | 0 (0,0) | 0.04 (0.03,0.05) | -1.81 (-2.79 - -0.82) | 1 (0,1) | 8.17 (6.23,10.59) | -0.84 (-1.28 - -0.40) |
|  | Female | 1 (0,1) | 7.4 (3.17,13.78) | -0.06 (-0.11 - -0.00) | 9 (8,10) | 77.84 (67.6,88.82) | -0.12 (-0.13 - -0.11) | 0 (0,0) | 0.06 (0.04,0.07) | -1.54 (-2.76 - -0.32) | 1 (1,2) | 11.6 (8.36,15.76) | -0.72 (-1.16 - -0.28) |
|  | Male | 1 (0,1) | 4.46 (1.6,8.91) | -0.23 (-0.37 - -0.09) | 6 (5,7) | 48.58 (41.55,56.28) | -0.20 (-0.22 - -0.18) | 0 (0,0) | 0.02 (0.01,0.02) | -1.82 (-2.72 - -0.91) | 2 (2,2) | 4.87 (3.68,6.28) | -0.82 (-1.15 - -0.49) |
| Argentina | Both | 591 (284,1079) | 4.32 (1.6,8.48) | -0.62 (-0.72 - -0.52) | 5964 (5144,6868) | 41.99 (35.72,48.78) | -0.40 (-0.41 - -0.38) | 3 (3,3) | 0.02 (0.02,0.03) | -5.62 (-6.55 - -4.68) | 463 (355,585) | 7.62 (5.66,10.09) | -2.60 (-2.88 - -2.31) |
|  | Female | 315 (153,583) | 4.71 (1.8,9.28) | -0.56 (-0.67 - -0.45) | 3280 (2824,3769) | 46.94 (39.96,54.42) | -0.29 (-0.31 - -0.28) | 1 (1,2) | 0.02 (0.01,0.03) | -6.15 (-6.86 - -5.42) | 611 (444,821) | 8.81 (6.2,12.24) | -2.43 (-2.64 - -2.22) |
|  | Male | 276 (127,493) | 3.95 (1.38,7.95) | -0.70 (-0.77 - -0.62) | 2685 (2298,3093) | 37.2 (31.31,43.74) | -0.51 (-0.53 - -0.49) | 2 (1,2) | 0.02 (0.02,0.03) | -5.15 (-6.15 - -4.14) | 1074 (811,1405) | 6.46 (4.88,8.31) | -2.79 (-3.21 - -2.37) |
| Armenia | Both | 44 (23,79) | 5.81 (2.34,10.93) | -0.34 (-0.38 - -0.29) | 359 (310,414) | 47.72 (40.79,55.42) | -0.13 (-0.14 - -0.12) | 0 (0,0) | 0.02 (0.01,0.03) | -4.25 (-6.23 - -2.22) | 33 (24,44) | 10.3 (7.05,14.56) | -1.06 (-2.06 - -0.06) |
|  | Female | 25 (13,46) | 7.09 (3,13.32) | -0.09 (-0.14 - -0.04) | 208 (181,239) | 59 (50.41,68.51) | 0.01 (-0.01 - 0.02) | 0 (0,0) | 0.01 (0.01,0.02) | -5.13 (-6.95 - -3.29) | 44 (29,63) | 12.6 (7.13,19.64) | -1.09 (-1.46 - -0.71) |
|  | Male | 19 (9,33) | 4.68 (1.64,9.11) | -0.63 (-0.70 - -0.56) | 151 (128,174) | 37.76 (31.76,44.16) | -0.30 (-0.31 - -0.28) | 0 (0,0) | 0.02 (0.02,0.03) | -2.91 (-4.64 - -1.15) | 77 (55,102) | 8.28 (5.92,11.59) | -1.20 (-1.90 - -0.49) |
| Australia | Both | 240 (144,354) | 3.86 (1.77,6.7) | -0.15 (-0.20 - -0.11) | 2373 (2094,2663) | 37.79 (32.92,42.82) | -0.24 (-0.27 - -0.22) | 0 (0,0) | 0.01 (0,0.01) | -6.73 (-8.01 - -5.43) | 178 (128,234) | 5.69 (4.04,7.43) | -1.81 (-2.22 - -1.40) |
|  | Female | 100 (52,162) | 3.32 (1.28,6.29) | -0.41 (-0.53 - -0.28) | 1075 (928,1228) | 35.23 (29.93,40.78) | -0.34 (-0.36 - -0.31) | 0 (0,0) | 0.01 (0,0.01) | -6.54 (-7.83 - -5.24) | 178 (127,232) | 5.84 (4.13,7.71) | -1.46 (-1.86 - -1.05) |
|  | Male | 140 (88,203) | 4.37 (2.05,7.43) | 0.02 (-0.04 - 0.08) | 1298 (1147,1457) | 40.21 (35.11,45.47) | -0.19 (-0.21 - -0.17) | 0 (0,0) | 0.01 (0,0.01) | -6.91 (-8.05 - -5.75) | 356 (257,464) | 5.54 (3.97,7.32) | -2.13 (-2.34 - -1.92) |
| Austria | Both | 93 (43,167) | 5.28 (2.05,10.33) | -0.60 (-0.69 - -0.52) | 979 (841,1121) | 54.73 (46.29,63.59) | -0.35 (-0.36 - -0.34) | 0 (0,0) | 0.01 (0,0.01) | -8.05 (-8.70 - -7.40) | 59 (42,80) | 7.56 (5.12,10.8) | -2.11 (-2.33 - -1.90) |
|  | Female | 48 (23,86) | 5.63 (2.17,10.92) | -0.40 (-0.47 - -0.32) | 517 (445,596) | 59.99 (50.55,69.98) | -0.20 (-0.22 - -0.18) | 0 (0,0) | 0.01 (0,0.01) | -6.92 (-8.06 - -5.76) | 76 (52,109) | 8.8 (5.62,13.15) | -1.42 (-1.71 - -1.13) |
|  | Male | 45 (20,81) | 4.95 (1.82,9.91) | -0.82 (-0.91 - -0.72) | 461 (394,529) | 49.83 (41.56,58.2) | -0.51 (-0.53 - -0.49) | 0 (0,0) | 0.01 (0,0.01) | -8.87 (-9.46 - -8.28) | 135 (97,186) | 6.41 (4.15,9.22) | -2.81 (-2.96 - -2.66) |
| Azerbaijan | Both | 165 (84,286) | 5.46 (2.12,10.34) | -0.41 (-0.49 - -0.34) | 1337 (1155,1524) | 44.23 (37.71,51.21) | -0.25 (-0.28 - -0.23) | 1 (0,1) | 0.02 (0.01,0.03) | -2.77 (-3.85 - -1.66) | 113 (81,155) | 9.8 (6.47,13.82) | -0.93 (-1.41 - -0.46) |
|  | Female | 100 (53,177) | 7.08 (2.93,13.5) | -0.25 (-0.34 - -0.16) | 814 (699,925) | 57.73 (49.1,66.89) | -0.10 (-0.11 - -0.08) | 0 (0,1) | 0.02 (0.01,0.04) | -3.14 (-4.07 - -2.21) | 182 (119,248) | 12.95 (7.56,19.63) | -0.67 (-0.83 - -0.51) |
|  | Male | 65 (29,119) | 4.04 (1.29,8.17) | -0.56 (-0.72 - -0.39) | 523 (446,604) | 32.42 (27.23,38.04) | -0.42 (-0.43 - -0.41) | 0 (0,0) | 0.02 (0.01,0.03) | -2.74 (-4.26 - -1.19) | 295 (209,399) | 7.06 (4.81,9.93) | -1.16 (-2.09 - -0.21) |
| Bahamas | Both | 7 (4,12) | 6.23 (2.64,11.49) | -0.05 (-0.14 - 0.04) | 84 (74,95) | 66.49 (57.49,75.65) | -0.08 (-0.10 - -0.06) | 0 (0,0) | 0.02 (0.02,0.03) | -3.33 (-4.96 - -1.67) | 3 (2,4) | 7.32 (5.25,9.97) | -1.22 (-1.85 - -0.59) |
|  | Female | 4 (2,7) | 7.76 (3.33,14.24) | -0.01 (-0.06 - 0.05) | 52 (45,59) | 81.9 (70.94,93.27) | -0.05 (-0.07 - -0.03) | 0 (0,0) | 0.03 (0.02,0.04) | -3.22 (-5.90 - -0.46) | 6 (4,8) | 9.72 (6.67,13.87) | -1.19 (-2.03 - -0.34) |
|  | Male | 3 (1,5) | 4.7 (1.73,9.15) | -0.17 (-0.29 - -0.06) | 32 (28,37) | 51.02 (43.85,59) | -0.14 (-0.16 - -0.11) | 0 (0,0) | 0.01 (0.01,0.02) | -3.47 (-4.30 - -2.64) | 9 (7,12) | 4.91 (3.6,6.37) | -1.35 (-1.65 - -1.05) |
| Bahrain | Both | 34 (20,57) | 8.51 (3.88,15.29) | -0.95 (-1.12 - -0.78) | 339 (297,380) | 81.56 (70.76,92.75) | -0.90 (-0.93 - -0.87) | 0 (0,1) | 0.11 (0.08,0.15) | -3.61 (-4.04 - -3.18) | 40 (31,50) | 20.07 (14.94,26.42) | -2.45 (-2.67 - -2.23) |
|  | Female | 18 (11,31) | 9.72 (4.54,17.24) | -0.83 (-1.01 - -0.65) | 174 (152,196) | 90.69 (78.46,102.83) | -0.68 (-0.72 - -0.64) | 0 (0,0) | 0.11 (0.07,0.17) | -3.76 (-4.64 - -2.87) | 43 (31,56) | 22.5 (15.32,31.43) | -2.34 (-2.73 - -1.95) |
|  | Male | 16 (9,27) | 7.46 (3.19,13.73) | -1.01 (-1.18 - -0.85) | 165 (143,187) | 73.85 (63.5,84.61) | -1.10 (-1.13 - -1.08) | 0 (0,0) | 0.11 (0.07,0.17) | -3.29 (-4.09 - -2.49) | 83 (64,104) | 18.03 (12.76,24.32) | -2.42 (-2.89 - -1.96) |
| Bangladesh | Both | 2608 (1228,4535) | 4.25 (1.55,8.38) | -0.03 (-0.11 - 0.04) | 23319 (19959,26632) | 37.4 (31.55,43.4) | -0.22 (-0.27 - -0.16) | 72 (50,108) | 0.12 (0.07,0.2) | -3.81 (-3.98 - -3.65) | 4058 (2818,6182) | 15.08 (10.49,21.56) | -3.09 (-3.26 - -2.92) |
|  | Female | 1537 (731,2757) | 5.02 (1.9,9.67) | 0.11 (-0.05 - 0.28) | 14347 (12229,16540) | 45.63 (38.36,53) | -0.05 (-0.10 - 0.00) | 37 (22,62) | 0.12 (0.06,0.24) | -3.80 (-3.95 - -3.65) | 5122 (3663,7424) | 16.72 (10.73,26.67) | -2.90 (-3.09 - -2.71) |
|  | Male | 1071 (439,1962) | 3.49 (1.06,7.19) | -0.23 (-0.32 - -0.15) | 8972 (7596,10313) | 29.03 (24.1,34.28) | -0.40 (-0.42 - -0.39) | 36 (21,61) | 0.12 (0.06,0.23) | -3.85 (-4.14 - -3.55) | 9181 (6987,12326) | 13.37 (8.03,22.34) | -3.34 (-3.59 - -3.09) |
| Barbados | Both | 4 (2,7) | 6.35 (2.63,11.63) | 0.05 (-0.05 - 0.15) | 48 (43,54) | 68.12 (59.48,77.27) | -0.02 (-0.03 - -0.01) | 0 (0,0) | 0.03 (0.02,0.04) | -3.19 (-3.58 - -2.80) | 2 (1,2) | 8.1 (5.92,10.76) | -1.29 (-1.44 - -1.14) |
|  | Female | 2 (1,4) | 7.94 (3.37,14.35) | 0.12 (0.05 - 0.19) | 29 (26,33) | 83.68 (72.75,95.21) | -0.00 (-0.01 - 0.01) | 0 (0,0) | 0.04 (0.03,0.06) | -2.91 (-3.28 - -2.54) | 4 (3,5) | 11.14 (7.65,15.57) | -1.55 (-2.00 - -1.09) |
|  | Male | 2 (1,3) | 4.83 (1.75,9.41) | -0.04 (-0.16 - 0.08) | 19 (17,22) | 53.29 (45.83,61.33) | -0.02 (-0.03 - -0.00) | 0 (0,0) | 0.02 (0.01,0.03) | -2.96 (-7.14 - 1.40) | 6 (4,7) | 5.2 (3.88,6.84) | -1.06 (-1.57 - -0.54) |
| Belarus | Both | 130 (67,231) | 6.46 (2.73,12.03) | -0.52 (-0.60 - -0.45) | 1111 (968,1267) | 55.38 (47.28,63.65) | -0.25 (-0.26 - -0.24) | 0 (0,0) | 0.02 (0.01,0.02) | -6.20 (-7.29 - -5.09) | 76 (54,102) | 11.56 (7.22,17.21) | -1.67 (-2.06 - -1.27) |
|  | Female | 84 (45,145) | 8.6 (3.89,16.08) | -0.45 (-0.54 - -0.37) | 715 (620,814) | 73.5 (62.7,84.42) | -0.27 (-0.30 - -0.25) | 0 (0,0) | 0.02 (0.02,0.03) | -5.52 (-7.35 - -3.65) | 155 (99,225) | 15.99 (9.09,25.31) | -1.40 (-1.75 - -1.04) |
|  | Male | 46 (20,83) | 4.44 (1.45,8.87) | -0.66 (-0.80 - -0.51) | 396 (342,457) | 38.32 (32.21,44.97) | -0.18 (-0.21 - -0.15) | 0 (0,0) | 0.01 (0.01,0.01) | -6.87 (-8.20 - -5.52) | 231 (155,314) | 7.39 (5.01,10.32) | -2.06 (-2.94 - -1.17) |
| Belgium | Both | 103 (47,182) | 4.07 (1.4,8.19) | -0.83 (-0.90 - -0.76) | 1100 (935,1260) | 42.25 (35.32,49.18) | -0.51 (-0.54 - -0.48) | 0 (0,0) | 0.01 (0.01,0.01) | -8.39 (-9.55 - -7.23) | 67 (49,88) | 6.16 (4.34,8.49) | -2.83 (-3.01 - -2.65) |
|  | Female | 55 (25,96) | 4.43 (1.59,9.03) | -0.83 (-0.91 - -0.76) | 603 (513,700) | 47.44 (39.78,55.45) | -0.48 (-0.52 - -0.44) | 0 (0,0) | 0.01 (0.01,0.01) | -7.71 (-8.27 - -7.14) | 93 (66,129) | 7.31 (4.93,10.6) | -2.34 (-2.77 - -1.91) |
|  | Male | 48 (22,86) | 3.73 (1.13,7.65) | -0.84 (-0.99 - -0.70) | 497 (416,576) | 37.3 (30.87,44) | -0.54 (-0.56 - -0.51) | 0 (0,0) | 0.01 (0.01,0.01) | -8.54 (-9.21 - -7.88) | 160 (117,213) | 5.06 (3.62,6.67) | -3.36 (-3.60 - -3.12) |
| Belize | Both | 9 (4,16) | 5.51 (2.2,10.39) | 0.08 (-0.00 - 0.17) | 99 (87,112) | 56.26 (48.7,63.99) | -0.03 (-0.04 - -0.02) | 0 (0,0) | 0.03 (0.02,0.03) | -4.21 (-4.54 - -3.88) | 4 (3,5) | 6.78 (5.11,8.87) | -2.11 (-2.37 - -1.86) |
|  | Female | 6 (3,10) | 6.83 (2.7,12.68) | 0.15 (0.09 - 0.20) | 61 (53,69) | 68.93 (59.6,78.57) | 0.02 (0.01 - 0.02) | 0 (0,0) | 0.03 (0.02,0.04) | -3.90 (-4.75 - -3.04) | 8 (6,10) | 8.7 (6.23,12) | -1.73 (-2.08 - -1.38) |
|  | Male | 4 (2,6) | 4.21 (1.45,8.49) | -0.07 (-0.26 - 0.12) | 39 (33,44) | 43.64 (36.91,50.39) | -0.11 (-0.12 - -0.09) | 0 (0,0) | 0.02 (0.01,0.03) | -4.74 (-5.54 - -3.94) | 12 (9,15) | 4.87 (3.63,6.23) | -2.66 (-3.11 - -2.20) |
| Benin | Both | 746 (464,1156) | 9.74 (5.1,16.34) | -0.37 (-0.44 - -0.31) | 6710 (6033,7400) | 97.3 (86.7,108.06) | -0.36 (-0.37 - -0.34) | 22 (11,37) | 0.26 (0.12,0.48) | -1.35 (-1.80 - -0.90) | 1578 (795,2717) | 34.58 (19.62,56.24) | -1.01 (-1.34 - -0.68) |
|  | Female | 454 (294,681) | 11.5 (6.2,18.9) | -0.24 (-0.33 - -0.16) | 4523 (4080,4991) | 129.25 (115.33,143.59) | -0.27 (-0.29 - -0.25) | 6 (4,11) | 0.17 (0.08,0.33) | -0.76 (-1.08 - -0.44) | 1152 (843,1592) | 31.08 (18.28,50.07) | -0.51 (-0.66 - -0.35) |
|  | Male | 292 (168,495) | 8.08 (3.77,14.5) | -0.58 (-0.64 - -0.51) | 2187 (1918,2474) | 65.54 (56.61,75.05) | -0.56 (-0.59 - -0.54) | 15 (6,29) | 0.35 (0.13,0.7) | -1.59 (-2.08 - -1.09) | 2730 (1808,4149) | 37.76 (17.34,68.59) | -1.47 (-1.71 - -1.22) |
| Bermuda | Both | 1 (0,1) | 5.84 (2.43,11) | -0.12 (-0.29 - 0.05) | 8 (7,8) | 62.65 (54.28,71.37) | -0.13 (-0.14 - -0.12) | 0 (0,0) | 0.01 (0.01,0.01) | -4.95 (-6.30 - -3.57) | 0 (0,0) | 5.84 (4.12,8.01) | -1.02 (-1.31 - -0.72) |
|  | Female | 0 (0,1) | 7.3 (3.15,13.47) | -0.03 (-0.08 - 0.03) | 5 (4,5) | 76.74 (66.79,87.41) | -0.09 (-0.10 - -0.08) | 0 (0,0) | 0.01 (0,0.01) | -5.37 (-6.42 - -4.32) | 0 (0,1) | 7.6 (5.05,10.85) | -0.99 (-1.19 - -0.80) |
|  | Male | 0 (0,0) | 4.43 (1.59,8.66) | -0.24 (-0.34 - -0.13) | 3 (3,3) | 48.95 (41.83,56.47) | -0.17 (-0.20 - -0.15) | 0 (0,0) | 0.01 (0,0.01) | -3.80 (-5.09 - -2.49) | 1 (1,1) | 4.14 (2.99,5.45) | -1.01 (-1.35 - -0.67) |
| Bhutan | Both | 11 (5,21) | 4.38 (1.58,8.71) | -0.08 (-0.25 - 0.08) | 101 (87,115) | 38.19 (32.32,44.36) | -0.23 (-0.24 - -0.22) | 0 (0,0) | 0.07 (0.04,0.11) | -4.24 (-4.75 - -3.71) | 14 (10,18) | 11.12 (7.66,15.29) | -2.97 (-3.35 - -2.59) |
|  | Female | 7 (3,12) | 5.15 (1.89,10.19) | -0.03 (-0.21 - 0.15) | 59 (50,68) | 44.9 (37.76,52.28) | -0.18 (-0.21 - -0.16) | 0 (0,0) | 0.06 (0.02,0.11) | -4.71 (-5.36 - -4.06) | 15 (11,20) | 11.5 (7.45,17.52) | -2.89 (-3.42 - -2.35) |
|  | Male | 5 (2,8) | 3.61 (1.13,7.38) | -0.24 (-0.40 - -0.08) | 42 (36,49) | 31.56 (26.29,37.02) | -0.37 (-0.39 - -0.36) | 0 (0,0) | 0.08 (0.04,0.14) | -3.64 (-4.37 - -2.90) | 29 (21,36) | 10.73 (6.76,15.85) | -2.88 (-3.44 - -2.32) |
| Bolivia (Plurinational State of) | Both | 218 (113,382) | 4.82 (1.98,9.1) | -0.80 (-0.91 - -0.69) | 1909 (1656,2171) | 42.71 (36.55,49.27) | -0.74 (-0.77 - -0.72) | 7 (5,10) | 0.16 (0.1,0.23) | -4.82 (-4.93 - -4.71) | 399 (271,571) | 16.67 (11.53,23.19) | -4.37 (-4.47 - -4.26) |
|  | Female | 122 (66,213) | 5.46 (2.21,10.48) | -0.80 (-0.87 - -0.73) | 1066 (922,1218) | 48.43 (41.24,56.06) | -0.70 (-0.72 - -0.68) | 3 (2,5) | 0.14 (0.08,0.24) | -4.79 (-4.97 - -4.62) | 360 (269,487) | 16.17 (10.53,24.26) | -4.20 (-4.29 - -4.10) |
|  | Male | 97 (49,159) | 4.19 (1.57,7.91) | -0.78 (-0.95 - -0.61) | 842 (731,964) | 37.15 (31.79,42.95) | -0.79 (-0.82 - -0.76) | 4 (2,6) | 0.17 (0.09,0.27) | -4.82 (-5.12 - -4.53) | 759 (580,983) | 17.12 (10.52,26.05) | -4.51 (-4.78 - -4.23) |
| Bosnia and Herzegovina | Both | 59 (37,94) | 8.66 (4.53,14.88) | 0.44 (0.35 - 0.52) | 443 (391,498) | 63.79 (55.43,72.29) | 0.06 (0.04 - 0.09) | 0 (0,0) | 0.04 (0.02,0.06) | -2.05 (-4.68 - 0.65) | 47 (31,64) | 14.73 (10.01,20.54) | -0.47 (-1.13 - 0.19) |
|  | Female | 28 (17,47) | 8.65 (4.01,15.59) | 0.13 (0.05 - 0.21) | 246 (217,279) | 73.14 (63.4,83.56) | -0.08 (-0.11 - -0.05) | 0 (0,0) | 0.03 (0.02,0.05) | -2.63 (-4.37 - -0.87) | 55 (37,81) | 16.44 (9.8,25.27) | -0.37 (-0.64 - -0.09) |
|  | Male | 31 (19,46) | 8.68 (4.73,14.47) | 0.78 (0.66 - 0.90) | 197 (172,221) | 54.96 (47.38,62.75) | 0.26 (0.22 - 0.30) | 0 (0,0) | 0.05 (0.03,0.09) | -1.68 (-4.47 - 1.20) | 102 (74,136) | 13.13 (8.1,19.45) | -0.50 (-1.43 - 0.42) |
| Botswana | Both | 68 (38,114) | 7.38 (3.38,13.24) | 0.00 (-0.06 - 0.07) | 654 (574,734) | 71.71 (62.59,81.61) | -0.02 (-0.03 - -0.02) | 1 (1,2) | 0.12 (0.06,0.21) | 0.37 (-0.33 - 1.08) | 79 (53,115) | 19.15 (11.79,29.77) | 0.26 (0.00 - 0.51) |
|  | Female | 42 (24,68) | 9.18 (4.16,16.11) | -0.06 (-0.15 - 0.04) | 415 (364,468) | 92.06 (80.08,104.97) | -0.09 (-0.10 - -0.08) | 0 (0,1) | 0.1 (0.05,0.2) | 0.11 (-0.62 - 0.85) | 96 (69,128) | 21.17 (12.23,34.19) | 0.02 (-0.23 - 0.27) |
|  | Male | 26 (14,45) | 5.63 (2.32,10.5) | 0.16 (0.12 - 0.20) | 238 (207,270) | 51.79 (44.32,59.45) | 0.19 (0.18 - 0.19) | 1 (0,1) | 0.13 (0.05,0.26) | 0.73 (0.06 - 1.40) | 175 (130,232) | 17.16 (9.83,28.29) | 0.58 (0.14 - 1.02) |
| Brazil | Both | 2492 (1192,4363) | 3.9 (1.45,7.63) | -1.17 (-1.32 - -1.02) | 23594 (18890,29017) | 37 (28.86,46.21) | -0.93 (-0.95 - -0.91) | 23 (21,26) | 0.04 (0.03,0.04) | -3.83 (-4.69 - -2.96) | 1679 (1432,1989) | 5.91 (4.79,7.26) | -2.72 (-3.25 - -2.18) |
|  | Female | 1377 (650,2438) | 4.39 (1.62,8.52) | -1.07 (-1.38 - -0.76) | 13529 (10854,16612) | 43.22 (33.85,54.22) | -0.78 (-0.81 - -0.76) | 11 (10,12) | 0.04 (0.03,0.04) | -3.60 (-4.31 - -2.89) | 2101 (1685,2585) | 6.69 (5.27,8.35) | -2.35 (-2.64 - -2.04) |
|  | Male | 1115 (534,1982) | 3.42 (1.22,6.86) | -1.16 (-1.21 - -1.11) | 10065 (7990,12423) | 31 (24.07,38.71) | -1.10 (-1.13 - -1.07) | 12 (11,14) | 0.04 (0.03,0.04) | -4.26 (-5.12 - -3.39) | 3780 (3137,4577) | 5.15 (4.22,6.26) | -3.28 (-4.02 - -2.53) |
| Brunei Darussalam | Both | 6 (3,10) | 4.33 (1.59,8.51) | -0.77 (-0.84 - -0.70) | 59 (51,68) | 44.58 (37.81,51.74) | -0.53 (-0.57 - -0.49) | 0 (0,0) | 0.02 (0.01,0.03) | -5.55 (-7.11 - -3.96) | 5 (4,6) | 8.85 (6.14,12.02) | -2.03 (-2.35 - -1.72) |
|  | Female | 3 (1,5) | 4.95 (1.87,9.54) | -0.62 (-0.65 - -0.59) | 33 (28,38) | 52.68 (44.89,61.51) | -0.44 (-0.47 - -0.41) | 0 (0,0) | 0.02 (0.01,0.03) | -4.35 (-6.17 - -2.50) | 7 (5,9) | 10.87 (7.2,15.68) | -1.34 (-1.78 - -0.90) |
|  | Male | 3 (1,5) | 3.78 (1.16,7.87) | -0.93 (-1.07 - -0.80) | 26 (22,30) | 37.33 (31.36,43.47) | -0.64 (-0.64 - -0.63) | 0 (0,0) | 0.02 (0.01,0.03) | -6.73 (-8.65 - -4.77) | 12 (9,15) | 7.04 (5,9.42) | -2.88 (-3.55 - -2.21) |
| Bulgaria | Both | 72 (39,125) | 5.53 (2.33,10.34) | -0.14 (-0.16 - -0.12) | 694 (576,821) | 52.45 (42.85,62.63) | -0.24 (-0.26 - -0.21) | 1 (1,1) | 0.07 (0.06,0.08) | -3.37 (-5.81 - -0.88) | 86 (68,107) | 14.87 (10.83,19.7) | -1.72 (-2.33 - -1.09) |
|  | Female | 43 (24,73) | 6.89 (2.98,12.67) | -0.05 (-0.07 - -0.03) | 420 (350,492) | 65.5 (53.74,77.58) | -0.21 (-0.22 - -0.20) | 0 (0,0) | 0.06 (0.05,0.07) | -3.61 (-4.82 - -2.38) | 109 (78,147) | 17.09 (11.31,24.87) | -1.68 (-2.38 - -0.98) |
|  | Male | 28 (14,50) | 4.25 (1.58,8.34) | -0.26 (-0.30 - -0.23) | 274 (222,332) | 40.16 (31.94,49.45) | -0.27 (-0.31 - -0.22) | 1 (0,1) | 0.08 (0.06,0.1) | -3.19 (-5.63 - -0.69) | 195 (149,248) | 12.77 (9.64,16.42) | -1.99 (-3.57 - -0.38) |
| Burkina Faso | Both | 1079 (655,1758) | 8.25 (4.11,14.19) | -0.47 (-0.59 - -0.35) | 9255 (8289,10248) | 79.49 (70.19,89.13) | -0.41 (-0.44 - -0.39) | 28 (12,52) | 0.19 (0.08,0.38) | -1.14 (-1.38 - -0.89) | 2186 (981,4082) | 26.53 (13.96,45.2) | -0.91 (-1.07 - -0.74) |
|  | Female | 661 (414,1052) | 9.69 (4.96,16.65) | -0.30 (-0.41 - -0.19) | 6235 (5633,6837) | 104.32 (93.02,116.31) | -0.29 (-0.32 - -0.27) | 7 (4,13) | 0.1 (0.04,0.21) | -0.40 (-0.67 - -0.12) | 1421 (1023,2037) | 22.4 (12.29,38.53) | -0.36 (-0.52 - -0.21) |
|  | Male | 419 (233,707) | 6.87 (3.08,12.41) | -0.69 (-0.84 - -0.55) | 3020 (2635,3442) | 54.22 (46.7,62.63) | -0.67 (-0.70 - -0.65) | 21 (7,43) | 0.28 (0.09,0.59) | -1.35 (-1.60 - -1.11) | 3607 (2185,5668) | 30.39 (12.69,58.08) | -1.24 (-1.45 - -1.03) |
| Burundi | Both | 481 (270,811) | 6.68 (2.99,12.3) | -0.57 (-0.65 - -0.48) | 4043 (3547,4576) | 60.26 (52.19,68.78) | -0.56 (-0.57 - -0.54) | 9 (6,14) | 0.13 (0.07,0.21) | -3.54 (-3.77 - -3.30) | 446 (328,623) | 18.16 (12.32,26.01) | -2.64 (-2.83 - -2.45) |
|  | Female | 272 (153,452) | 7.41 (3.33,13.62) | -0.47 (-0.57 - -0.37) | 2396 (2102,2727) | 69.9 (60.49,80.01) | -0.50 (-0.51 - -0.49) | 6 (4,10) | 0.17 (0.08,0.32) | -3.34 (-3.56 - -3.11) | 827 (593,1120) | 23.42 (14.02,36.37) | -2.54 (-2.75 - -2.33) |
|  | Male | 209 (110,367) | 5.94 (2.44,11.29) | -0.68 (-0.75 - -0.60) | 1647 (1430,1886) | 50.3 (42.7,58.17) | -0.65 (-0.67 - -0.63) | 3 (2,5) | 0.08 (0.04,0.15) | -3.97 (-4.20 - -3.75) | 1272 (982,1661) | 12.72 (8.43,19.36) | -2.87 (-3.05 - -2.68) |
| Cabo Verde | Both | 19 (12,31) | 9.99 (4.9,17.57) | -0.19 (-0.22 - -0.15) | 192 (172,213) | 96.77 (85.42,108.59) | -0.42 (-0.43 - -0.40) | 0 (0,0) | 0.16 (0.09,0.24) | -3.07 (-3.83 - -2.31) | 27 (19,35) | 25.05 (16.85,34.58) | -2.18 (-2.62 - -1.73) |
|  | Female | 10 (6,17) | 10.9 (5.3,19.05) | -0.34 (-0.42 - -0.27) | 120 (107,133) | 122.36 (108.22,137.23) | -0.53 (-0.55 - -0.52) | 0 (0,0) | 0.08 (0.04,0.14) | -3.29 (-3.71 - -2.87) | 22 (15,30) | 22.74 (13.03,35.85) | -1.66 (-1.93 - -1.40) |
|  | Male | 9 (5,15) | 9.13 (4.27,16.33) | -0.02 (-0.08 - 0.04) | 72 (63,82) | 71.74 (61.6,82.75) | -0.19 (-0.21 - -0.17) | 0 (0,0) | 0.24 (0.12,0.41) | -2.98 (-3.86 - -2.09) | 49 (38,62) | 27.31 (16.76,41.43) | -2.60 (-3.16 - -2.03) |
| Cambodia | Both | 327 (165,555) | 4.96 (1.97,9.32) | -0.25 (-0.39 - -0.11) | 2906 (2502,3324) | 44.78 (38.04,51.7) | -0.36 (-0.38 - -0.34) | 5 (3,6) | 0.07 (0.04,0.11) | -3.31 (-3.48 - -3.13) | 381 (275,502) | 14.33 (9.69,19.92) | -2.00 (-2.11 - -1.88) |
|  | Female | 192 (100,327) | 5.95 (2.43,11.1) | -0.14 (-0.18 - -0.10) | 1693 (1465,1938) | 53.52 (45.39,62.09) | -0.28 (-0.30 - -0.26) | 3 (2,4) | 0.08 (0.04,0.14) | -3.64 (-3.89 - -3.39) | 555 (408,737) | 17.43 (10.5,25.8) | -2.11 (-2.23 - -1.98) |
|  | Male | 136 (65,236) | 4.02 (1.43,7.75) | -0.38 (-0.49 - -0.27) | 1214 (1036,1399) | 36.47 (30.81,42.54) | -0.40 (-0.41 - -0.39) | 2 (1,3) | 0.06 (0.03,0.11) | -2.80 (-2.96 - -2.64) | 936 (722,1208) | 11.37 (7.38,16.54) | -1.77 (-1.86 - -1.67) |
| Cameroon | Both | 1591 (992,2526) | 9.41 (4.96,16.24) | 0.16 (0.11 - 0.22) | 14506 (13037,15983) | 91.03 (80.65,101.18) | -0.11 (-0.14 - -0.08) | 59 (33,91) | 0.33 (0.17,0.55) | 0.02 (-0.18 - 0.21) | 4366 (2412,6531) | 39.78 (23.1,59.65) | -0.06 (-0.22 - 0.11) |
|  | Female | 907 (572,1432) | 10.57 (5.61,17.95) | 0.17 (0.10 - 0.24) | 9483 (8523,10389) | 119.06 (105.5,131.83) | -0.06 (-0.09 - -0.04) | 15 (9,24) | 0.18 (0.09,0.32) | 0.68 (0.49 - 0.87) | 2523 (1882,3344) | 30.83 (17.51,47.55) | 0.23 (0.02 - 0.45) |
|  | Male | 684 (409,1142) | 8.33 (4.02,14.86) | 0.16 (0.05 - 0.26) | 5022 (4420,5672) | 63.79 (55.08,73.02) | -0.19 (-0.23 - -0.14) | 44 (21,70) | 0.48 (0.21,0.84) | -0.20 (-0.40 - 0.01) | 6889 (4668,9554) | 48.3 (24.68,79.42) | -0.23 (-0.42 - -0.05) |
| Canada | Both | 574 (305,948) | 7.09 (3.11,12.8) | -0.47 (-0.54 - -0.40) | 7018 (6149,7958) | 83.38 (72.54,94.69) | -0.26 (-0.30 - -0.22) | 3 (2,3) | 0.03 (0.03,0.04) | -4.26 (-5.18 - -3.34) | 566 (431,750) | 14.27 (10.09,19.85) | -1.66 (-1.83 - -1.48) |
|  | Female | 275 (148,455) | 7 (3.13,12.6) | -0.63 (-0.70 - -0.55) | 3409 (2987,3893) | 83.38 (72.28,95.5) | -0.31 (-0.32 - -0.30) | 1 (1,1) | 0.03 (0.02,0.04) | -4.50 (-5.59 - -3.40) | 615 (449,859) | 15.25 (9.74,23.18) | -1.56 (-1.73 - -1.40) |
|  | Male | 298 (157,501) | 7.18 (3.1,13.09) | -0.33 (-0.40 - -0.26) | 3609 (3162,4112) | 83.38 (72.14,95.21) | -0.22 (-0.26 - -0.19) | 1 (1,2) | 0.04 (0.03,0.05) | -4.15 (-5.26 - -3.02) | 1181 (888,1557) | 13.34 (9.29,18.74) | -1.73 (-1.97 - -1.49) |
| Central African Republic | Both | 212 (123,355) | 7.32 (3.39,13.07) | -0.34 (-0.49 - -0.19) | 1827 (1617,2060) | 66.83 (58.46,76.06) | -0.35 (-0.38 - -0.32) | 8 (4,13) | 0.25 (0.11,0.46) | -2.37 (-2.76 - -1.99) | 463 (253,792) | 29.16 (16.24,48.95) | -1.96 (-2.27 - -1.65) |
|  | Female | 119 (69,199) | 8.24 (3.83,14.78) | -0.34 (-0.49 - -0.18) | 1056 (939,1187) | 77.23 (67.1,88.15) | -0.36 (-0.40 - -0.32) | 3 (1,6) | 0.21 (0.08,0.45) | -2.81 (-3.26 - -2.35) | 409 (239,653) | 28.1 (14.31,50.07) | -2.18 (-2.49 - -1.87) |
|  | Male | 93 (52,155) | 6.42 (2.87,11.59) | -0.32 (-0.47 - -0.17) | 771 (675,876) | 56.47 (48.85,64.91) | -0.35 (-0.36 - -0.33) | 4 (2,8) | 0.28 (0.11,0.57) | -2.02 (-2.45 - -1.59) | 871 (530,1363) | 30.1 (14.53,55.32) | -1.72 (-2.10 - -1.35) |
| Chad | Both | 1159 (755,1770) | 10.38 (5.64,17.19) | -0.34 (-0.41 - -0.27) | 9691 (8741,10627) | 99.53 (88.62,110.28) | -0.33 (-0.35 - -0.32) | 71 (42,114) | 0.55 (0.29,0.93) | -0.22 (-0.48 - 0.04) | 5316 (3102,8566) | 59.74 (35.39,93.55) | -0.27 (-0.48 - -0.05) |
|  | Female | 681 (452,1010) | 11.6 (6.27,19.13) | -0.29 (-0.42 - -0.17) | 6351 (5746,6957) | 127.87 (114.4,141.76) | -0.25 (-0.25 - -0.24) | 14 (6,22) | 0.23 (0.1,0.43) | 0.34 (0.00 - 0.68) | 2018 (1298,2824) | 36.58 (20.1,58.17) | 0.02 (-0.16 - 0.20) |
|  | Male | 478 (301,777) | 9.25 (4.71,15.76) | -0.41 (-0.45 - -0.36) | 3340 (2953,3747) | 71.4 (62.04,81.17) | -0.45 (-0.47 - -0.43) | 57 (32,95) | 0.86 (0.43,1.52) | -0.35 (-0.60 - -0.10) | 7334 (4746,10920) | 82.01 (43.78,139.54) | -0.39 (-0.64 - -0.15) |
| Chile | Both | 238 (113,434) | 4.87 (1.83,9.57) | -0.26 (-0.43 - -0.10) | 2355 (2022,2675) | 46.6 (39.5,53.94) | -0.14 (-0.16 - -0.13) | 1 (1,1) | 0.02 (0.02,0.03) | -5.13 (-5.54 - -4.71) | 186 (143,232) | 8.46 (6.18,11.21) | -2.42 (-2.56 - -2.28) |
|  | Female | 127 (58,235) | 5.34 (2.03,10.5) | -0.05 (-0.29 - 0.18) | 1287 (1102,1475) | 52.26 (44.09,60.83) | 0.11 (0.07 - 0.14) | 1 (0,1) | 0.02 (0.02,0.03) | -5.53 (-5.95 - -5.10) | 238 (171,326) | 9.73 (6.57,14.05) | -2.28 (-2.56 - -2.01) |
|  | Male | 111 (50,200) | 4.41 (1.57,8.93) | -0.48 (-0.55 - -0.41) | 1068 (912,1220) | 41.22 (34.75,47.77) | -0.42 (-0.45 - -0.39) | 1 (1,1) | 0.03 (0.02,0.04) | -4.83 (-5.41 - -4.26) | 424 (318,545) | 7.25 (5.49,9.3) | -2.68 (-2.92 - -2.45) |
| China | Both | 22028 (11629,38662) | 6.62 (2.83,12.38) | -0.42 (-0.55 - -0.29) | 203389 (164399,249471) | 61.26 (48.45,76.56) | -0.69 (-0.72 - -0.67) | 165 (126,202) | 0.05 (0.04,0.07) | -4.09 (-4.34 - -3.83) | 23434 (17761,29940) | 15.75 (11.49,20.52) | -2.07 (-2.24 - -1.90) |
|  | Female | 12391 (6606,21766) | 7.98 (3.46,14.97) | -0.44 (-0.70 - -0.18) | 116080 (94096,142200) | 75.09 (59.27,93.47) | -0.64 (-0.65 - -0.62) | 60 (46,78) | 0.04 (0.03,0.05) | -4.85 (-5.18 - -4.53) | 28394 (20391,36994) | 18.49 (12.92,24.83) | -1.97 (-2.10 - -1.83) |
|  | Male | 9636 (4792,17100) | 5.42 (2.23,10.32) | -0.38 (-0.43 - -0.33) | 87309 (70548,107402) | 49.23 (38.65,61.39) | -0.71 (-0.75 - -0.68) | 105 (79,133) | 0.06 (0.04,0.08) | -3.67 (-3.79 - -3.55) | 51827 (38727,66289) | 13.37 (9.93,17.54) | -2.17 (-2.33 - -2.02) |
| Colombia | Both | 657 (312,1141) | 4.51 (1.69,8.83) | -1.05 (-1.17 - -0.93) | 6660 (5754,7623) | 44.06 (37.66,50.83) | -0.84 (-0.88 - -0.81) | 6 (4,7) | 0.04 (0.03,0.05) | -4.21 (-5.09 - -3.32) | 479 (391,574) | 6.59 (5.19,8.21) | -2.90 (-3.58 - -2.22) |
|  | Female | 376 (185,667) | 5.28 (2,10.15) | -0.92 (-0.95 - -0.90) | 3709 (3195,4260) | 50.33 (42.83,58.21) | -0.76 (-0.79 - -0.72) | 2 (2,3) | 0.03 (0.02,0.04) | -4.80 (-5.84 - -3.74) | 515 (404,639) | 7 (5.34,8.96) | -2.84 (-3.39 - -2.29) |
|  | Male | 281 (128,513) | 3.78 (1.23,7.76) | -1.20 (-1.32 - -1.08) | 2951 (2527,3405) | 38.11 (32.28,44.26) | -0.93 (-0.96 - -0.91) | 3 (3,4) | 0.04 (0.03,0.06) | -4.01 (-5.66 - -2.33) | 994 (813,1212) | 6.2 (4.71,7.94) | -3.18 (-3.93 - -2.42) |
| Comoros | Both | 22 (12,37) | 6.88 (2.98,12.68) | -0.43 (-0.51 - -0.36) | 202 (178,229) | 65.19 (56.79,74.56) | -0.47 (-0.48 - -0.46) | 0 (0,1) | 0.12 (0.07,0.2) | -2.92 (-4.35 - -1.47) | 23 (15,33) | 18.51 (12.32,27.49) | -2.11 (-2.93 - -1.29) |
|  | Female | 12 (7,20) | 7.78 (3.57,14.23) | -0.41 (-0.50 - -0.32) | 118 (104,133) | 76.65 (66.83,87.65) | -0.47 (-0.48 - -0.45) | 0 (0,0) | 0.15 (0.07,0.28) | -3.11 (-4.99 - -1.19) | 35 (24,49) | 22.66 (13.5,36.98) | -1.99 (-2.94 - -1.03) |
|  | Male | 10 (5,17) | 6.03 (2.42,11.48) | -0.45 (-0.54 - -0.35) | 85 (73,98) | 54.04 (45.84,63.1) | -0.47 (-0.48 - -0.46) | 0 (0,0) | 0.1 (0.04,0.19) | -3.17 (-4.16 - -2.17) | 58 (43,78) | 14.46 (8.4,23.98) | -2.27 (-2.85 - -1.69) |
| Congo | Both | 190 (107,322) | 7.67 (3.52,13.95) | -0.39 (-0.49 - -0.30) | 1826 (1607,2051) | 74.57 (64.7,84.55) | -0.37 (-0.39 - -0.36) | 3 (2,4) | 0.12 (0.06,0.2) | -3.37 (-3.90 - -2.84) | 217 (136,312) | 18.98 (12.27,27.8) | -2.31 (-2.62 - -2.00) |
|  | Female | 109 (61,182) | 8.82 (4.03,15.87) | -0.28 (-0.38 - -0.18) | 1079 (949,1216) | 87.95 (76.08,100.26) | -0.29 (-0.31 - -0.28) | 1 (1,2) | 0.11 (0.05,0.18) | -2.74 (-3.00 - -2.48) | 246 (181,324) | 20.18 (12.54,31.18) | -1.70 (-1.82 - -1.57) |
|  | Male | 81 (45,142) | 6.54 (2.85,12.23) | -0.51 (-0.64 - -0.39) | 747 (651,856) | 61.18 (52.66,70.4) | -0.48 (-0.50 - -0.46) | 2 (1,3) | 0.13 (0.05,0.24) | -3.81 (-4.27 - -3.35) | 463 (348,602) | 17.7 (9.65,28.82) | -2.94 (-3.29 - -2.59) |
| Cook Islands | Both | 0 (0,1) | 8.68 (4.03,15.48) | 0.01 (-0.06 - 0.07) | 5 (5,6) | 95.68 (84.26,108.25) | -0.17 (-0.19 - -0.15) | 0 (0,0) | 0.11 (0.03,0.21) | -3.12 (-3.71 - -2.53) | 1 (0,1) | 26.91 (14.76,41.83) | -1.60 (-1.86 - -1.35) |
|  | Female | 0 (0,0) | 9.75 (4.51,17.3) | -0.20 (-0.25 - -0.15) | 3 (3,3) | 105.48 (92,120.11) | -0.40 (-0.44 - -0.36) | 0 (0,0) | 0.15 (0.03,0.31) | -2.96 (-3.46 - -2.47) | 1 (1,1) | 33.38 (15.63,55.75) | -1.67 (-1.90 - -1.44) |
|  | Male | 0 (0,0) | 7.64 (3.48,13.79) | 0.24 (0.13 - 0.35) | 2 (2,3) | 85.78 (74.41,97.76) | 0.08 (0.06 - 0.09) | 0 (0,0) | 0.07 (0.02,0.15) | -3.50 (-4.38 - -2.61) | 1 (1,2) | 20.45 (9.74,34.42) | -1.56 (-1.98 - -1.15) |
| Costa Rica | Both | 73 (36,125) | 5.32 (2.06,10.08) | -0.51 (-0.61 - -0.40) | 743 (642,847) | 52.52 (44.8,60.49) | -0.42 (-0.42 - -0.41) | 0 (0,0) | 0.02 (0.01,0.02) | -3.22 (-3.93 - -2.49) | 32 (24,40) | 5.6 (4.08,7.52) | -1.48 (-1.93 - -1.03) |
|  | Female | 42 (22,75) | 6.27 (2.5,11.98) | -0.54 (-0.65 - -0.43) | 423 (364,487) | 60.26 (51.35,69.95) | -0.50 (-0.52 - -0.49) | 0 (0,0) | 0.02 (0.01,0.02) | -3.59 (-4.37 - -2.81) | 47 (34,64) | 6.76 (4.62,9.52) | -1.41 (-1.61 - -1.21) |
|  | Male | 30 (14,53) | 4.39 (1.56,8.56) | -0.44 (-0.54 - -0.34) | 321 (277,372) | 44.91 (38.04,52.02) | -0.30 (-0.31 - -0.29) | 0 (0,0) | 0.01 (0.01,0.02) | -2.96 (-3.29 - -2.62) | 79 (59,102) | 4.46 (3.32,5.74) | -1.46 (-2.26 - -0.65) |
| Croatia | Both | 36 (17,63) | 4.4 (1.58,8.8) | -0.65 (-0.82 - -0.49) | 337 (289,383) | 40.15 (33.87,46.2) | -0.55 (-0.59 - -0.51) | 0 (0,0) | 0.01 (0.01,0.01) | -5.51 (-6.83 - -4.17) | 27 (19,35) | 8.21 (5.63,11.16) | -1.53 (-1.78 - -1.29) |
|  | Female | 21 (10,37) | 5.3 (1.92,10.42) | -0.56 (-0.69 - -0.43) | 198 (170,229) | 48.66 (41.13,56.49) | -0.51 (-0.56 - -0.46) | 0 (0,0) | 0.01 (0.01,0.01) | -5.63 (-7.71 - -3.51) | 42 (29,57) | 10.3 (6.6,15.04) | -1.31 (-1.65 - -0.98) |
|  | Male | 15 (6,27) | 3.55 (1.07,7.34) | -0.77 (-0.98 - -0.56) | 138 (118,159) | 32.1 (26.95,37.44) | -0.60 (-0.62 - -0.59) | 0 (0,0) | 0.01 (0.01,0.01) | -5.68 (-7.68 - -3.63) | 69 (48,91) | 6.23 (4.45,8.25) | -1.90 (-2.29 - -1.50) |
| Cuba | Both | 135 (67,233) | 5.72 (2.34,10.78) | -0.39 (-0.52 - -0.26) | 1508 (1326,1696) | 60.98 (53.17,69.39) | -0.25 (-0.28 - -0.22) | 1 (1,1) | 0.03 (0.02,0.03) | -3.35 (-4.04 - -2.65) | 73 (58,88) | 7.3 (5.46,9.42) | -1.52 (-1.67 - -1.38) |
|  | Female | 82 (43,144) | 7.17 (3.02,13.39) | -0.30 (-0.36 - -0.23) | 906 (801,1027) | 75.76 (65.92,86.48) | -0.20 (-0.23 - -0.16) | 0 (0,0) | 0.03 (0.02,0.03) | -3.72 (-5.40 - -2.00) | 107 (77,140) | 8.97 (6.22,12.15) | -1.39 (-1.59 - -1.20) |
|  | Male | 53 (23,94) | 4.36 (1.5,8.7) | -0.49 (-0.61 - -0.37) | 602 (521,682) | 47.13 (40.35,54.02) | -0.32 (-0.36 - -0.28) | 0 (0,0) | 0.03 (0.02,0.04) | -2.96 (-3.47 - -2.45) | 180 (138,228) | 5.73 (4.43,7.19) | -1.70 (-1.94 - -1.47) |
| Cyprus | Both | 10 (4,17) | 3.35 (1.03,7) | -0.39 (-0.54 - -0.23) | 101 (83,120) | 35.9 (29.05,43.3) | -0.22 (-0.24 - -0.20) | 0 (0,0) | 0.01 (0,0.01) | -7.53 (-9.04 - -5.99) | 6 (4,8) | 5.23 (3.61,7.21) | -2.35 (-2.87 - -1.83) |
|  | Female | 5 (2,9) | 3.6 (1.05,7.49) | -0.38 (-0.50 - -0.25) | 55 (45,65) | 40.66 (32.29,49.51) | -0.12 (-0.17 - -0.07) | 0 (0,0) | 0.01 (0,0.02) | -7.27 (-9.20 - -5.30) | 9 (6,12) | 6.37 (4.17,9.37) | -2.23 (-2.85 - -1.60) |
|  | Male | 5 (2,8) | 3.13 (0.83,6.54) | -0.43 (-0.65 - -0.22) | 46 (38,54) | 31.48 (25.71,37.77) | -0.31 (-0.37 - -0.26) | 0 (0,0) | 0.01 (0,0.01) | -7.66 (-8.91 - -6.40) | 15 (11,20) | 4.17 (2.94,5.63) | -2.52 (-2.87 - -2.16) |
| Czechia | Both | 107 (50,186) | 4.85 (1.77,9.3) | -0.80 (-0.93 - -0.67) | 982 (846,1126) | 44.61 (37.98,51.63) | -0.54 (-0.56 - -0.52) | 0 (0,0) | 0.01 (0.01,0.01) | -6.42 (-7.33 - -5.49) | 75 (54,99) | 9.16 (5.94,13.39) | -1.85 (-2.10 - -1.60) |
|  | Female | 66 (32,117) | 6.09 (2.32,11.55) | -0.61 (-0.76 - -0.45) | 609 (525,700) | 56.75 (48.42,66.06) | -0.40 (-0.45 - -0.35) | 0 (0,0) | 0.01 (0.01,0.01) | -5.96 (-6.51 - -5.40) | 126 (79,187) | 11.78 (6.59,18.85) | -1.46 (-1.57 - -1.35) |
|  | Male | 42 (19,76) | 3.67 (1.12,7.54) | -1.08 (-1.17 - -0.99) | 373 (317,426) | 33.08 (27.78,38.65) | -0.75 (-0.77 - -0.73) | 0 (0,0) | 0.01 (0.01,0.02) | -6.68 (-7.31 - -6.04) | 201 (137,281) | 6.66 (4.78,8.76) | -2.54 (-2.81 - -2.26) |
| C么te d'Ivoire | Both | 1645 (1087,2546) | 11.43 (6.37,18.93) | -0.36 (-0.40 - -0.31) | 14338 (12939,15712) | 107.76 (96.33,119.62) | -0.51 (-0.53 - -0.49) | 76 (37,118) | 0.49 (0.22,0.84) | -1.22 (-1.53 - -0.91) | 5658 (2690,8983) | 55.04 (30.47,86.29) | -1.09 (-1.38 - -0.81) |
|  | Female | 920 (609,1408) | 12.55 (6.79,20.41) | -0.26 (-0.30 - -0.22) | 9287 (8430,10188) | 142.38 (127.63,157.09) | -0.42 (-0.44 - -0.41) | 16 (10,26) | 0.23 (0.12,0.42) | -0.51 (-0.79 - -0.22) | 2640 (1966,3707) | 38.24 (22.45,59.87) | -0.49 (-0.72 - -0.25) |
|  | Male | 725 (453,1145) | 10.34 (5.36,17.46) | -0.51 (-0.60 - -0.43) | 5051 (4444,5687) | 75.47 (65.75,86.13) | -0.61 (-0.64 - -0.58) | 59 (25,96) | 0.73 (0.27,1.32) | -1.36 (-1.69 - -1.04) | 8298 (4994,11949) | 71.05 (30.7,120.97) | -1.31 (-1.62 - -1.00) |
| Democratic People's Republic of Korea | Both | 510 (297,849) | 7.7 (3.55,13.95) | -0.39 (-0.46 - -0.33) | 4978 (4404,5581) | 72 (62.91,81.36) | -0.75 (-0.76 - -0.73) | 6 (4,9) | 0.09 (0.05,0.14) | -1.87 (-2.06 - -1.68) | 662 (463,894) | 20.4 (13.69,29.01) | -1.22 (-1.36 - -1.07) |
|  | Female | 286 (164,476) | 8.87 (4.03,15.76) | -0.37 (-0.45 - -0.28) | 2855 (2524,3210) | 84.47 (73.66,96.21) | -0.67 (-0.68 - -0.66) | 2 (1,4) | 0.06 (0.03,0.12) | -2.79 (-3.00 - -2.58) | 753 (524,1049) | 22.27 (13.12,34.03) | -1.32 (-1.52 - -1.13) |
|  | Male | 224 (124,382) | 6.58 (2.93,12.12) | -0.40 (-0.46 - -0.34) | 2122 (1850,2402) | 60.05 (51.9,68.79) | -0.77 (-0.79 - -0.75) | 4 (2,6) | 0.11 (0.05,0.19) | -1.30 (-1.43 - -1.17) | 1415 (1043,1855) | 18.61 (11.32,28.05) | -1.09 (-1.24 - -0.93) |
| Democratic Republic of the Congo | Both | 3046 (1614,5255) | 6.37 (2.71,11.77) | -0.70 (-0.87 - -0.53) | 26400 (23107,30076) | 58.31 (49.95,67.07) | -0.75 (-0.80 - -0.71) | 46 (24,76) | 0.1 (0.04,0.18) | -5.43 (-5.66 - -5.20) | 3477 (2216,5222) | 15.39 (9.32,23.99) | -4.08 (-4.24 - -3.92) |
|  | Female | 1730 (954,3054) | 7.32 (3.1,13.76) | -0.58 (-0.78 - -0.37) | 15382 (13337,17500) | 68.88 (58.71,79.59) | -0.61 (-0.63 - -0.59) | 21 (8,39) | 0.09 (0.03,0.18) | -5.47 (-5.77 - -5.18) | 3798 (2472,5629) | 16.61 (9.26,27.78) | -3.80 (-3.98 - -3.61) |
|  | Male | 1316 (675,2253) | 5.45 (2.23,10.32) | -0.85 (-0.99 - -0.71) | 11018 (9622,12622) | 48.07 (41.08,55.71) | -0.95 (-0.97 - -0.92) | 26 (11,47) | 0.1 (0.04,0.21) | -5.38 (-5.55 - -5.21) | 7275 (5012,10286) | 14.2 (8.01,23.55) | -4.34 (-4.49 - -4.20) |
| Denmark | Both | 55 (25,97) | 4.24 (1.5,8.34) | -0.89 (-0.94 - -0.84) | 584 (501,668) | 43.73 (36.86,50.61) | -0.84 (-0.86 - -0.81) | 0 (0,0) | 0.01 (0.01,0.01) | -8.52 (-9.31 - -7.72) | 36 (26,47) | 6.39 (4.51,8.68) | -3.37 (-3.56 - -3.19) |
|  | Female | 29 (13,51) | 4.63 (1.69,9.06) | -0.76 (-0.94 - -0.59) | 318 (274,366) | 49.01 (41.37,57.2) | -0.77 (-0.80 - -0.74) | 0 (0,0) | 0.01 (0.01,0.01) | -8.22 (-9.39 - -7.04) | 49 (35,67) | 7.6 (5.14,10.73) | -2.94 (-3.09 - -2.79) |
|  | Male | 26 (12,47) | 3.87 (1.22,7.86) | -1.04 (-1.10 - -0.97) | 265 (224,307) | 38.72 (32.31,45.52) | -0.92 (-0.95 - -0.88) | 0 (0,0) | 0.01 (0.01,0.01) | -8.84 (-10.52 - -7.12) | 85 (62,114) | 5.24 (3.77,6.98) | -3.83 (-4.05 - -3.61) |
| Djibouti | Both | 40 (23,67) | 7.55 (3.43,13.54) | -0.06 (-0.10 - -0.03) | 359 (318,405) | 70.79 (61.47,80.27) | -0.18 (-0.20 - -0.16) | 0 (0,1) | 0.07 (0.04,0.12) | -1.81 (-2.24 - -1.38) | 39 (28,55) | 15.02 (10.2,21.4) | -1.04 (-1.27 - -0.81) |
|  | Female | 20 (12,35) | 8.36 (3.78,15.02) | -0.05 (-0.09 - -0.00) | 190 (168,215) | 83.28 (72.42,95.22) | -0.14 (-0.16 - -0.13) | 0 (0,0) | 0.06 (0.03,0.12) | -2.17 (-2.65 - -1.69) | 38 (27,51) | 16.39 (9.64,25.52) | -1.03 (-1.25 - -0.80) |
|  | Male | 19 (11,34) | 6.84 (3.01,12.69) | -0.07 (-0.12 - -0.02) | 169 (146,191) | 60.53 (51.9,69.52) | -0.18 (-0.20 - -0.17) | 0 (0,0) | 0.08 (0.04,0.15) | -1.53 (-2.15 - -0.92) | 77 (58,99) | 13.92 (8.57,21.05) | -1.01 (-1.50 - -0.52) |
| Dominica | Both | 1 (1,2) | 5.48 (2.23,10.55) | -0.34 (-0.43 - -0.26) | 12 (11,14) | 56.92 (49.48,64.83) | -0.29 (-0.30 - -0.28) | 0 (0,0) | 0.06 (0.03,0.09) | -0.24 (-0.87 - 0.39) | 1 (0,1) | 9.48 (6.77,12.88) | -0.24 (-0.59 - 0.10) |
|  | Female | 1 (0,1) | 6.91 (2.93,12.88) | -0.27 (-0.40 - -0.14) | 7 (6,8) | 70.72 (61.18,80.81) | -0.24 (-0.26 - -0.22) | 0 (0,0) | 0.08 (0.04,0.13) | -0.38 (-0.82 - 0.05) | 1 (1,2) | 13 (8.77,18.44) | -0.23 (-0.63 - 0.17) |
|  | Male | 0 (0,1) | 4.11 (1.33,8.32) | -0.55 (-0.70 - -0.40) | 5 (4,5) | 43.76 (37.39,50.36) | -0.40 (-0.44 - -0.35) | 0 (0,0) | 0.04 (0.02,0.06) | -0.30 (-0.86 - 0.26) | 2 (1,2) | 6.1 (4.2,8.65) | -0.31 (-0.55 - -0.07) |
| Dominican Republic | Both | 237 (130,389) | 6.07 (2.68,11.04) | -0.17 (-0.35 - 0.00) | 2320 (2021,2620) | 60.15 (51.98,68.58) | -0.30 (-0.31 - -0.28) | 5 (3,7) | 0.12 (0.07,0.2) | -2.25 (-3.04 - -1.45) | 264 (187,372) | 14.99 (10.12,21.39) | -1.81 (-2.37 - -1.24) |
|  | Female | 144 (82,241) | 7.48 (3.33,13.82) | -0.01 (-0.11 - 0.10) | 1395 (1217,1585) | 73.36 (63.5,84.06) | -0.16 (-0.17 - -0.15) | 2 (2,3) | 0.12 (0.06,0.2) | -2.10 (-2.78 - -1.42) | 323 (240,422) | 16.76 (10.67,24.27) | -1.46 (-1.75 - -1.17) |
|  | Male | 93 (48,161) | 4.7 (1.87,8.97) | -0.35 (-0.46 - -0.24) | 925 (800,1043) | 47.3 (40.53,54.22) | -0.44 (-0.45 - -0.43) | 2 (2,4) | 0.13 (0.06,0.24) | -2.32 (-3.17 - -1.45) | 587 (449,739) | 13.31 (7.87,22.2) | -2.16 (-2.79 - -1.53) |
| Ecuador | Both | 344 (180,580) | 5.23 (2.16,9.68) | -0.38 (-0.42 - -0.34) | 3064 (2671,3477) | 46.61 (40.02,53.64) | -0.35 (-0.36 - -0.33) | 4 (3,5) | 0.07 (0.05,0.09) | -4.85 (-6.16 - -3.52) | 290 (240,349) | 9.31 (7.21,11.97) | -3.68 (-4.70 - -2.65) |
|  | Female | 188 (96,331) | 5.8 (2.32,11.18) | -0.44 (-0.50 - -0.38) | 1676 (1457,1901) | 51.79 (44.13,59.84) | -0.38 (-0.39 - -0.37) | 2 (2,2) | 0.06 (0.05,0.09) | -4.51 (-5.81 - -3.19) | 319 (258,394) | 9.89 (7.4,13.16) | -3.15 (-4.05 - -2.24) |
|  | Male | 156 (83,253) | 4.67 (1.88,8.65) | -0.32 (-0.36 - -0.28) | 1388 (1194,1583) | 41.58 (35.28,48.11) | -0.30 (-0.32 - -0.28) | 2 (2,3) | 0.07 (0.05,0.1) | -4.97 (-7.00 - -2.90) | 609 (508,732) | 8.75 (6.57,11.28) | -4.19 (-5.97 - -2.38) |
| Egypt | Both | 4231 (2578,6749) | 9.24 (4.76,15.95) | 0.14 (0.06 - 0.22) | 36373 (32422,40223) | 82.83 (72.98,92.72) | -0.16 (-0.17 - -0.15) | 249 (154,403) | 0.55 (0.31,0.92) | -4.65 (-5.27 - -4.03) | 12051 (7825,19184) | 55.35 (35.58,85.38) | -4.31 (-4.96 - -3.65) |
|  | Female | 2277 (1393,3580) | 10.36 (5.35,17.91) | -0.26 (-0.39 - -0.13) | 19287 (17065,21321) | 91.56 (79.96,103.17) | -0.46 (-0.49 - -0.44) | 127 (73,202) | 0.58 (0.28,1.01) | -5.44 (-6.11 - -4.77) | 13224 (8658,19694) | 60.18 (34.38,95.3) | -5.01 (-5.62 - -4.40) |
|  | Male | 1954 (1199,3179) | 8.2 (4.04,14.45) | 0.73 (0.61 - 0.84) | 17086 (15160,19100) | 74.79 (65.38,84.74) | 0.23 (0.22 - 0.25) | 122 (70,213) | 0.52 (0.24,0.99) | -3.50 (-4.08 - -2.92) | 25275 (17556,37234) | 50.88 (28.47,89.64) | -3.28 (-3.78 - -2.77) |
| El Salvador | Both | 109 (51,193) | 4.57 (1.67,8.95) | -0.74 (-0.88 - -0.61) | 1038 (893,1179) | 43.9 (37.22,50.55) | -0.59 (-0.61 - -0.57) | 1 (1,2) | 0.06 (0.04,0.09) | -4.74 (-6.13 - -3.33) | 98 (74,125) | 8.35 (6.23,11.11) | -3.40 (-4.18 - -2.60) |
|  | Female | 62 (29,111) | 5.33 (1.92,10.51) | -0.60 (-0.67 - -0.52) | 580 (497,662) | 50.14 (42.59,58.05) | -0.53 (-0.56 - -0.50) | 1 (0,1) | 0.05 (0.03,0.08) | -4.71 (-6.16 - -3.23) | 98 (76,123) | 8.51 (6.05,11.51) | -3.13 (-3.84 - -2.41) |
|  | Male | 47 (22,83) | 3.85 (1.34,7.58) | -0.88 (-0.97 - -0.79) | 458 (394,521) | 37.92 (31.96,43.96) | -0.66 (-0.68 - -0.63) | 1 (1,1) | 0.07 (0.04,0.12) | -4.37 (-6.50 - -2.19) | 197 (157,240) | 8.21 (5.45,12.08) | -3.62 (-4.99 - -2.23) |
| Equatorial Guinea | Both | 57 (32,96) | 7.42 (3.31,13.41) | -0.40 (-0.48 - -0.32) | 555 (487,625) | 71.55 (61.84,81.36) | -0.30 (-0.30 - -0.29) | 1 (0,2) | 0.15 (0.05,0.3) | -3.37 (-3.94 - -2.80) | 84 (47,129) | 21.11 (12.14,34.57) | -2.49 (-2.88 - -2.09) |
|  | Female | 29 (16,50) | 8.64 (3.88,15.57) | -0.29 (-0.34 - -0.23) | 285 (249,322) | 86.08 (74.57,98.12) | -0.17 (-0.18 - -0.16) | 0 (0,1) | 0.14 (0.05,0.35) | -3.23 (-3.91 - -2.54) | 77 (50,117) | 23.26 (12.76,41.05) | -2.17 (-2.59 - -1.76) |
|  | Male | 28 (15,48) | 6.44 (2.67,11.76) | -0.43 (-0.56 - -0.30) | 270 (235,308) | 60.64 (51.86,70.18) | -0.28 (-0.29 - -0.26) | 1 (0,1) | 0.15 (0.04,0.32) | -3.47 (-3.97 - -2.98) | 161 (104,235) | 19.77 (9.3,34.8) | -2.72 (-3.09 - -2.34) |
| Eritrea | Both | 198 (105,340) | 6.21 (2.62,11.47) | -0.28 (-0.36 - -0.19) | 1736 (1514,1961) | 56.93 (48.85,65.15) | -0.28 (-0.29 - -0.27) | 3 (2,4) | 0.09 (0.05,0.14) | -1.94 (-2.22 - -1.67) | 211 (152,305) | 14.32 (9.85,20.72) | -1.24 (-1.38 - -1.09) |
|  | Female | 107 (58,184) | 6.86 (2.94,12.83) | -0.18 (-0.31 - -0.05) | 981 (854,1114) | 66.51 (56.93,76.22) | -0.19 (-0.20 - -0.18) | 1 (1,2) | 0.08 (0.04,0.16) | -2.02 (-2.29 - -1.74) | 236 (168,333) | 15.76 (8.98,25.01) | -1.13 (-1.31 - -0.96) |
|  | Male | 92 (47,160) | 5.59 (2.23,10.42) | -0.39 (-0.43 - -0.35) | 755 (654,861) | 47.99 (40.69,55.73) | -0.40 (-0.42 - -0.39) | 1 (1,3) | 0.09 (0.05,0.18) | -1.89 (-2.10 - -1.68) | 447 (338,585) | 12.99 (8.53,20.26) | -1.36 (-1.52 - -1.20) |
| Estonia | Both | 14 (7,25) | 5.04 (1.9,9.79) | -0.43 (-0.56 - -0.29) | 119 (102,136) | 42.48 (36.13,49.11) | -0.12 (-0.19 - -0.04) | 0 (0,0) | 0 (0,0.01) | -8.63 (-10.69 - -6.53) | 8 (6,11) | 8.33 (5.34,12.05) | -1.55 (-1.76 - -1.33) |
|  | Female | 9 (4,16) | 6.42 (2.48,12.44) | 0.06 (-0.09 - 0.20) | 74 (64,85) | 54.42 (46.39,63.14) | 0.07 (0.03 - 0.12) | 0 (0,0) | 0.01 (0,0.01) | -7.84 (-9.12 - -6.55) | 15 (10,21) | 11.13 (6.01,17.39) | -1.09 (-1.24 - -0.94) |
|  | Male | 5 (2,10) | 3.74 (1.11,7.69) | -1.11 (-1.29 - -0.94) | 45 (38,52) | 31.11 (26.03,36.66) | -0.40 (-0.41 - -0.38) | 0 (0,0) | 0 (0,0.01) | -9.46 (-10.39 - -8.52) | 23 (16,32) | 5.67 (3.99,7.64) | -2.29 (-2.55 - -2.04) |
| Eswatini | Both | 35 (19,60) | 6.47 (2.81,12.06) | -0.11 (-0.22 - -0.01) | 324 (286,365) | 61.72 (53.57,70.32) | -0.15 (-0.17 - -0.13) | 1 (0,1) | 0.1 (0.06,0.17) | -0.36 (-0.64 - -0.08) | 37 (27,49) | 16.21 (11.1,22.53) | -0.34 (-0.51 - -0.17) |
|  | Female | 21 (12,37) | 8.24 (3.61,15.17) | -0.14 (-0.32 - 0.04) | 208 (184,234) | 81.5 (70.94,92.77) | -0.17 (-0.19 - -0.15) | 0 (0,0) | 0.1 (0.05,0.19) | -0.52 (-0.88 - -0.15) | 49 (35,64) | 18.95 (11.21,29.26) | -0.36 (-0.60 - -0.12) |
|  | Male | 13 (7,23) | 4.8 (1.82,9.34) | 0.07 (-0.06 - 0.20) | 116 (100,134) | 43.08 (36.54,49.94) | 0.05 (0.04 - 0.05) | 0 (0,0) | 0.11 (0.06,0.19) | -0.03 (-0.30 - 0.24) | 85 (64,108) | 13.63 (9.14,20.05) | -0.10 (-0.28 - 0.09) |
| Ethiopia | Both | 2953 (1445,5184) | 5.18 (2.03,9.88) | -0.93 (-1.16 - -0.70) | 25721 (20608,31795) | 46.66 (36.63,58.37) | -0.76 (-0.79 - -0.74) | 33 (22,48) | 0.06 (0.04,0.09) | -2.01 (-2.19 - -1.83) | 2158 (1552,2936) | 10.68 (7.59,14.61) | -1.39 (-1.47 - -1.30) |
|  | Female | 1676 (847,2925) | 6 (2.43,11.33) | -0.89 (-1.07 - -0.71) | 15111 (12164,18472) | 55.97 (44.14,69.73) | -0.71 (-0.74 - -0.69) | 22 (12,33) | 0.08 (0.04,0.13) | -1.94 (-2.15 - -1.73) | 3866 (2806,5066) | 14.02 (9.42,19.76) | -1.35 (-1.45 - -1.26) |
|  | Male | 1277 (601,2308) | 4.4 (1.57,8.59) | -1.04 (-1.25 - -0.84) | 10610 (8376,13107) | 37.76 (29.13,47.59) | -0.84 (-0.86 - -0.82) | 11 (6,18) | 0.04 (0.02,0.07) | -2.13 (-2.27 - -1.99) | 6024 (4620,7683) | 7.47 (5.02,10.87) | -1.43 (-1.52 - -1.33) |
| Fiji | Both | 35 (22,54) | 9.92 (5.13,16.87) | -0.24 (-0.28 - -0.19) | 363 (324,401) | 105.49 (93.37,117.98) | -0.34 (-0.36 - -0.31) | 1 (1,1) | 0.25 (0.15,0.39) | 0.38 (-0.05 - 0.80) | 67 (49,90) | 40.28 (26.15,57.75) | 0.09 (-0.14 - 0.33) |
|  | Female | 19 (12,29) | 11.17 (5.71,19.21) | -0.26 (-0.32 - -0.20) | 197 (174,221) | 117.41 (103.3,132.32) | -0.38 (-0.39 - -0.36) | 0 (0,1) | 0.25 (0.13,0.41) | 0.29 (-0.12 - 0.70) | 73 (51,98) | 43.21 (25.3,66.3) | -0.09 (-0.29 - 0.11) |
|  | Male | 16 (10,24) | 8.73 (4.43,14.88) | -0.21 (-0.25 - -0.16) | 166 (149,184) | 94.08 (82.94,105.84) | -0.30 (-0.32 - -0.28) | 0 (0,1) | 0.25 (0.14,0.42) | 0.79 (0.00 - 1.58) | 140 (104,182) | 37.4 (21.86,57.76) | 0.30 (-0.13 - 0.74) |
| Finland | Both | 58 (27,103) | 5.07 (1.9,9.74) | -0.74 (-0.90 - -0.59) | 646 (554,738) | 53.54 (45.55,61.6) | -0.30 (-0.35 - -0.26) | 0 (0,0) | 0.01 (0.01,0.02) | -7.61 (-8.52 - -6.69) | 41 (30,54) | 7.98 (5.69,10.88) | -3.00 (-3.21 - -2.79) |
|  | Female | 30 (14,54) | 5.46 (2.1,10.54) | -0.56 (-0.69 - -0.42) | 346 (296,395) | 58.76 (49.61,68.08) | -0.14 (-0.20 - -0.09) | 0 (0,0) | 0.01 (0.01,0.02) | -7.26 (-8.70 - -5.81) | 55 (38,74) | 9.36 (6.13,13.65) | -2.58 (-3.14 - -2.02) |
|  | Male | 28 (13,50) | 4.7 (1.62,9.24) | -0.95 (-1.14 - -0.76) | 300 (257,342) | 48.56 (41.06,56.31) | -0.49 (-0.52 - -0.46) | 0 (0,0) | 0.01 (0.01,0.01) | -8.55 (-9.55 - -7.54) | 96 (71,127) | 6.67 (4.85,8.67) | -3.49 (-3.85 - -3.12) |
| France | Both | 435 (251,672) | 2.78 (1.22,5.04) | -1.00 (-1.06 - -0.94) | 4987 (4360,5689) | 30.43 (26.26,34.85) | -0.92 (-0.95 - -0.90) | 1 (1,1) | 0.01 (0.01,0.01) | -6.22 (-7.30 - -5.14) | 331 (247,431) | 4.48 (3.3,5.84) | -2.19 (-2.43 - -1.95) |
|  | Female | 212 (118,337) | 2.78 (1.19,5.14) | -1.28 (-1.34 - -1.22) | 2564 (2232,2908) | 32.09 (27.58,37.01) | -1.15 (-1.17 - -1.12) | 0 (0,1) | 0.01 (0,0.01) | -6.12 (-8.02 - -4.17) | 399 (293,517) | 5.03 (3.63,6.61) | -2.09 (-2.36 - -1.83) |
|  | Male | 223 (127,337) | 2.77 (1.17,5.01) | -0.69 (-0.75 - -0.63) | 2423 (2103,2784) | 28.86 (24.56,33.49) | -0.67 (-0.69 - -0.66) | 1 (0,1) | 0.01 (0,0.01) | -6.18 (-6.91 - -5.43) | 730 (541,942) | 3.97 (2.92,5.23) | -2.35 (-2.79 - -1.92) |
| Gabon | Both | 60 (34,101) | 7.33 (3.28,13.19) | -0.30 (-0.32 - -0.28) | 580 (510,649) | 71.39 (62.02,80.98) | -0.34 (-0.35 - -0.33) | 1 (0,1) | 0.08 (0.04,0.14) | -2.32 (-2.60 - -2.04) | 60 (42,83) | 15.73 (10.24,23.15) | -1.37 (-1.52 - -1.22) |
|  | Female | 35 (19,61) | 8.17 (3.58,14.81) | -0.28 (-0.31 - -0.26) | 349 (306,395) | 82.53 (71.28,94.01) | -0.27 (-0.28 - -0.26) | 0 (0,0) | 0.06 (0.03,0.12) | -2.81 (-3.10 - -2.52) | 68 (48,92) | 16.16 (9.58,24.88) | -1.35 (-1.49 - -1.22) |
|  | Male | 26 (14,45) | 6.48 (2.77,12.16) | -0.33 (-0.37 - -0.30) | 231 (201,262) | 59.38 (50.9,68.53) | -0.45 (-0.46 - -0.43) | 0 (0,1) | 0.1 (0.05,0.18) | -1.97 (-2.39 - -1.53) | 128 (95,165) | 15.12 (9.04,23.78) | -1.43 (-1.72 - -1.13) |
| Gambia | Both | 133 (82,213) | 10.57 (5.42,18) | -0.54 (-0.63 - -0.46) | 1193 (1073,1318) | 98.6 (87.17,110.32) | -0.72 (-0.76 - -0.68) | 4 (3,6) | 0.33 (0.18,0.53) | -1.57 (-2.65 - -0.48) | 301 (187,452) | 39.11 (24.95,58.18) | -1.41 (-2.19 - -0.63) |
|  | Female | 71 (43,112) | 11.09 (5.43,18.95) | -0.61 (-0.69 - -0.54) | 748 (669,829) | 122.16 (108.43,136.77) | -0.74 (-0.79 - -0.70) | 1 (1,2) | 0.18 (0.08,0.35) | -0.26 (-1.34 - 0.82) | 193 (136,260) | 31.13 (17.97,49.72) | -0.56 (-1.03 - -0.10) |
|  | Male | 62 (37,98) | 10.15 (5.11,17.54) | -0.45 (-0.52 - -0.38) | 445 (388,504) | 75.07 (64.55,85.97) | -0.67 (-0.71 - -0.63) | 3 (2,5) | 0.46 (0.22,0.84) | -1.97 (-3.05 - -0.88) | 493 (354,659) | 46.86 (25.86,78.39) | -1.87 (-2.77 - -0.96) |
| Georgia | Both | 69 (38,119) | 7.57 (3.4,14.1) | -0.48 (-0.52 - -0.43) | 541 (470,615) | 61.23 (52.73,70.5) | -0.48 (-0.51 - -0.46) | 0 (0,0) | 0.03 (0.02,0.03) | -3.60 (-7.66 - 0.64) | 47 (34,61) | 13.31 (8.65,19.15) | -1.21 (-2.62 - 0.21) |
|  | Female | 42 (24,71) | 9.88 (4.62,18.12) | -0.32 (-0.36 - -0.27) | 331 (290,378) | 79.52 (68.48,91.1) | -0.27 (-0.31 - -0.23) | 0 (0,0) | 0.02 (0.01,0.02) | -6.49 (-10.87 - -1.90) | 70 (44,99) | 16.84 (8.84,27.08) | -1.23 (-2.57 - 0.13) |
|  | Male | 26 (13,46) | 5.49 (2.17,10.57) | -0.66 (-0.69 - -0.63) | 210 (177,243) | 44.97 (37.39,53.17) | -0.74 (-0.76 - -0.72) | 0 (0,0) | 0.03 (0.03,0.04) | -2.41 (-5.69 - 0.98) | 116 (80,155) | 10.15 (7.27,13.69) | -1.05 (-2.97 - 0.91) |
| Germany | Both | 905 (439,1574) | 5.67 (2.16,10.87) | -0.82 (-0.93 - -0.72) | 9331 (8117,10591) | 58.3 (49.33,66.86) | -0.42 (-0.45 - -0.40) | 2 (2,2) | 0.01 (0.01,0.01) | -8.33 (-8.79 - -7.87) | 564 (396,760) | 8.46 (5.94,11.78) | -2.94 (-3.11 - -2.77) |
|  | Female | 490 (238,857) | 6.36 (2.48,12.02) | -0.74 (-0.88 - -0.60) | 5175 (4501,5891) | 67.18 (57.16,78.02) | -0.32 (-0.36 - -0.28) | 1 (1,1) | 0.01 (0.01,0.01) | -8.12 (-9.39 - -6.84) | 791 (561,1081) | 10.27 (6.79,15.26) | -2.35 (-2.63 - -2.07) |
|  | Male | 414 (191,757) | 5.03 (1.8,9.99) | -0.91 (-0.99 - -0.84) | 4156 (3565,4803) | 50.04 (42.18,58.42) | -0.54 (-0.56 - -0.52) | 1 (1,1) | 0.01 (0.01,0.01) | -8.69 (-9.73 - -7.64) | 1355 (966,1788) | 6.78 (4.54,9.49) | -3.66 (-3.92 - -3.40) |
| Ghana | Both | 2213 (1484,3325) | 13.76 (7.9,22.17) | 0.13 (0.10 - 0.15) | 19124 (17343,20965) | 123.69 (111.29,137.06) | -0.15 (-0.17 - -0.14) | 42 (29,59) | 0.26 (0.15,0.41) | -3.23 (-3.55 - -2.91) | 3462 (2414,4717) | 36.64 (24.29,52.59) | -2.36 (-2.59 - -2.12) |
|  | Female | 1173 (783,1724) | 14.3 (8.02,23.19) | -0.03 (-0.10 - 0.05) | 12414 (11231,13569) | 161.02 (144.5,177.89) | -0.18 (-0.20 - -0.17) | 10 (5,17) | 0.13 (0.06,0.24) | -2.58 (-2.87 - -2.29) | 2414 (1729,3297) | 31.29 (17.86,48.76) | -1.20 (-1.41 - -0.98) |
|  | Male | 1040 (654,1549) | 13.3 (7.36,21.61) | 0.29 (0.28 - 0.31) | 6709 (5944,7589) | 87.68 (76.46,100.33) | -0.10 (-0.11 - -0.08) | 32 (21,47) | 0.39 (0.21,0.66) | -3.42 (-3.79 - -3.06) | 5876 (4514,7599) | 41.75 (24.74,65.66) | -2.99 (-3.30 - -2.68) |
| Greece | Both | 86 (38,150) | 4.51 (1.63,8.81) | -0.83 (-0.86 - -0.81) | 925 (796,1064) | 46.11 (38.69,53.8) | -0.64 (-0.67 - -0.61) | 0 (0,0) | 0.02 (0.02,0.03) | -6.25 (-7.99 - -4.47) | 65 (49,82) | 7.77 (5.76,10.2) | -2.89 (-3.18 - -2.61) |
|  | Female | 45 (20,81) | 4.88 (1.79,9.56) | -0.74 (-0.78 - -0.71) | 504 (430,578) | 51.41 (43.18,60.02) | -0.59 (-0.61 - -0.57) | 0 (0,0) | 0.02 (0.02,0.03) | -5.63 (-7.25 - -3.98) | 89 (65,116) | 9.22 (6.48,12.58) | -2.49 (-2.74 - -2.23) |
|  | Male | 40 (18,72) | 4.15 (1.42,8.39) | -0.94 (-0.99 - -0.90) | 421 (356,492) | 41.04 (34,48.48) | -0.70 (-0.72 - -0.68) | 0 (0,0) | 0.02 (0.01,0.02) | -6.86 (-8.40 - -5.29) | 154 (115,196) | 6.39 (4.78,8.24) | -3.37 (-3.64 - -3.09) |
| Greenland | Both | 1 (1,2) | 6.78 (3.01,12.4) | -1.09 (-1.23 - -0.95) | 13 (11,15) | 85.01 (74.43,96.34) | -1.23 (-1.30 - -1.16) | 0 (0,0) | 0.04 (0.02,0.06) | -7.75 (-8.19 - -7.30) | 1 (1,2) | 15.02 (10.3,21.17) | -4.31 (-4.53 - -4.09) |
|  | Female | 0 (0,1) | 6.43 (2.61,12.4) | -0.41 (-0.52 - -0.31) | 6 (5,7) | 78.13 (66.85,90.55) | -0.42 (-0.49 - -0.35) | 0 (0,0) | 0.01 (0,0.01) | -5.61 (-6.73 - -4.48) | 1 (1,1) | 12.45 (7.67,19.36) | -1.03 (-1.30 - -0.75) |
|  | Male | 1 (0,1) | 7.1 (3.29,12.91) | -1.57 (-1.68 - -1.47) | 7 (6,8) | 91.55 (80.55,103.38) | -1.75 (-1.81 - -1.68) | 0 (0,0) | 0.07 (0.04,0.11) | -7.98 (-8.44 - -7.51) | 2 (2,3) | 17.39 (11.66,25.24) | -5.46 (-5.76 - -5.17) |
| Grenada | Both | 2 (1,3) | 5.93 (2.54,11.05) | -0.33 (-0.39 - -0.26) | 20 (17,24) | 64.82 (54.01,76.61) | -0.25 (-0.28 - -0.22) | 0 (0,0) | 0.03 (0.02,0.04) | -5.07 (-5.74 - -4.39) | 1 (1,1) | 7.82 (5.69,10.38) | -2.56 (-3.09 - -2.02) |
|  | Female | 1 (1,2) | 7.45 (3.26,13.47) | -0.31 (-0.37 - -0.25) | 12 (10,14) | 78.84 (66.63,91.99) | -0.23 (-0.25 - -0.21) | 0 (0,0) | 0.04 (0.03,0.05) | -5.30 (-6.33 - -4.26) | 2 (1,2) | 10.16 (6.95,14.49) | -2.76 (-3.24 - -2.27) |
|  | Male | 1 (0,1) | 4.52 (1.67,8.81) | -0.32 (-0.48 - -0.16) | 8 (7,10) | 51.93 (41.39,64.4) | -0.24 (-0.28 - -0.21) | 0 (0,0) | 0.02 (0.02,0.03) | -4.62 (-6.58 - -2.61) | 2 (2,3) | 5.66 (4.21,7.31) | -2.41 (-3.44 - -1.38) |
| Guam | Both | 5 (3,8) | 10.05 (5.18,16.88) | 0.77 (0.72 - 0.82) | 54 (49,60) | 111.73 (99.65,124.37) | 0.51 (0.47 - 0.54) | 0 (0,0) | 0.11 (0.07,0.17) | -1.31 (-2.58 - -0.04) | 7 (5,9) | 30.25 (19.41,43.03) | -0.12 (-0.64 - 0.40) |
|  | Female | 3 (2,4) | 12.17 (6.52,20.51) | 0.94 (0.89 - 1.00) | 31 (28,34) | 133.04 (118.4,148.53) | 0.62 (0.58 - 0.66) | 0 (0,0) | 0.08 (0.05,0.12) | -1.94 (-3.89 - 0.06) | 8 (6,11) | 33.55 (18.55,51.71) | -0.04 (-0.80 - 0.73) |
|  | Male | 2 (1,3) | 8.07 (3.87,14) | 0.56 (0.55 - 0.58) | 23 (20,25) | 91.64 (80.26,102.97) | 0.36 (0.33 - 0.40) | 0 (0,0) | 0.14 (0.08,0.23) | -0.89 (-2.31 - 0.55) | 15 (11,18) | 27.16 (16.5,41.3) | -0.21 (-0.75 - 0.32) |
| Guatemala | Both | 291 (142,520) | 4.39 (1.65,8.51) | -0.47 (-0.56 - -0.39) | 2761 (2376,3167) | 40.92 (34.68,47.49) | -0.39 (-0.40 - -0.38) | 5 (4,6) | 0.07 (0.05,0.09) | -3.74 (-4.33 - -3.15) | 273 (220,331) | 8.93 (7.09,10.98) | -2.88 (-3.28 - -2.48) |
|  | Female | 170 (83,307) | 5.15 (1.97,10.15) | -0.34 (-0.37 - -0.32) | 1575 (1348,1819) | 46.82 (39.66,54.91) | -0.22 (-0.23 - -0.21) | 2 (2,3) | 0.07 (0.05,0.09) | -4.14 (-5.08 - -3.20) | 319 (264,381) | 9.6 (7.56,12.02) | -2.98 (-3.60 - -2.36) |
|  | Male | 121 (56,211) | 3.63 (1.18,7.28) | -0.62 (-0.71 - -0.53) | 1186 (1024,1363) | 35.04 (29.69,40.85) | -0.59 (-0.61 - -0.57) | 2 (2,3) | 0.07 (0.05,0.1) | -3.29 (-4.12 - -2.45) | 592 (489,704) | 8.25 (6.24,10.58) | -2.73 (-3.35 - -2.12) |
| Guinea | Both | 755 (485,1177) | 10.09 (5.56,16.76) | -0.12 (-0.14 - -0.10) | 6617 (5958,7261) | 96.09 (85.84,106.2) | -0.24 (-0.25 - -0.23) | 46 (24,76) | 0.57 (0.27,0.98) | -1.57 (-2.05 - -1.08) | 3248 (1559,5293) | 60.6 (33.01,96.23) | -1.39 (-1.80 - -0.98) |
|  | Female | 439 (291,665) | 11.32 (6.33,18.49) | -0.05 (-0.08 - -0.02) | 4425 (3992,4853) | 126.46 (113.34,140.53) | -0.12 (-0.13 - -0.11) | 12 (6,21) | 0.3 (0.14,0.59) | -1.14 (-1.44 - -0.83) | 1570 (995,2418) | 42.16 (23.53,71.29) | -0.83 (-1.03 - -0.62) |
|  | Male | 317 (189,525) | 8.98 (4.53,15.58) | -0.21 (-0.26 - -0.17) | 2192 (1928,2456) | 66.05 (57.34,75.27) | -0.47 (-0.50 - -0.44) | 35 (15,58) | 0.82 (0.33,1.49) | -1.71 (-2.25 - -1.18) | 4817 (2875,7438) | 78.16 (35.23,134.1) | -1.66 (-2.11 - -1.20) |
| Guinea-Bissau | Both | 121 (77,189) | 10.95 (5.91,18.21) | -0.55 (-0.59 - -0.50) | 1037 (936,1142) | 100.05 (88.99,111.37) | -0.67 (-0.71 - -0.64) | 5 (3,7) | 0.43 (0.22,0.73) | -2.90 (-3.93 - -1.85) | 352 (199,540) | 48.06 (29.08,73.32) | -2.51 (-3.49 - -1.52) |
|  | Female | 66 (42,103) | 11.55 (6.08,19.69) | -0.53 (-0.59 - -0.46) | 664 (601,730) | 127.01 (112.79,141.68) | -0.60 (-0.62 - -0.58) | 1 (1,2) | 0.23 (0.11,0.41) | -1.65 (-2.51 - -0.78) | 188 (135,256) | 35.26 (20.15,55.1) | -1.17 (-1.66 - -0.68) |
|  | Male | 55 (34,86) | 10.43 (5.35,17.48) | -0.57 (-0.64 - -0.49) | 373 (327,418) | 73.61 (63.95,83.8) | -0.77 (-0.80 - -0.74) | 4 (2,6) | 0.63 (0.28,1.15) | -3.26 (-4.35 - -2.15) | 540 (369,739) | 60.43 (30.87,103.13) | -3.10 (-4.12 - -2.06) |
| Guyana | Both | 17 (10,29) | 6.2 (2.83,10.84) | -0.90 (-1.00 - -0.81) | 183 (162,205) | 65.82 (57.68,74.31) | -0.89 (-0.94 - -0.85) | 0 (0,0) | 0.1 (0.07,0.14) | -3.62 (-4.63 - -2.60) | 14 (10,17) | 13.89 (10.41,18.16) | -2.93 (-3.63 - -2.22) |
|  | Female | 11 (6,18) | 7.84 (3.78,13.79) | -0.81 (-0.86 - -0.76) | 114 (101,127) | 82.91 (72.79,93.76) | -0.85 (-0.89 - -0.81) | 0 (0,0) | 0.13 (0.09,0.2) | -3.42 (-4.64 - -2.19) | 25 (20,31) | 18.35 (13.03,25.55) | -2.75 (-3.52 - -1.97) |
|  | Male | 7 (3,11) | 4.62 (1.83,8.75) | -1.00 (-1.15 - -0.86) | 69 (60,79) | 49.14 (41.99,56.35) | -0.93 (-0.96 - -0.89) | 0 (0,0) | 0.08 (0.05,0.12) | -3.86 (-5.01 - -2.69) | 39 (31,48) | 9.57 (6.82,13.42) | -3.07 (-3.44 - -2.70) |
| Haiti | Both | 346 (214,538) | 6.06 (3.07,10.61) | -0.41 (-0.44 - -0.39) | 3311 (2978,3677) | 61.01 (54.05,68.17) | -0.35 (-0.37 - -0.33) | 34 (17,63) | 0.58 (0.27,1.12) | -2.53 (-2.79 - -2.27) | 1244 (468,2377) | 54.54 (27.94,100.93) | -2.42 (-2.65 - -2.18) |
|  | Female | 212 (136,336) | 7.5 (3.84,12.77) | -0.22 (-0.26 - -0.18) | 2012 (1816,2224) | 74.59 (66.34,83.77) | -0.21 (-0.24 - -0.18) | 21 (12,42) | 0.73 (0.36,1.5) | -2.38 (-2.70 - -2.06) | 1964 (1154,3749) | 68.17 (37.18,134.05) | -2.27 (-2.55 - -1.98) |
|  | Male | 134 (77,220) | 4.65 (2.05,8.64) | -0.66 (-0.81 - -0.50) | 1299 (1145,1459) | 47.56 (41.44,54.06) | -0.53 (-0.55 - -0.51) | 13 (4,26) | 0.43 (0.12,0.9) | -2.74 (-2.92 - -2.55) | 3208 (1738,5692) | 41.1 (14.02,81.13) | -2.62 (-2.79 - -2.44) |
| Honduras | Both | 224 (112,392) | 5.14 (2.05,9.8) | -0.66 (-0.75 - -0.58) | 2213 (1921,2517) | 50.62 (43.46,58.11) | -0.70 (-0.73 - -0.67) | 3 (2,6) | 0.08 (0.03,0.14) | -4.68 (-4.83 - -4.54) | 219 (131,347) | 10.29 (6.29,16.04) | -3.70 (-3.80 - -3.60) |
|  | Female | 128 (63,230) | 5.96 (2.4,11.26) | -0.59 (-0.69 - -0.49) | 1218 (1050,1391) | 56.36 (48.12,64.93) | -0.62 (-0.65 - -0.60) | 2 (1,3) | 0.07 (0.03,0.14) | -5.13 (-5.32 - -4.93) | 229 (148,341) | 10.63 (6.22,17.25) | -3.87 (-3.99 - -3.74) |
|  | Male | 96 (46,169) | 4.35 (1.62,8.44) | -0.77 (-0.91 - -0.63) | 995 (869,1133) | 44.98 (38.7,51.92) | -0.78 (-0.82 - -0.75) | 2 (1,3) | 0.08 (0.03,0.17) | -4.20 (-4.35 - -4.05) | 448 (293,653) | 9.94 (5.47,17.1) | -3.51 (-3.62 - -3.40) |
| Hungary | Both | 104 (53,181) | 5.5 (2.18,10.4) | -0.70 (-0.80 - -0.60) | 965 (836,1096) | 50.17 (43.05,57.83) | -0.55 (-0.56 - -0.54) | 0 (0,0) | 0.01 (0.01,0.01) | -6.75 (-8.20 - -5.27) | 70 (48,93) | 10.2 (6.7,15.02) | -1.80 (-2.10 - -1.50) |
|  | Female | 66 (36,117) | 7.22 (3.01,13.58) | -0.64 (-0.76 - -0.53) | 615 (536,701) | 65.76 (56.65,75.83) | -0.52 (-0.53 - -0.50) | 0 (0,0) | 0.01 (0.01,0.01) | -6.56 (-9.41 - -3.62) | 127 (83,185) | 13.53 (7.91,21.77) | -1.23 (-1.57 - -0.90) |
|  | Male | 38 (17,68) | 3.87 (1.2,7.86) | -0.78 (-0.89 - -0.66) | 350 (298,407) | 35.41 (29.75,41.52) | -0.60 (-0.62 - -0.59) | 0 (0,0) | 0.01 (0.01,0.02) | -6.63 (-8.65 - -4.57) | 196 (137,269) | 7.04 (4.75,9.97) | -2.61 (-3.18 - -2.03) |
| Iceland | Both | 4 (2,7) | 4.66 (1.67,9.24) | -0.50 (-0.54 - -0.46) | 43 (37,50) | 47.68 (40.25,55.68) | -0.29 (-0.30 - -0.28) | 0 (0,0) | 0.01 (0.01,0.01) | -8.41 (-9.47 - -7.33) | 3 (2,4) | 6.87 (4.85,9.22) | -2.88 (-3.10 - -2.66) |
|  | Female | 2 (1,4) | 4.92 (1.72,9.88) | -0.42 (-0.52 - -0.32) | 23 (20,26) | 51.65 (43.37,60.5) | -0.24 (-0.26 - -0.22) | 0 (0,0) | 0.01 (0.01,0.01) | -8.24 (-10.01 - -6.43) | 3 (2,5) | 7.94 (5.36,11.18) | -2.74 (-3.02 - -2.46) |
|  | Male | 2 (1,4) | 4.42 (1.54,8.95) | -0.59 (-0.65 - -0.53) | 20 (17,23) | 43.87 (36.69,51.32) | -0.35 (-0.37 - -0.32) | 0 (0,0) | 0.01 (0.01,0.01) | -8.89 (-11.02 - -6.71) | 6 (4,8) | 5.84 (4.17,7.86) | -3.30 (-3.97 - -2.62) |
| India | Both | 24136 (11547,43402) | 4.76 (1.85,9.23) | -0.28 (-0.55 - -0.01) | 203503 (162083,252841) | 38.62 (29.88,48.73) | -0.29 (-0.34 - -0.25) | 207 (150,297) | 0.04 (0.03,0.06) | -3.99 (-4.20 - -3.77) | 18265 (14073,24280) | 9.06 (6.52,12.25) | -2.34 (-2.42 - -2.25) |
|  | Female | 14309 (7196,25646) | 5.92 (2.36,11.32) | -0.12 (-0.37 - 0.12) | 120204 (96202,149011) | 47.77 (37.21,59.83) | -0.12 (-0.14 - -0.11) | 116 (67,185) | 0.05 (0.03,0.08) | -4.18 (-4.45 - -3.92) | 27715 (20891,36766) | 11.31 (7.88,16.07) | -2.28 (-2.36 - -2.20) |
|  | Male | 9826 (4357,17678) | 3.71 (1.24,7.62) | -0.46 (-0.51 - -0.42) | 83299 (65474,104156) | 30.25 (23.24,38.4) | -0.52 (-0.55 - -0.48) | 91 (64,153) | 0.04 (0.02,0.06) | -3.68 (-4.23 - -3.13) | 45980 (35618,57968) | 6.99 (4.79,9.84) | -2.38 (-2.69 - -2.07) |
| Indonesia | Both | 6247 (3516,10777) | 6.89 (3.13,12.61) | -0.73 (-0.88 - -0.57) | 55056 (44562,67387) | 59.84 (47.19,74.68) | -0.84 (-0.87 - -0.82) | 176 (101,251) | 0.2 (0.11,0.3) | -1.95 (-2.09 - -1.80) | 11407 (7323,15283) | 27.37 (18.1,37.68) | -1.60 (-1.75 - -1.46) |
|  | Female | 3704 (2148,6383) | 8.41 (3.88,15.38) | -0.66 (-0.77 - -0.55) | 32809 (26730,39829) | 73.48 (58.08,91.28) | -0.76 (-0.77 - -0.74) | 83 (50,130) | 0.19 (0.11,0.31) | -2.49 (-2.81 - -2.16) | 13325 (9623,17511) | 30.28 (20.24,43.74) | -1.81 (-1.90 - -1.71) |
|  | Male | 2543 (1346,4456) | 5.45 (2.29,10.37) | -0.79 (-1.03 - -0.56) | 22247 (17935,27355) | 46.98 (37.04,59.02) | -0.94 (-0.98 - -0.90) | 93 (47,137) | 0.2 (0.09,0.32) | -1.33 (-1.46 - -1.20) | 24731 (17691,31691) | 24.64 (14.57,35.41) | -1.30 (-1.39 - -1.22) |
| Iran (Islamic Republic of) | Both | 2433 (1481,3840) | 9.51 (4.92,16.48) | -1.27 (-1.33 - -1.21) | 21172 (17204,25765) | 83.43 (66.13,103.38) | -1.36 (-1.42 - -1.31) | 53 (41,69) | 0.21 (0.16,0.29) | -4.52 (-4.96 - -4.07) | 3311 (2595,4139) | 27.83 (21.84,34.99) | -3.79 (-4.14 - -3.43) |
|  | Female | 1328 (814,2095) | 10.66 (5.53,18.23) | -1.41 (-1.48 - -1.35) | 11415 (9300,13818) | 92.38 (73.54,114.48) | -1.50 (-1.56 - -1.45) | 26 (19,33) | 0.21 (0.16,0.29) | -4.90 (-5.41 - -4.38) | 3652 (2893,4487) | 29.92 (22.94,37.83) | -3.98 (-4.36 - -3.60) |
|  | Male | 1105 (667,1746) | 8.41 (4.28,14.56) | -1.08 (-1.16 - -1.01) | 9757 (7909,11891) | 74.93 (59.27,93.11) | -1.19 (-1.25 - -1.14) | 27 (21,36) | 0.22 (0.16,0.3) | -4.08 (-4.45 - -3.70) | 6962 (5518,8555) | 25.84 (19.81,33.52) | -3.53 (-3.85 - -3.21) |
| Iraq | Both | 2010 (1193,3175) | 11.44 (5.62,19.78) | -0.18 (-0.28 - -0.08) | 18691 (16619,20860) | 105.8 (93.18,118.86) | -0.10 (-0.13 - -0.07) | 67 (45,89) | 0.39 (0.23,0.58) | -4.01 (-4.39 - -3.62) | 4037 (3058,5311) | 45.64 (30.99,64.53) | -3.28 (-3.63 - -2.93) |
|  | Female | 1103 (667,1730) | 12.95 (6.44,21.99) | -0.13 (-0.21 - -0.06) | 9995 (8917,11146) | 116.79 (102.85,132.12) | -0.03 (-0.05 - -0.02) | 30 (18,44) | 0.36 (0.19,0.61) | -4.62 (-5.06 - -4.17) | 3878 (2818,5110) | 46.04 (28.56,68.51) | -3.64 (-4.02 - -3.27) |
|  | Male | 907 (527,1452) | 10.02 (4.74,17.55) | -0.23 (-0.35 - -0.10) | 8696 (7676,9765) | 95.47 (83.2,108.54) | -0.18 (-0.20 - -0.17) | 37 (26,52) | 0.42 (0.23,0.67) | -3.36 (-3.73 - -2.98) | 7915 (6006,10070) | 45.27 (28.49,67.47) | -2.94 (-3.26 - -2.62) |
| Ireland | Both | 49 (21,90) | 3.73 (1.22,7.6) | -0.90 (-0.99 - -0.81) | 518 (440,598) | 38.15 (31.85,44.51) | -0.71 (-0.74 - -0.68) | 0 (0,0) | 0 (0,0.01) | -8.49 (-9.38 - -7.59) | 30 (22,41) | 5.38 (3.72,7.44) | -2.70 (-3.10 - -2.30) |
|  | Female | 26 (11,48) | 4.03 (1.33,8.24) | -0.96 (-1.06 - -0.86) | 283 (241,328) | 42.55 (35.65,49.85) | -0.72 (-0.75 - -0.70) | 0 (0,0) | 0.01 (0,0.01) | -7.94 (-9.20 - -6.66) | 43 (30,59) | 6.43 (4.21,9.36) | -2.33 (-2.73 - -1.92) |
|  | Male | 23 (10,42) | 3.44 (1.03,6.99) | -0.85 (-0.98 - -0.72) | 236 (200,274) | 33.93 (28.2,40.21) | -0.69 (-0.71 - -0.67) | 0 (0,0) | 0 (0,0.01) | -8.79 (-10.31 - -7.25) | 73 (52,98) | 4.38 (3.09,5.93) | -3.08 (-3.52 - -2.64) |
| Israel | Both | 156 (70,276) | 4.61 (1.71,9.04) | -1.01 (-1.21 - -0.81) | 1525 (1317,1737) | 46.76 (39.6,54.13) | -0.80 (-0.84 - -0.76) | 0 (0,0) | 0 (0,0.01) | -8.99 (-10.16 - -7.80) | 94 (67,125) | 6.49 (4.55,8.99) | -2.69 (-2.85 - -2.52) |
|  | Female | 77 (34,137) | 4.68 (1.69,9.27) | -0.84 (-1.06 - -0.62) | 783 (677,896) | 49.21 (41.55,57.33) | -0.66 (-0.69 - -0.64) | 0 (0,0) | 0.01 (0,0.01) | -9.08 (-10.13 - -8.03) | 118 (83,164) | 7.39 (4.95,10.79) | -2.58 (-2.87 - -2.30) |
|  | Male | 78 (35,143) | 4.53 (1.64,9.08) | -1.14 (-1.33 - -0.94) | 742 (638,851) | 44.43 (37.23,52) | -0.94 (-1.00 - -0.88) | 0 (0,0) | 0 (0,0.01) | -8.94 (-10.20 - -7.67) | 212 (153,283) | 5.64 (3.95,7.6) | -2.82 (-2.99 - -2.65) |
| Italy | Both | 453 (201,818) | 4.32 (1.55,8.54) | -0.91 (-1.02 - -0.80) | 4866 (3885,6023) | 43.48 (33.61,54.55) | -0.74 (-0.78 - -0.69) | 1 (1,1) | 0.01 (0.01,0.01) | -7.37 (-9.09 - -5.62) | 298 (211,401) | 6.54 (4.63,8.86) | -2.68 (-3.12 - -2.25) |
|  | Female | 236 (104,429) | 4.65 (1.68,9.2) | -1.05 (-1.17 - -0.92) | 2639 (2102,3254) | 48.75 (37.92,61.14) | -0.77 (-0.79 - -0.76) | 1 (1,1) | 0.01 (0.01,0.02) | -6.62 (-8.85 - -4.34) | 429 (307,570) | 7.98 (5.62,10.73) | -2.45 (-3.04 - -1.85) |
|  | Male | 217 (98,388) | 4.01 (1.4,8.03) | -0.76 (-0.99 - -0.53) | 2226 (1754,2779) | 38.52 (29.67,48.67) | -0.69 (-0.74 - -0.64) | 0 (0,0) | 0.01 (0.01,0.01) | -7.67 (-8.52 - -6.81) | 727 (522,965) | 5.19 (3.61,7.09) | -3.02 (-3.51 - -2.54) |
| Jamaica | Both | 43 (22,76) | 5.44 (2.24,10.4) | -0.38 (-0.48 - -0.29) | 496 (434,561) | 57.17 (49.56,65.12) | -0.31 (-0.32 - -0.30) | 0 (0,0) | 0.04 (0.03,0.05) | -5.74 (-7.56 - -3.90) | 23 (18,28) | 7.87 (5.92,10.24) | -3.68 (-4.42 - -2.94) |
|  | Female | 27 (14,47) | 6.8 (2.88,12.89) | -0.32 (-0.41 - -0.24) | 302 (264,342) | 70.88 (61.25,80.94) | -0.25 (-0.26 - -0.24) | 0 (0,0) | 0.05 (0.04,0.07) | -6.08 (-8.28 - -3.83) | 44 (34,55) | 10.6 (7.7,14.31) | -3.88 (-4.90 - -2.84) |
|  | Male | 17 (8,30) | 4.13 (1.47,8.28) | -0.45 (-0.51 - -0.38) | 194 (169,221) | 43.92 (37.81,50.58) | -0.36 (-0.38 - -0.34) | 0 (0,0) | 0.03 (0.02,0.04) | -5.33 (-6.06 - -4.59) | 67 (53,82) | 5.24 (3.97,6.78) | -3.33 (-3.86 - -2.79) |
| Japan | Both | 987 (441,1826) | 4.64 (1.69,9.2) | 0.10 (0.03 - 0.18) | 9853 (7868,12228) | 43.92 (34.24,54.98) | 0.10 (0.09 - 0.12) | 2 (2,2) | 0.01 (0.01,0.01) | -5.31 (-5.83 - -4.79) | 716 (498,966) | 7.91 (5.42,10.74) | -0.78 (-0.99 - -0.56) |
|  | Female | 546 (242,1018) | 5.28 (1.94,10.61) | 0.12 (-0.00 - 0.25) | 5590 (4463,6927) | 51.3 (39.84,64.08) | 0.15 (0.12 - 0.18) | 1 (1,1) | 0.01 (0.01,0.01) | -5.35 (-5.91 - -4.78) | 1056 (728,1420) | 9.7 (6.56,13.22) | -0.68 (-0.95 - -0.41) |
|  | Male | 441 (195,794) | 4.03 (1.41,8.15) | 0.06 (-0.02 - 0.13) | 4263 (3389,5294) | 36.94 (28.54,46.47) | 0.07 (0.06 - 0.08) | 1 (1,1) | 0.01 (0.01,0.01) | -5.25 (-5.91 - -4.59) | 1772 (1226,2383) | 6.21 (4.27,8.44) | -1.14 (-1.52 - -0.77) |
| Jordan | Both | 551 (333,911) | 11.2 (5.39,19.73) | -0.37 (-0.49 - -0.26) | 5471 (4855,6083) | 106.57 (93.68,120.04) | -0.35 (-0.39 - -0.31) | 17 (14,21) | 0.37 (0.26,0.49) | -3.59 (-3.85 - -3.33) | 1089 (857,1362) | 45.28 (33.3,59.84) | -2.84 (-3.04 - -2.64) |
|  | Female | 297 (178,494) | 12.46 (6,21.93) | -0.34 (-0.45 - -0.22) | 2854 (2513,3189) | 115.23 (100.51,130.26) | -0.35 (-0.38 - -0.32) | 8 (6,10) | 0.36 (0.24,0.51) | -3.88 (-4.17 - -3.60) | 1103 (865,1371) | 46.88 (31.94,65.12) | -2.99 (-3.21 - -2.77) |
|  | Male | 254 (146,417) | 10.03 (4.62,17.83) | -0.40 (-0.51 - -0.28) | 2617 (2312,2938) | 98.53 (85.8,111.76) | -0.35 (-0.38 - -0.33) | 9 (7,11) | 0.38 (0.24,0.55) | -3.25 (-3.54 - -2.95) | 2192 (1802,2668) | 43.82 (29.49,61.5) | -2.68 (-2.98 - -2.38) |
| Kazakhstan | Both | 497 (279,849) | 7.63 (3.45,14.08) | -0.60 (-0.71 - -0.49) | 4047 (3530,4605) | 65.57 (56.06,75.6) | -0.66 (-0.69 - -0.61) | 2 (2,3) | 0.04 (0.03,0.05) | -3.81 (-5.53 - -2.06) | 367 (276,462) | 15.09 (10,21.22) | -1.47 (-2.01 - -0.93) |
|  | Female | 305 (175,519) | 9.59 (4.29,17.84) | -0.20 (-0.36 - -0.04) | 2488 (2169,2833) | 82.37 (70.62,94.77) | -0.15 (-0.16 - -0.13) | 1 (1,1) | 0.03 (0.02,0.04) | -3.97 (-5.38 - -2.54) | 551 (359,764) | 18.4 (10.31,28.52) | -1.12 (-1.50 - -0.74) |
|  | Male | 192 (103,334) | 5.76 (2.38,10.96) | -1.16 (-1.24 - -1.07) | 1559 (1343,1786) | 49.45 (41.84,57.86) | -1.32 (-1.45 - -1.19) | 1 (1,2) | 0.05 (0.03,0.06) | -3.70 (-5.47 - -1.90) | 918 (653,1211) | 11.91 (8.66,15.69) | -2.04 (-3.15 - -0.92) |
| Kenya | Both | 1765 (955,3067) | 7.18 (3.07,13.22) | -0.58 (-0.81 - -0.35) | 16595 (13425,20288) | 66.83 (52.8,82.72) | -0.58 (-0.61 - -0.54) | 11 (8,15) | 0.05 (0.03,0.06) | -1.76 (-1.97 - -1.56) | 1212 (907,1569) | 12.32 (9.04,16) | -1.06 (-1.08 - -1.03) |
|  | Female | 985 (545,1711) | 8.13 (3.54,14.94) | -0.52 (-0.78 - -0.27) | 9642 (7829,11709) | 78.69 (62.53,97.2) | -0.53 (-0.58 - -0.49) | 6 (4,10) | 0.05 (0.03,0.09) | -2.26 (-2.53 - -1.98) | 1819 (1332,2350) | 14.98 (10.58,20.04) | -1.19 (-1.26 - -1.13) |
|  | Male | 780 (411,1368) | 6.25 (2.63,11.76) | -0.63 (-0.84 - -0.41) | 6953 (5576,8571) | 55.28 (43.29,69.23) | -0.62 (-0.66 - -0.58) | 5 (3,7) | 0.04 (0.03,0.06) | -0.95 (-1.05 - -0.86) | 3030 (2316,3843) | 9.73 (7.08,12.97) | -0.79 (-0.82 - -0.75) |
| Kiribati | Both | 5 (3,8) | 10.09 (5.54,16.42) | -0.68 (-0.74 - -0.63) | 51 (46,56) | 98.2 (87.34,108.74) | -0.92 (-0.97 - -0.86) | 0 (0,0) | 0.36 (0.19,0.58) | -2.36 (-2.68 - -2.03) | 15 (9,21) | 48.63 (30.16,70.96) | -1.96 (-2.19 - -1.73) |
|  | Female | 3 (2,4) | 10.9 (5.69,18.46) | -0.81 (-0.87 - -0.74) | 28 (25,31) | 108.73 (96.44,121.77) | -1.00 (-1.06 - -0.94) | 0 (0,0) | 0.27 (0.13,0.49) | -2.80 (-3.00 - -2.60) | 11 (8,16) | 44.16 (24.53,71.07) | -2.16 (-2.27 - -2.04) |
|  | Male | 3 (2,4) | 9.34 (5.25,15.03) | -0.52 (-0.60 - -0.45) | 23 (21,25) | 87.96 (77.7,97.81) | -0.83 (-0.87 - -0.79) | 0 (0,0) | 0.45 (0.21,0.76) | -2.07 (-2.44 - -1.71) | 26 (18,35) | 52.63 (28.84,83.15) | -1.83 (-2.12 - -1.53) |
| Kuwait | Both | 114 (67,190) | 10.43 (5,18.59) | -0.29 (-0.47 - -0.11) | 1097 (979,1223) | 100.52 (88.24,113.2) | -0.37 (-0.42 - -0.32) | 3 (2,3) | 0.26 (0.2,0.33) | -0.63 (-1.18 - -0.08) | 221 (182,267) | 35.58 (27.48,45.65) | -0.61 (-0.97 - -0.26) |
|  | Female | 61 (36,103) | 11.56 (5.49,20.67) | -0.27 (-0.47 - -0.07) | 576 (510,653) | 108.69 (94.46,124.1) | -0.32 (-0.36 - -0.28) | 1 (1,1) | 0.18 (0.14,0.23) | -2.68 (-4.89 - -0.41) | 163 (128,202) | 31.04 (20.58,43.14) | -1.66 (-2.96 - -0.34) |
|  | Male | 52 (30,89) | 9.36 (4.34,16.86) | -0.29 (-0.44 - -0.14) | 522 (463,583) | 92.81 (80.94,105.17) | -0.39 (-0.44 - -0.33) | 2 (2,2) | 0.34 (0.25,0.46) | 0.10 (-0.46 - 0.68) | 385 (322,466) | 39.88 (30.03,51.8) | -0.10 (-0.52 - 0.31) |
| Kyrgyzstan | Both | 145 (77,257) | 5.31 (2.11,10.04) | -0.98 (-1.08 - -0.87) | 1124 (972,1288) | 43.27 (36.78,50.09) | -0.78 (-0.80 - -0.75) | 1 (1,1) | 0.04 (0.03,0.05) | -3.79 (-5.01 - -2.56) | 111 (84,140) | 11.37 (7.98,15.5) | -2.18 (-2.60 - -1.76) |
|  | Female | 89 (47,159) | 6.64 (2.74,12.69) | -0.92 (-1.03 - -0.81) | 694 (597,798) | 54.47 (46.24,63.18) | -0.67 (-0.69 - -0.66) | 1 (0,1) | 0.05 (0.04,0.06) | -4.42 (-7.37 - -1.39) | 178 (126,245) | 14.25 (8.84,21.16) | -2.13 (-2.54 - -1.73) |
|  | Male | 56 (27,103) | 4.04 (1.38,8.15) | -1.05 (-1.17 - -0.92) | 430 (366,502) | 32.5 (27.21,38.37) | -0.92 (-0.94 - -0.89) | 0 (0,1) | 0.04 (0.03,0.05) | -3.48 (-5.13 - -1.81) | 290 (213,378) | 8.6 (6.34,11.43) | -2.25 (-3.06 - -1.43) |
| Lao People's Democratic Republic | Both | 177 (99,301) | 5.94 (2.62,10.78) | -0.29 (-0.33 - -0.25) | 1567 (1377,1765) | 53.94 (46.42,61.57) | -0.33 (-0.35 - -0.32) | 7 (4,10) | 0.23 (0.12,0.37) | -3.54 (-3.88 - -3.21) | 406 (266,596) | 28.96 (18.92,42.25) | -2.83 (-3.11 - -2.56) |
|  | Female | 102 (59,172) | 6.95 (3.03,12.67) | -0.22 (-0.29 - -0.15) | 906 (791,1023) | 63.27 (54.24,72.51) | -0.27 (-0.28 - -0.26) | 3 (2,5) | 0.23 (0.12,0.4) | -4.13 (-4.29 - -3.97) | 465 (336,639) | 31.53 (19.1,48.03) | -3.19 (-3.37 - -3.01) |
|  | Male | 75 (41,128) | 4.96 (2.05,9.31) | -0.38 (-0.41 - -0.34) | 661 (575,754) | 44.87 (38.3,51.43) | -0.40 (-0.43 - -0.38) | 4 (2,6) | 0.23 (0.1,0.42) | -2.89 (-3.17 - -2.61) | 871 (621,1163) | 26.45 (15.07,42.43) | -2.41 (-2.66 - -2.16) |
| Latvia | Both | 19 (9,34) | 4.91 (1.87,9.55) | -0.44 (-0.58 - -0.30) | 169 (145,194) | 43.82 (37.07,51.09) | -0.45 (-0.48 - -0.42) | 0 (0,0) | 0.01 (0.01,0.01) | -7.05 (-8.59 - -5.47) | 12 (9,16) | 8.93 (5.84,12.78) | -1.90 (-2.31 - -1.49) |
|  | Female | 12 (6,21) | 6.23 (2.41,11.81) | -0.37 (-0.51 - -0.23) | 105 (90,121) | 56.12 (47.4,65.66) | -0.41 (-0.44 - -0.38) | 0 (0,0) | 0.01 (0.01,0.01) | -6.79 (-9.92 - -3.56) | 22 (15,32) | 11.81 (6.75,18.38) | -1.59 (-2.09 - -1.09) |
|  | Male | 7 (3,14) | 3.67 (1.09,7.54) | -0.53 (-0.72 - -0.34) | 64 (54,74) | 32.2 (26.78,37.91) | -0.50 (-0.53 - -0.47) | 0 (0,0) | 0.01 (0.01,0.01) | -7.07 (-9.24 - -4.84) | 34 (24,47) | 6.2 (4.4,8.13) | -2.34 (-2.70 - -1.98) |
| Lebanon | Both | 183 (113,283) | 11.06 (5.64,18.81) | 0.21 (0.15 - 0.28) | 1637 (1455,1829) | 98.5 (86.44,110.81) | 0.16 (0.14 - 0.18) | 1 (1,2) | 0.07 (0.05,0.11) | -4.06 (-4.19 - -3.94) | 153 (114,203) | 19.41 (13.24,26.99) | -1.86 (-1.92 - -1.80) |
|  | Female | 93 (56,150) | 11.76 (5.71,20.61) | 0.35 (0.24 - 0.47) | 830 (730,933) | 105.22 (91.44,119.47) | 0.29 (0.26 - 0.32) | 1 (0,1) | 0.07 (0.04,0.11) | -4.99 (-5.26 - -4.71) | 168 (116,225) | 21.38 (12.97,32.69) | -2.25 (-2.35 - -2.14) |
|  | Male | 90 (57,138) | 10.42 (5.48,17.57) | 0.12 (0.04 - 0.21) | 807 (719,902) | 92.43 (80.52,104.48) | 0.04 (0.02 - 0.06) | 1 (0,1) | 0.08 (0.04,0.13) | -2.92 (-3.14 - -2.70) | 320 (247,412) | 17.64 (11.21,26.11) | -1.42 (-1.59 - -1.26) |
| Lesotho | Both | 47 (25,82) | 5.66 (2.4,10.74) | -0.22 (-0.37 - -0.06) | 455 (396,517) | 53.48 (45.8,61.67) | -0.19 (-0.20 - -0.18) | 1 (1,1) | 0.09 (0.05,0.15) | 1.91 (1.31 - 2.52) | 48 (33,75) | 14.28 (9.7,20.64) | 0.68 (0.43 - 0.92) |
|  | Female | 30 (17,52) | 7.31 (3.19,13.63) | -0.26 (-0.39 - -0.13) | 301 (262,345) | 71.47 (61.47,82.51) | -0.19 (-0.22 - -0.17) | 0 (0,1) | 0.09 (0.04,0.17) | 1.41 (0.35 - 2.48) | 72 (52,95) | 17.21 (10.66,26.55) | 0.37 (0.15 - 0.60) |
|  | Male | 17 (8,30) | 4.03 (1.4,7.99) | -0.14 (-0.24 - -0.04) | 153 (131,176) | 35.77 (30.17,41.81) | -0.07 (-0.07 - -0.07) | 0 (0,1) | 0.09 (0.04,0.18) | 2.62 (2.10 - 3.15) | 120 (90,156) | 11.41 (6.89,18.61) | 1.30 (1.15 - 1.46) |
| Liberia | Both | 265 (160,433) | 9.52 (4.82,16.46) | -0.66 (-0.80 - -0.51) | 2352 (2100,2605) | 87.53 (77.2,97.93) | -0.86 (-0.88 - -0.83) | 11 (6,18) | 0.38 (0.18,0.67) | -4.04 (-4.92 - -3.16) | 758 (379,1228) | 43.28 (24.27,67.75) | -3.60 (-4.36 - -2.83) |
|  | Female | 149 (91,238) | 10.69 (5.41,18.04) | -0.64 (-0.81 - -0.46) | 1510 (1357,1677) | 113.02 (100.16,126.75) | -0.82 (-0.86 - -0.79) | 3 (2,5) | 0.24 (0.1,0.45) | -2.97 (-3.46 - -2.47) | 463 (315,648) | 34.24 (19.5,56.02) | -2.35 (-2.72 - -1.98) |
|  | Male | 116 (69,202) | 8.43 (3.98,15.2) | -0.68 (-0.75 - -0.61) | 842 (732,954) | 62.76 (53.82,72.38) | -0.81 (-0.83 - -0.78) | 8 (3,13) | 0.52 (0.2,0.96) | -4.44 (-5.31 - -3.57) | 1221 (751,1815) | 51.82 (23.72,89.68) | -4.21 (-5.03 - -3.38) |
| Libya | Both | 254 (170,380) | 12.06 (6.98,19.24) | -0.06 (-0.11 - -0.02) | 2509 (2260,2756) | 111.66 (99.87,123.69) | -0.62 (-0.67 - -0.57) | 21 (13,35) | 1.12 (0.6,2.14) | 0.16 (-0.79 - 1.12) | 1150 (587,1873) | 109.45 (64.1,198.48) | -0.09 (-1.53 - 1.37) |
|  | Female | 147 (98,220) | 14.13 (8.26,22.63) | 0.18 (0.06 - 0.29) | 1390 (1254,1519) | 126.42 (112.51,140.01) | -0.53 (-0.58 - -0.47) | 9 (5,17) | 0.94 (0.46,2.14) | -0.63 (-2.39 - 1.16) | 916 (594,1633) | 96.77 (53.3,201.84) | -0.69 (-2.03 - 0.67) |
|  | Male | 107 (71,162) | 10.08 (5.48,16.18) | -0.30 (-0.36 - -0.24) | 1119 (1003,1247) | 97.56 (86.74,109.14) | -0.72 (-0.77 - -0.66) | 12 (5,21) | 1.29 (0.49,2.51) | 0.72 (-0.96 - 2.43) | 2066 (1374,3283) | 121.54 (52.71,225.93) | 0.49 (-1.00 - 2.00) |
| Lithuania | Both | 32 (16,56) | 6 (2.46,11.4) | -0.66 (-0.78 - -0.55) | 294 (236,364) | 54.94 (43.18,68.59) | -0.46 (-0.48 - -0.44) | 0 (0,0) | 0.01 (0,0.01) | -7.06 (-8.43 - -5.67) | 20 (13,27) | 10.66 (6.45,16.14) | -1.34 (-1.63 - -1.05) |
|  | Female | 20 (11,36) | 7.8 (3.21,14.58) | -0.57 (-0.66 - -0.48) | 186 (150,230) | 71.41 (56.12,89.31) | -0.41 (-0.45 - -0.37) | 0 (0,0) | 0 (0,0.01) | -8.30 (-9.33 - -7.26) | 37 (23,56) | 14.27 (7.52,23.14) | -1.21 (-1.51 - -0.90) |
|  | Male | 12 (6,21) | 4.29 (1.44,8.61) | -0.81 (-1.00 - -0.63) | 108 (85,134) | 39.28 (30.31,49.39) | -0.53 (-0.56 - -0.50) | 0 (0,0) | 0.01 (0,0.01) | -6.49 (-8.34 - -4.60) | 57 (37,81) | 7.22 (4.65,10.42) | -1.56 (-2.04 - -1.08) |
| Luxembourg | Both | 5 (2,9) | 3.93 (1.34,7.88) | -0.72 (-0.86 - -0.58) | 55 (48,64) | 40.44 (34.15,47.03) | -0.49 (-0.50 - -0.47) | 0 (0,0) | 0.01 (0.01,0.01) | -9.36 (-10.12 - -8.59) | 4 (3,5) | 5.96 (4.25,8.16) | -3.83 (-4.17 - -3.49) |
|  | Female | 3 (1,5) | 4.17 (1.42,8.3) | -0.59 (-0.70 - -0.48) | 29 (25,34) | 44.64 (37.26,52.42) | -0.44 (-0.46 - -0.42) | 0 (0,0) | 0.01 (0.01,0.01) | -8.91 (-9.84 - -7.97) | 5 (3,6) | 6.95 (4.63,9.94) | -3.28 (-3.56 - -3.00) |
|  | Male | 3 (1,5) | 3.71 (1.17,7.6) | -0.83 (-0.90 - -0.77) | 26 (22,30) | 36.53 (30.5,43.02) | -0.53 (-0.55 - -0.50) | 0 (0,0) | 0.01 (0.01,0.01) | -9.82 (-13.03 - -6.50) | 8 (6,11) | 5.04 (3.67,6.75) | -4.48 (-4.92 - -4.05) |
| Madagascar | Both | 1149 (665,1910) | 7.77 (3.67,14) | -0.19 (-0.32 - -0.05) | 10063 (8931,11291) | 70.35 (61.29,79.85) | -0.25 (-0.26 - -0.24) | 29 (20,42) | 0.2 (0.12,0.33) | -1.74 (-2.06 - -1.40) | 1733 (1173,2478) | 25.15 (16.8,35.99) | -1.35 (-1.61 - -1.08) |
|  | Female | 623 (360,1041) | 8.37 (3.93,15.12) | -0.19 (-0.32 - -0.06) | 5822 (5099,6591) | 80.65 (70.04,92.47) | -0.24 (-0.25 - -0.23) | 14 (8,23) | 0.2 (0.09,0.36) | -1.44 (-1.69 - -1.18) | 1906 (1308,2643) | 26.46 (15.71,42.22) | -1.07 (-1.31 - -0.84) |
|  | Male | 526 (293,898) | 7.19 (3.26,13.19) | -0.20 (-0.27 - -0.12) | 4241 (3708,4795) | 59.96 (51.58,68.53) | -0.27 (-0.28 - -0.27) | 15 (9,25) | 0.21 (0.1,0.38) | -1.99 (-2.39 - -1.59) | 3639 (2756,4656) | 23.76 (14.02,38.58) | -1.63 (-1.92 - -1.33) |
| Malawi | Both | 815 (454,1386) | 7.71 (3.63,13.93) | -0.17 (-0.31 - -0.04) | 7396 (6527,8352) | 70.51 (61.41,80.64) | -0.35 (-0.36 - -0.34) | 13 (8,19) | 0.12 (0.07,0.2) | -3.21 (-3.57 - -2.84) | 846 (532,1239) | 18.79 (12.8,27.2) | -2.33 (-2.57 - -2.09) |
|  | Female | 447 (250,772) | 8.41 (3.87,15.25) | -0.19 (-0.31 - -0.06) | 4280 (3773,4842) | 80.65 (70.2,92.34) | -0.33 (-0.35 - -0.31) | 7 (4,11) | 0.13 (0.06,0.23) | -3.32 (-3.83 - -2.80) | 1119 (804,1509) | 21.22 (12.3,33.24) | -2.31 (-2.60 - -2.01) |
|  | Male | 367 (201,622) | 7.02 (3.19,12.84) | -0.18 (-0.21 - -0.14) | 3117 (2703,3575) | 60.17 (51.61,69.71) | -0.39 (-0.40 - -0.38) | 6 (3,10) | 0.11 (0.04,0.22) | -3.07 (-3.44 - -2.70) | 1965 (1467,2597) | 16.31 (9.17,26.22) | -2.35 (-2.63 - -2.08) |
| Malaysia | Both | 692 (380,1216) | 6.73 (2.91,12.54) | 0.00 (-0.07 - 0.07) | 6612 (5748,7505) | 62.97 (54.35,72.08) | 0.16 (0.14 - 0.19) | 4 (3,6) | 0.04 (0.03,0.06) | -2.92 (-4.00 - -1.84) | 690 (487,913) | 15.14 (9.98,21.2) | -0.73 (-1.02 - -0.44) |
|  | Female | 391 (223,685) | 7.83 (3.44,14.62) | 0.10 (0.06 - 0.14) | 3735 (3257,4265) | 73.14 (63.01,83.94) | 0.26 (0.23 - 0.29) | 2 (1,3) | 0.04 (0.02,0.06) | -3.15 (-4.07 - -2.23) | 900 (582,1231) | 17.64 (9.69,26.88) | -0.46 (-0.58 - -0.34) |
|  | Male | 301 (158,527) | 5.69 (2.26,10.81) | -0.12 (-0.21 - -0.03) | 2878 (2485,3293) | 53.35 (45.71,61.81) | 0.06 (0.03 - 0.09) | 3 (2,4) | 0.05 (0.03,0.08) | -2.84 (-3.97 - -1.69) | 1590 (1125,2070) | 12.78 (8.66,18.36) | -0.96 (-1.35 - -0.57) |
| Maldives | Both | 7 (3,12) | 5.05 (2.07,9.63) | -0.96 (-1.03 - -0.89) | 61 (53,69) | 47.25 (40.49,54.25) | -0.97 (-1.00 - -0.94) | 0 (0,0) | 0.05 (0.03,0.07) | -5.29 (-5.90 - -4.67) | 8 (6,10) | 12.46 (8.52,17.06) | -2.98 (-3.27 - -2.68) |
|  | Female | 4 (2,7) | 5.66 (2.22,11.25) | -0.96 (-1.06 - -0.86) | 32 (28,37) | 52.44 (44.51,60.45) | -0.87 (-0.89 - -0.85) | 0 (0,0) | 0.03 (0.02,0.05) | -6.61 (-6.95 - -6.26) | 8 (5,11) | 13.06 (7.45,19.97) | -3.23 (-3.37 - -3.09) |
|  | Male | 3 (2,5) | 4.49 (1.77,8.55) | -0.93 (-1.08 - -0.77) | 29 (25,33) | 42.53 (36.4,49.02) | -1.05 (-1.08 - -1.02) | 0 (0,0) | 0.06 (0.03,0.1) | -4.19 (-5.24 - -3.12) | 16 (12,21) | 11.91 (8.04,16.48) | -2.68 (-3.28 - -2.09) |
| Mali | Both | 1398 (894,2235) | 9.53 (5,16.01) | -0.37 (-0.39 - -0.35) | 12103 (10940,13352) | 93.64 (83.34,104.34) | -0.27 (-0.28 - -0.27) | 51 (30,84) | 0.32 (0.16,0.57) | -1.43 (-1.98 - -0.87) | 3600 (2216,5933) | 39.15 (23.69,62.47) | -1.16 (-1.58 - -0.73) |
|  | Female | 836 (552,1288) | 11.04 (5.96,18.45) | -0.28 (-0.35 - -0.20) | 7981 (7202,8828) | 122.61 (109.51,136.7) | -0.19 (-0.20 - -0.18) | 15 (7,27) | 0.21 (0.08,0.42) | -1.02 (-1.33 - -0.71) | 2325 (1471,3449) | 33.58 (18.15,57.02) | -0.69 (-0.88 - -0.51) |
|  | Male | 561 (331,912) | 8.09 (3.88,14.24) | -0.51 (-0.53 - -0.49) | 4123 (3655,4626) | 65.28 (56.84,74.22) | -0.42 (-0.44 - -0.40) | 36 (20,62) | 0.43 (0.21,0.81) | -1.61 (-2.30 - -0.90) | 5925 (4142,8637) | 44.41 (24.31,78.72) | -1.46 (-2.06 - -0.86) |
| Malta | Both | 3 (1,6) | 3.84 (1.3,7.7) | -0.73 (-0.82 - -0.65) | 33 (28,38) | 39.38 (32.97,45.96) | -0.46 (-0.49 - -0.44) | 0 (0,0) | 0.02 (0.02,0.03) | -5.51 (-7.04 - -3.96) | 3 (2,3) | 6.96 (5.23,9.01) | -2.77 (-3.32 - -2.21) |
|  | Female | 2 (1,3) | 4.13 (1.44,8.15) | -0.64 (-0.71 - -0.56) | 18 (15,20) | 43.89 (36.71,51.58) | -0.42 (-0.44 - -0.39) | 0 (0,0) | 0.02 (0.01,0.02) | -5.60 (-7.05 - -4.12) | 3 (2,4) | 7.6 (5.35,10.26) | -2.59 (-3.08 - -2.09) |
|  | Male | 2 (1,3) | 3.57 (1.1,7.37) | -0.83 (-0.89 - -0.76) | 15 (13,18) | 35.15 (29,41.52) | -0.52 (-0.54 - -0.50) | 0 (0,0) | 0.03 (0.02,0.04) | -5.10 (-6.77 - -3.40) | 6 (5,7) | 6.36 (4.83,8.2) | -3.05 (-3.47 - -2.63) |
| Marshall Islands | Both | 2 (1,3) | 7.78 (3.73,13.63) | -0.36 (-0.41 - -0.32) | 19 (17,22) | 83.88 (73.81,94.26) | -0.49 (-0.52 - -0.47) | 0 (0,0) | 0.18 (0.1,0.3) | 0.14 (-0.15 - 0.43) | 3 (2,4) | 30.53 (20.2,43.44) | -0.27 (-0.39 - -0.15) |
|  | Female | 1 (1,2) | 9.08 (4.35,16.06) | -0.22 (-0.29 - -0.16) | 11 (10,12) | 96.3 (84.13,108.81) | -0.44 (-0.47 - -0.42) | 0 (0,0) | 0.2 (0.1,0.38) | 0.15 (-0.13 - 0.43) | 4 (3,5) | 35.67 (21.84,54.9) | -0.20 (-0.34 - -0.06) |
|  | Male | 1 (0,1) | 6.53 (2.9,11.68) | -0.54 (-0.60 - -0.49) | 9 (7,9) | 71.88 (62.7,81.42) | -0.56 (-0.60 - -0.52) | 0 (0,0) | 0.16 (0.07,0.3) | 0.02 (-0.40 - 0.43) | 7 (5,9) | 25.51 (14.88,40.6) | -0.29 (-0.50 - -0.07) |
| Mauritania | Both | 229 (137,373) | 9.82 (4.93,17.31) | -0.83 (-0.88 - -0.79) | 2146 (1918,2380) | 97.05 (85.67,108.79) | -0.72 (-0.74 - -0.69) | 5 (3,6) | 0.19 (0.11,0.3) | -2.48 (-2.69 - -2.26) | 359 (246,494) | 28.4 (18.86,40.33) | -1.86 (-2.02 - -1.71) |
|  | Female | 132 (81,213) | 11.19 (5.67,19.09) | -0.72 (-0.78 - -0.66) | 1411 (1270,1561) | 126.14 (112.11,141.07) | -0.72 (-0.74 - -0.70) | 1 (1,2) | 0.12 (0.05,0.22) | -1.89 (-2.41 - -1.36) | 297 (208,399) | 26.5 (15.27,41.36) | -1.19 (-1.28 - -1.11) |
|  | Male | 97 (54,163) | 8.52 (3.84,15.82) | -0.96 (-0.99 - -0.94) | 735 (641,833) | 67.99 (58.23,78.31) | -0.72 (-0.74 - -0.71) | 3 (2,5) | 0.26 (0.14,0.44) | -2.73 (-2.97 - -2.49) | 656 (508,836) | 30.05 (18.17,45.69) | -2.37 (-2.57 - -2.17) |
| Mauritius | Both | 17 (9,29) | 5.51 (2.23,10.3) | -0.80 (-0.91 - -0.70) | 166 (145,189) | 51.63 (44.35,59.51) | -0.84 (-0.86 - -0.82) | 0 (0,0) | 0.09 (0.07,0.1) | -0.84 (-2.81 - 1.18) | 23 (18,29) | 16.22 (12.23,21) | -1.14 (-2.21 - -0.06) |
|  | Female | 10 (5,17) | 6.45 (2.68,12.26) | -0.71 (-0.78 - -0.63) | 95 (82,109) | 59.33 (50.62,69.19) | -0.70 (-0.72 - -0.69) | 0 (0,0) | 0.09 (0.07,0.11) | -0.95 (-3.27 - 1.43) | 30 (22,39) | 18.53 (12.63,25.68) | -0.98 (-1.82 - -0.14) |
|  | Male | 7 (4,12) | 4.58 (1.75,8.91) | -0.94 (-1.05 - -0.83) | 72 (62,82) | 44.09 (37.56,50.94) | -1.01 (-1.04 - -0.97) | 0 (0,0) | 0.08 (0.06,0.11) | -1.07 (-3.59 - 1.52) | 53 (41,66) | 13.95 (10.66,17.92) | -1.19 (-2.21 - -0.15) |
| Mexico | Both | 2575 (1356,4517) | 5.95 (2.48,11.18) | -1.08 (-1.22 - -0.94) | 25658 (20566,31268) | 57.57 (45.19,71.24) | -0.93 (-0.96 - -0.90) | 23 (20,27) | 0.05 (0.05,0.06) | -4.11 (-5.00 - -3.21) | 1794 (1494,2133) | 9.2 (7.24,11.55) | -2.98 (-3.52 - -2.45) |
|  | Female | 1517 (828,2693) | 7.09 (3.03,13.25) | -1.11 (-1.25 - -0.97) | 14888 (11893,18143) | 67.51 (52.96,83.46) | -0.92 (-0.94 - -0.89) | 11 (9,13) | 0.05 (0.04,0.06) | -4.22 (-5.60 - -2.81) | 2222 (1765,2711) | 10.25 (7.9,13.04) | -2.82 (-3.27 - -2.38) |
|  | Male | 1058 (513,1842) | 4.85 (1.88,9.41) | -1.02 (-1.17 - -0.87) | 10770 (8672,13227) | 47.83 (37.48,59.44) | -0.93 (-0.97 - -0.90) | 13 (11,15) | 0.06 (0.05,0.07) | -4.05 (-4.85 - -3.25) | 4016 (3296,4839) | 8.17 (6.51,10.17) | -3.21 (-3.76 - -2.65) |
| Micronesia (Federated States of) | Both | 3 (2,5) | 8.07 (3.93,14.33) | -0.56 (-0.62 - -0.51) | 36 (32,40) | 84.32 (73.99,95.36) | -0.72 (-0.75 - -0.69) | 0 (0,0) | 0.14 (0.07,0.24) | -2.60 (-2.92 - -2.28) | 5 (3,7) | 26.95 (17.45,38.86) | -1.74 (-1.92 - -1.54) |
|  | Female | 2 (1,3) | 9.29 (4.47,16.66) | -0.59 (-0.62 - -0.56) | 20 (18,22) | 95.52 (83.31,108.58) | -0.76 (-0.80 - -0.72) | 0 (0,0) | 0.16 (0.07,0.31) | -2.44 (-2.80 - -2.08) | 7 (5,9) | 31.98 (18.76,48.92) | -1.65 (-1.78 - -1.52) |
|  | Male | 1 (1,2) | 6.93 (3.27,12.38) | -0.53 (-0.62 - -0.43) | 16 (14,18) | 73.76 (64.2,84.15) | -0.68 (-0.69 - -0.66) | 0 (0,0) | 0.12 (0.05,0.24) | -2.85 (-3.20 - -2.50) | 11 (8,15) | 22.19 (12.55,35.39) | -1.84 (-1.95 - -1.73) |
| Monaco | Both | 0 (0,1) | 4.53 (1.63,8.91) | -0.22 (-0.29 - -0.16) | 3 (3,4) | 46.62 (39.47,54.17) | -0.10 (-0.13 - -0.07) | 0 (0,0) | 0.04 (0.02,0.05) | -3.92 (-4.30 - -3.54) | 0 (0,0) | 8.94 (6.56,11.7) | -1.98 (-2.18 - -1.77) |
|  | Female | 0 (0,0) | 4.9 (1.8,9.55) | -0.19 (-0.23 - -0.15) | 2 (2,2) | 51.95 (43.81,60.72) | -0.08 (-0.10 - -0.05) | 0 (0,0) | 0.05 (0.03,0.08) | -3.73 (-4.09 - -3.37) | 0 (0,0) | 11.3 (7.82,15.56) | -2.01 (-2.20 - -1.82) |
|  | Male | 0 (0,0) | 4.16 (1.38,8.43) | -0.26 (-0.35 - -0.18) | 1 (1,2) | 41.47 (34.59,48.43) | -0.13 (-0.14 - -0.13) | 0 (0,0) | 0.02 (0.01,0.04) | -4.36 (-4.80 - -3.91) | 1 (0,1) | 6.66 (4.74,8.95) | -1.89 (-2.02 - -1.77) |
| Mongolia | Both | 110 (64,182) | 8.64 (4.35,15.08) | -0.58 (-0.62 - -0.54) | 935 (835,1031) | 79.42 (70.02,89) | -0.96 (-1.01 - -0.90) | 0 (0,0) | 0.01 (0.01,0.02) | -2.42 (-5.39 - 0.63) | 64 (44,88) | 15.76 (9.79,22.96) | -1.03 (-1.22 - -0.84) |
|  | Female | 70 (43,114) | 11.16 (5.75,19) | -0.61 (-0.76 - -0.46) | 602 (538,667) | 104.23 (92.07,116.59) | -0.96 (-1.05 - -0.87) | 0 (0,0) | 0.01 (0.01,0.02) | -3.19 (-6.39 - 0.12) | 121 (79,172) | 20.96 (10.91,33.14) | -1.05 (-1.17 - -0.94) |
|  | Male | 40 (22,71) | 6.23 (2.72,11.57) | -0.44 (-0.56 - -0.33) | 333 (294,374) | 55.61 (48.18,63.5) | -0.87 (-0.92 - -0.81) | 0 (0,0) | 0.02 (0.01,0.03) | -1.82 (-5.13 - 1.60) | 185 (126,260) | 10.78 (6.87,15.87) | -1.09 (-1.31 - -0.86) |
| Montenegro | Both | 8 (4,14) | 5.21 (2,10.13) | -0.06 (-0.25 - 0.14) | 72 (62,82) | 46.75 (39.96,53.87) | -0.02 (-0.04 - -0.01) | 0 (0,0) | 0.01 (0.01,0.02) | -2.40 (-4.32 - -0.44) | 5 (4,8) | 9.48 (5.98,13.39) | -0.40 (-0.78 - -0.03) |
|  | Female | 5 (3,9) | 6.63 (2.63,12.64) | 0.05 (-0.07 - 0.16) | 44 (38,51) | 60.01 (51.18,69.24) | 0.05 (0.04 - 0.06) | 0 (0,0) | 0.01 (0,0.02) | -3.39 (-6.52 - -0.16) | 9 (6,13) | 12.4 (6.98,19.18) | -0.33 (-0.66 - -0.01) |
|  | Male | 3 (1,6) | 3.9 (1.27,7.98) | -0.19 (-0.35 - -0.04) | 28 (24,32) | 34.54 (29.1,40.57) | -0.09 (-0.11 - -0.07) | 0 (0,0) | 0.01 (0.01,0.02) | -1.64 (-5.13 - 1.97) | 15 (10,20) | 6.79 (4.5,9.71) | -0.41 (-0.98 - 0.17) |
| Morocco | Both | 1298 (795,2111) | 10.06 (5.03,17.54) | -0.20 (-0.28 - -0.11) | 11716 (10430,13146) | 90.67 (79.86,102.79) | -0.41 (-0.43 - -0.39) | 33 (18,55) | 0.26 (0.13,0.48) | -4.53 (-4.98 - -4.08) | 1690 (1070,2417) | 33.26 (20.54,52.15) | -3.74 (-4.12 - -3.36) |
|  | Female | 706 (431,1160) | 11.21 (5.51,19.51) | -0.17 (-0.24 - -0.10) | 6188 (5471,6922) | 98.05 (85.66,111.53) | -0.38 (-0.39 - -0.37) | 21 (10,40) | 0.34 (0.14,0.71) | -4.34 (-4.81 - -3.88) | 2588 (1629,4342) | 41.17 (22.39,74.02) | -3.60 (-3.79 - -3.42) |
|  | Male | 593 (359,957) | 8.98 (4.39,15.83) | -0.21 (-0.29 - -0.14) | 5528 (4897,6206) | 83.61 (73.13,94.94) | -0.42 (-0.44 - -0.39) | 12 (6,20) | 0.19 (0.08,0.36) | -4.80 (-5.29 - -4.31) | 4278 (2898,6231) | 25.69 (14.69,41.62) | -3.85 (-4.28 - -3.43) |
| Mozambique | Both | 1445 (837,2370) | 8.21 (3.91,14.57) | -0.00 (-0.20 - 0.19) | 12222 (10756,13729) | 74.41 (64.72,84.64) | -0.13 (-0.14 - -0.12) | 18 (10,27) | 0.11 (0.06,0.18) | -2.57 (-2.98 - -2.15) | 1582 (1004,2195) | 17.95 (11.95,25.73) | -1.68 (-1.94 - -1.42) |
|  | Female | 791 (453,1292) | 8.79 (4.07,15.84) | -0.05 (-0.23 - 0.13) | 7086 (6214,7957) | 84.2 (73.24,96.12) | -0.13 (-0.14 - -0.12) | 6 (4,10) | 0.07 (0.03,0.13) | -2.61 (-2.98 - -2.23) | 1423 (1027,1909) | 16.72 (10.03,25.74) | -1.28 (-1.49 - -1.08) |
|  | Male | 654 (380,1099) | 7.66 (3.53,13.78) | 0.08 (0.00 - 0.15) | 5136 (4496,5792) | 64.33 (55.14,73.55) | -0.13 (-0.14 - -0.12) | 12 (5,20) | 0.15 (0.06,0.27) | -2.52 (-2.95 - -2.08) | 3005 (2179,3922) | 19.24 (10.98,30.4) | -1.98 (-2.26 - -1.70) |
| Myanmar | Both | 1265 (731,2150) | 6.09 (2.85,11.05) | -0.46 (-0.52 - -0.41) | 11617 (10309,13000) | 55.58 (48.6,62.82) | -0.49 (-0.51 - -0.46) | 49 (30,68) | 0.23 (0.13,0.38) | -3.28 (-3.60 - -2.96) | 3144 (2138,4199) | 29.77 (19.24,43.82) | -2.62 (-2.88 - -2.36) |
|  | Female | 717 (400,1255) | 6.98 (3.18,12.81) | -0.45 (-0.52 - -0.37) | 6629 (5833,7493) | 63.95 (55.85,72.8) | -0.43 (-0.45 - -0.40) | 21 (13,31) | 0.2 (0.11,0.35) | -3.85 (-4.25 - -3.45) | 3037 (2251,4085) | 29.59 (18.53,45.08) | -2.84 (-3.14 - -2.54) |
|  | Male | 548 (303,896) | 5.21 (2.24,9.56) | -0.47 (-0.51 - -0.44) | 4989 (4380,5614) | 47.34 (41.04,53.88) | -0.54 (-0.56 - -0.51) | 28 (16,41) | 0.27 (0.12,0.46) | -2.76 (-3.03 - -2.48) | 6181 (4538,8046) | 29.93 (17.24,45.53) | -2.39 (-2.62 - -2.16) |
| Namibia | Both | 66 (35,114) | 6.14 (2.56,11.48) | -0.64 (-0.79 - -0.49) | 628 (540,714) | 59.57 (50.87,68.31) | -0.55 (-0.57 - -0.52) | 1 (1,1) | 0.08 (0.04,0.13) | -1.41 (-1.85 - -0.96) | 61 (46,84) | 13.91 (9.23,19.97) | -1.00 (-1.23 - -0.77) |
|  | Female | 42 (23,72) | 7.82 (3.39,14.61) | -0.66 (-0.72 - -0.60) | 413 (357,470) | 77.97 (66.45,89.6) | -0.60 (-0.61 - -0.58) | 0 (0,1) | 0.07 (0.03,0.13) | -1.48 (-2.02 - -0.93) | 86 (59,119) | 16.21 (9.39,25.77) | -0.94 (-1.18 - -0.71) |
|  | Male | 24 (11,42) | 4.47 (1.55,8.62) | -0.57 (-0.69 - -0.45) | 215 (183,248) | 41.02 (34.62,47.95) | -0.43 (-0.46 - -0.41) | 0 (0,1) | 0.08 (0.04,0.16) | -1.38 (-1.74 - -1.02) | 147 (109,192) | 11.59 (7.69,17.62) | -1.07 (-1.29 - -0.85) |
| Nauru | Both | 1 (0,1) | 13.95 (8.2,22.24) | 0.21 (0.14 - 0.28) | 7 (7,8) | 148.04 (134.19,163.13) | -0.15 (-0.17 - -0.12) | 0 (0,0) | 0.67 (0.37,1.14) | -0.08 (-0.29 - 0.14) | 2 (1,3) | 82.44 (53.46,127.22) | -0.14 (-0.33 - 0.06) |
|  | Female | 0 (0,1) | 16.4 (9.52,25.86) | 0.53 (0.48 - 0.58) | 4 (4,5) | 171.55 (154.15,190.35) | 0.12 (0.12 - 0.12) | 0 (0,0) | 0.7 (0.36,1.31) | 0.27 (0.06 - 0.49) | 2 (2,3) | 90.92 (55.22,148.08) | 0.17 (-0.00 - 0.34) |
|  | Male | 0 (0,0) | 11.7 (6.73,18.65) | -0.14 (-0.25 - -0.04) | 3 (3,4) | 126.15 (113.31,139.62) | -0.44 (-0.47 - -0.41) | 0 (0,0) | 0.63 (0.29,1.24) | -0.39 (-0.63 - -0.16) | 4 (3,6) | 74.39 (40.3,129.64) | -0.43 (-0.67 - -0.20) |
| Nepal | Both | 500 (224,911) | 3.99 (1.39,8.15) | -0.34 (-0.58 - -0.10) | 4358 (3693,5001) | 34.3 (28.61,40.03) | -0.41 (-0.42 - -0.39) | 6 (4,10) | 0.05 (0.02,0.09) | -5.01 (-5.33 - -4.69) | 520 (363,826) | 8.68 (5.85,12.52) | -3.36 (-3.54 - -3.19) |
|  | Female | 290 (132,533) | 4.7 (1.57,9.58) | -0.26 (-0.49 - -0.03) | 2561 (2156,2938) | 40.62 (33.83,47.6) | -0.33 (-0.34 - -0.31) | 2 (1,4) | 0.04 (0.01,0.07) | -6.07 (-6.37 - -5.77) | 573 (411,754) | 9.15 (5.86,13.1) | -3.58 (-3.68 - -3.47) |
|  | Male | 210 (86,396) | 3.31 (0.98,6.97) | -0.43 (-0.60 - -0.25) | 1797 (1513,2086) | 28.09 (23.11,33.26) | -0.51 (-0.54 - -0.48) | 4 (2,7) | 0.06 (0.02,0.12) | -4.02 (-4.26 - -3.78) | 1093 (821,1511) | 8.18 (4.95,13.95) | -3.08 (-3.25 - -2.91) |
| Netherlands | Both | 180 (84,328) | 4.88 (1.9,9.65) | -0.78 (-0.92 - -0.64) | 1945 (1674,2216) | 50.59 (42.63,58.72) | -0.66 (-0.68 - -0.64) | 0 (0,0) | 0.01 (0,0.01) | -7.52 (-7.88 - -7.15) | 113 (81,149) | 7.11 (5.01,9.63) | -2.13 (-2.27 - -1.99) |
|  | Female | 96 (46,176) | 5.34 (2.05,10.52) | -0.72 (-0.84 - -0.59) | 1065 (918,1225) | 56.82 (47.96,66.25) | -0.60 (-0.61 - -0.58) | 0 (0,0) | 0.01 (0.01,0.01) | -7.11 (-7.50 - -6.71) | 159 (113,220) | 8.53 (5.73,11.96) | -1.87 (-2.00 - -1.75) |
|  | Male | 84 (37,148) | 4.45 (1.58,8.92) | -0.85 (-1.00 - -0.70) | 880 (748,1014) | 44.65 (37.16,52.02) | -0.74 (-0.76 - -0.71) | 0 (0,0) | 0.01 (0,0.01) | -7.88 (-8.57 - -7.17) | 272 (197,365) | 5.75 (4.07,7.69) | -2.40 (-2.53 - -2.27) |
| New Zealand | Both | 73 (35,128) | 5.57 (2.26,10.8) | -0.44 (-0.47 - -0.41) | 701 (560,869) | 52.99 (41.33,66.68) | -0.29 (-0.30 - -0.28) | 0 (0,0) | 0.01 (0.01,0.02) | -6.26 (-8.07 - -4.41) | 47 (34,63) | 8.44 (5.82,11.69) | -2.07 (-2.43 - -1.72) |
|  | Female | 39 (19,70) | 6.22 (2.54,12.1) | -0.19 (-0.24 - -0.15) | 387 (305,480) | 60.29 (46.69,76.15) | -0.04 (-0.09 - 0.00) | 0 (0,0) | 0.01 (0.01,0.01) | -5.83 (-6.41 - -5.25) | 64 (43,89) | 10.02 (6.43,14.53) | -1.21 (-1.33 - -1.09) |
|  | Male | 33 (16,59) | 4.95 (1.92,9.79) | -0.71 (-0.77 - -0.65) | 315 (251,387) | 46.12 (36.08,57.81) | -0.58 (-0.61 - -0.55) | 0 (0,0) | 0.01 (0.01,0.02) | -6.67 (-8.52 - -4.78) | 111 (77,152) | 6.96 (4.91,9.44) | -2.95 (-3.49 - -2.40) |
| Nicaragua | Both | 126 (61,228) | 4.83 (1.81,9.45) | -0.60 (-0.76 - -0.44) | 1208 (1042,1384) | 46.57 (39.56,53.85) | -0.52 (-0.54 - -0.51) | 1 (1,2) | 0.04 (0.03,0.06) | -5.22 (-5.76 - -4.69) | 82 (63,107) | 7.25 (5.26,9.79) | -3.68 (-3.96 - -3.40) |
|  | Female | 72 (35,128) | 5.67 (2.13,10.93) | -0.62 (-0.79 - -0.46) | 680 (585,786) | 53.82 (45.61,62.6) | -0.53 (-0.54 - -0.52) | 1 (0,1) | 0.04 (0.02,0.07) | -4.96 (-5.49 - -4.44) | 105 (76,136) | 8.33 (5.69,11.62) | -3.27 (-3.70 - -2.84) |
|  | Male | 54 (23,102) | 4.04 (1.33,8.37) | -0.50 (-0.60 - -0.40) | 528 (449,603) | 39.67 (33.32,46.01) | -0.48 (-0.50 - -0.46) | 1 (0,1) | 0.04 (0.02,0.07) | -5.54 (-5.97 - -5.10) | 187 (143,238) | 6.21 (4.33,8.72) | -4.11 (-4.47 - -3.75) |
| Niger | Both | 1487 (934,2379) | 9.36 (4.79,16.11) | -0.48 (-0.61 - -0.35) | 12288 (10997,13620) | 88.16 (77.62,98.86) | -0.57 (-0.59 - -0.55) | 39 (18,71) | 0.22 (0.09,0.43) | -3.33 (-3.89 - -2.76) | 2829 (1425,5440) | 29.59 (16.9,49.33) | -2.61 (-3.00 - -2.22) |
|  | Female | 869 (563,1349) | 10.56 (5.51,17.79) | -0.44 (-0.56 - -0.31) | 7868 (7067,8655) | 111.03 (98.5,123.81) | -0.53 (-0.56 - -0.51) | 12 (4,21) | 0.14 (0.05,0.29) | -2.65 (-3.21 - -2.09) | 2054 (1290,2953) | 26.81 (13.7,44.41) | -1.74 (-2.03 - -1.44) |
|  | Male | 618 (367,1028) | 8.24 (3.9,14.58) | -0.58 (-0.61 - -0.55) | 4419 (3870,5011) | 65.47 (56.32,75.53) | -0.60 (-0.63 - -0.58) | 27 (10,56) | 0.29 (0.1,0.63) | -3.58 (-4.21 - -2.96) | 4883 (3137,7652) | 32.08 (14.71,62.63) | -3.14 (-3.65 - -2.62) |
| Nigeria | Both | 13256 (7994,21394) | 10.31 (5.18,17.78) | -0.20 (-0.37 - -0.03) | 119306 (97534,144913) | 99.23 (79.57,121.17) | -0.18 (-0.21 - -0.16) | 587 (231,1004) | 0.42 (0.16,0.74) | -0.21 (-0.37 - -0.04) | 47473 (18788,79885) | 48.88 (25.03,78.04) | -0.21 (-0.34 - -0.08) |
|  | Female | 7917 (4807,12692) | 11.94 (5.9,20.46) | 0.06 (-0.13 - 0.25) | 78479 (64070,94574) | 126.02 (101.28,153.99) | 0.06 (0.04 - 0.07) | 93 (51,217) | 0.14 (0.07,0.32) | -1.06 (-1.25 - -0.88) | 18470 (13480,29009) | 28.51 (19.16,47.59) | -0.45 (-0.56 - -0.35) |
|  | Male | 5338 (3125,8869) | 8.73 (4.16,15.35) | -0.52 (-0.65 - -0.39) | 40827 (32870,50405) | 71.16 (55.85,89.07) | -0.54 (-0.57 - -0.50) | 495 (161,869) | 0.7 (0.23,1.27) | -0.07 (-0.24 - 0.10) | 65943 (35196,102602) | 68.84 (27.31,118.19) | -0.15 (-0.30 - 0.00) |
| Niue | Both | 0 (0,0) | 11.5 (6.2,18.61) | 0.27 (0.22 - 0.31) | 1 (1,1) | 133 (119.67,145.9) | 0.11 (0.10 - 0.12) | 0 (0,0) | 1.15 (0.74,1.84) | 4.56 (3.10 - 6.05) | 0 (0,1) | 121.3 (83.15,182.68) | 3.10 (2.11 - 4.09) |
|  | Female | 0 (0,0) | 12.08 (6.51,19.91) | 0.13 (0.11 - 0.14) | 0 (0,0) | 139.51 (124.57,154.34) | 0.01 (0.01 - 0.02) | 0 (0,0) | 0.98 (0.56,1.62) | 4.85 (3.00 - 6.73) | 0 (0,0) | 108.67 (67.14,164.79) | 3.11 (2.96 - 3.25) |
|  | Male | 0 (0,0) | 11.02 (5.77,17.92) | 0.43 (0.38 - 0.49) | 0 (0,0) | 127.35 (113.62,141.4) | 0.21 (0.19 - 0.23) | 0 (0,0) | 1.32 (0.73,2.41) | 4.38 (3.14 - 5.64) | 1 (0,1) | 134.93 (78.22,232.98) | 3.29 (2.35 - 4.24) |
| North Macedonia | Both | 26 (14,44) | 5.67 (2.41,10.42) | -0.72 (-0.77 - -0.68) | 256 (209,312) | 54.92 (43.68,67.53) | -0.59 (-0.63 - -0.55) | 0 (0,0) | 0.05 (0.03,0.08) | -4.99 (-6.38 - -3.58) | 26 (19,36) | 13.68 (9.26,19.54) | -2.58 (-3.14 - -2.01) |
|  | Female | 16 (9,26) | 7.1 (3.13,13.13) | -0.80 (-0.86 - -0.74) | 157 (127,191) | 69.74 (55.67,85.67) | -0.64 (-0.68 - -0.59) | 0 (0,0) | 0.04 (0.02,0.07) | -5.23 (-6.81 - -3.62) | 38 (25,55) | 16.66 (10.02,25.88) | -2.64 (-3.23 - -2.04) |
|  | Male | 10 (5,17) | 4.33 (1.63,8.4) | -0.62 (-0.69 - -0.55) | 99 (80,124) | 41.06 (32.25,52.04) | -0.47 (-0.49 - -0.45) | 0 (0,0) | 0.05 (0.03,0.09) | -4.79 (-6.11 - -3.45) | 64 (46,88) | 10.9 (7.22,15.63) | -2.54 (-2.96 - -2.12) |
| Northern Mariana Islands | Both | 1 (1,2) | 8.34 (3.84,14.88) | -0.03 (-0.08 - 0.02) | 15 (13,16) | 94.57 (82.98,106.87) | -0.13 (-0.18 - -0.08) | 0 (0,0) | 0.04 (0.03,0.06) | -1.94 (-4.31 - 0.49) | 1 (1,2) | 20.9 (13.22,30.8) | -0.60 (-1.15 - -0.05) |
|  | Female | 1 (0,1) | 9.36 (4.32,16.72) | -0.01 (-0.05 - 0.04) | 8 (7,9) | 105.07 (92.26,119.3) | -0.08 (-0.12 - -0.03) | 0 (0,0) | 0.04 (0.02,0.07) | -3.29 (-4.34 - -2.23) | 2 (1,2) | 24.09 (12.86,39.34) | -0.53 (-0.70 - -0.36) |
|  | Male | 1 (0,1) | 7.4 (3.24,13.29) | 0.00 (-0.13 - 0.14) | 7 (6,8) | 84.85 (73.95,96.58) | -0.11 (-0.14 - -0.07) | 0 (0,0) | 0.04 (0.02,0.07) | -2.32 (-4.07 - -0.54) | 3 (2,4) | 17.95 (9.66,28.69) | -0.67 (-0.94 - -0.39) |
| Norway | Both | 61 (27,109) | 4.94 (1.83,9.69) | -0.84 (-1.02 - -0.66) | 665 (533,821) | 51.86 (40.68,64.86) | -0.47 (-0.48 - -0.46) | 0 (0,0) | 0 (0,0) | -10.05 (-12.01 - -8.05) | 39 (26,52) | 6.98 (4.75,9.73) | -2.09 (-2.33 - -1.86) |
|  | Female | 31 (14,56) | 5.16 (1.94,10.28) | -0.92 (-1.12 - -0.72) | 348 (278,428) | 55.85 (43.62,69.88) | -0.53 (-0.54 - -0.51) | 0 (0,0) | 0 (0,0) | -9.03 (-11.76 - -6.21) | 51 (35,71) | 8.15 (5.44,11.55) | -1.74 (-2.17 - -1.30) |
|  | Male | 30 (13,54) | 4.73 (1.71,9.37) | -0.77 (-0.95 - -0.59) | 317 (252,393) | 48.08 (37.4,60.39) | -0.41 (-0.42 - -0.40) | 0 (0,0) | 0 (0,0) | -12.06 (-12.43 - -11.69) | 90 (61,122) | 5.88 (3.95,8.17) | -2.60 (-2.83 - -2.37) |
| Oman | Both | 189 (122,279) | 13.3 (7.4,21.1) | 0.62 (0.54 - 0.71) | 1512 (1372,1658) | 112.05 (99.85,124.33) | 0.29 (0.28 - 0.31) | 2 (2,3) | 0.16 (0.11,0.23) | -3.38 (-4.05 - -2.71) | 197 (157,248) | 28.5 (20.8,37.63) | -1.95 (-2.36 - -1.55) |
|  | Female | 92 (58,143) | 13.47 (7.02,22.1) | 0.85 (0.75 - 0.94) | 708 (630,784) | 107.76 (94.74,120.71) | 0.45 (0.43 - 0.46) | 1 (1,1) | 0.16 (0.09,0.25) | -4.00 (-4.57 - -3.42) | 194 (152,242) | 29.05 (19.07,41.32) | -2.45 (-2.81 - -2.10) |
|  | Male | 96 (64,139) | 13.13 (7.63,20.67) | 0.45 (0.29 - 0.61) | 804 (726,886) | 116.17 (103.56,129.65) | 0.17 (0.15 - 0.20) | 1 (1,2) | 0.17 (0.1,0.27) | -2.88 (-4.08 - -1.68) | 391 (323,476) | 27.98 (18.85,38.8) | -1.68 (-2.31 - -1.05) |
| Pakistan | Both | 6518 (3608,11195) | 6.04 (2.71,11.05) | -0.13 (-0.32 - 0.06) | 52876 (42485,64986) | 50.55 (39.63,63.14) | -0.26 (-0.28 - -0.24) | 235 (156,372) | 0.21 (0.13,0.35) | -0.51 (-1.16 - 0.14) | 12163 (8224,16530) | 24.9 (16.65,37.61) | -0.51 (-0.97 - -0.04) |
|  | Female | 3790 (2131,6551) | 7.31 (3.31,13.43) | 0.18 (0.12 - 0.24) | 30447 (24442,37420) | 60.69 (47.5,75.54) | -0.07 (-0.08 - -0.06) | 127 (78,242) | 0.24 (0.13,0.47) | -0.63 (-1.25 - -0.01) | 15207 (10717,24885) | 29.07 (18.32,49.23) | -0.56 (-0.97 - -0.16) |
|  | Male | 2728 (1386,4662) | 4.87 (1.96,9.28) | -0.51 (-0.72 - -0.31) | 22430 (17928,27648) | 41.2 (32.26,51.78) | -0.51 (-0.53 - -0.49) | 108 (65,158) | 0.18 (0.1,0.3) | -0.11 (-0.53 - 0.32) | 27370 (19808,39294) | 21.07 (12.79,31.76) | -0.27 (-0.61 - 0.08) |
| Palau | Both | 0 (0,1) | 9.51 (4.9,16.29) | 0.04 (0.01 - 0.08) | 5 (4,5) | 102.57 (91.02,114.48) | -0.30 (-0.31 - -0.28) | 0 (0,0) | 0.33 (0.2,0.52) | -0.81 (-1.01 - -0.61) | 1 (1,1) | 45.73 (31.01,65.38) | -0.76 (-0.90 - -0.62) |
|  | Female | 0 (0,0) | 10.51 (5.29,18.18) | -0.01 (-0.04 - 0.01) | 3 (2,3) | 114.28 (100.74,128.17) | -0.34 (-0.36 - -0.31) | 0 (0,0) | 0.49 (0.27,0.82) | 0.12 (-0.17 - 0.40) | 1 (1,2) | 60.51 (36.56,94.06) | -0.25 (-0.43 - -0.06) |
|  | Male | 0 (0,0) | 8.57 (4.26,14.75) | 0.10 (0.05 - 0.15) | 2 (2,2) | 91.59 (80.72,103.14) | -0.24 (-0.26 - -0.23) | 0 (0,0) | 0.19 (0.12,0.31) | -2.29 (-2.58 - -1.99) | 2 (2,3) | 32.04 (19.57,48.8) | -1.43 (-1.58 - -1.29) |
| Palestine | Both | 204 (116,342) | 8.51 (3.92,15.28) | -0.46 (-0.57 - -0.35) | 1881 (1656,2125) | 79.5 (69.11,90.32) | -0.47 (-0.49 - -0.46) | 7 (5,9) | 0.29 (0.19,0.41) | -4.31 (-5.21 - -3.41) | 405 (311,522) | 34.86 (25.01,46.17) | -3.64 (-4.29 - -2.98) |
|  | Female | 116 (67,197) | 9.92 (4.49,17.89) | -0.47 (-0.49 - -0.45) | 1042 (920,1166) | 90.33 (78.62,102.82) | -0.50 (-0.52 - -0.48) | 3 (2,4) | 0.28 (0.16,0.44) | -4.63 (-6.11 - -3.12) | 423 (319,531) | 36.59 (23.74,52.98) | -3.85 (-4.82 - -2.87) |
|  | Male | 88 (47,148) | 7.18 (3.07,13.17) | -0.46 (-0.62 - -0.30) | 839 (734,958) | 69.19 (59.66,79.74) | -0.44 (-0.45 - -0.42) | 4 (3,5) | 0.3 (0.18,0.46) | -3.94 (-4.38 - -3.50) | 828 (672,1018) | 33.21 (22.45,47.79) | -3.41 (-3.82 - -3.00) |
| Panama | Both | 73 (35,134) | 4.88 (1.9,9.34) | -0.93 (-1.01 - -0.85) | 764 (668,868) | 50.78 (43.85,58.17) | -0.98 (-1.01 - -0.95) | 1 (1,1) | 0.05 (0.03,0.06) | -3.09 (-4.19 - -1.97) | 53 (43,64) | 7.78 (6.15,9.7) | -2.10 (-2.67 - -1.52) |
|  | Female | 40 (19,74) | 5.42 (2.08,10.55) | -0.47 (-0.50 - -0.44) | 379 (324,436) | 51.86 (43.84,60.39) | -0.45 (-0.47 - -0.44) | 0 (0,0) | 0.05 (0.04,0.07) | -3.23 (-4.52 - -1.92) | 63 (51,78) | 8.7 (6.6,11.36) | -1.97 (-2.90 - -1.04) |
|  | Male | 34 (16,60) | 4.36 (1.67,8.42) | -1.40 (-1.43 - -1.36) | 384 (338,433) | 49.74 (43.16,56.54) | -1.43 (-1.48 - -1.37) | 0 (0,0) | 0.04 (0.03,0.06) | -3.22 (-4.25 - -2.18) | 116 (95,140) | 6.91 (5.38,8.77) | -2.31 (-3.01 - -1.61) |
| Papua New Guinea | Both | 377 (224,593) | 7.26 (3.58,12.63) | -0.24 (-0.32 - -0.17) | 3767 (3386,4141) | 82.02 (72.74,91.43) | -0.20 (-0.20 - -0.19) | 25 (13,49) | 0.44 (0.2,0.91) | -0.56 (-1.00 - -0.12) | 1375 (752,2523) | 53.18 (29.22,98.09) | -0.48 (-0.81 - -0.14) |
|  | Female | 204 (120,326) | 8.31 (3.95,14.81) | -0.21 (-0.27 - -0.14) | 2016 (1793,2238) | 92.9 (81.66,104.34) | -0.14 (-0.15 - -0.14) | 13 (6,26) | 0.48 (0.19,1.06) | -0.94 (-1.30 - -0.57) | 1501 (885,2676) | 59.49 (29.08,114.41) | -0.76 (-0.97 - -0.55) |
|  | Male | 173 (105,265) | 6.31 (3.1,11.04) | -0.27 (-0.38 - -0.16) | 1751 (1571,1939) | 72.25 (63.94,80.89) | -0.25 (-0.26 - -0.23) | 12 (6,25) | 0.4 (0.17,0.87) | -0.01 (-0.78 - 0.76) | 2876 (1766,5037) | 47.53 (22.83,93.23) | -0.07 (-0.69 - 0.55) |
| Paraguay | Both | 102 (48,184) | 3.82 (1.36,7.86) | -0.34 (-0.52 - -0.16) | 1011 (863,1166) | 37.4 (31.62,43.52) | -0.34 (-0.36 - -0.33) | 1 (1,1) | 0.03 (0.02,0.05) | -2.68 (-3.83 - -1.51) | 64 (49,82) | 5.49 (4.11,7.29) | -1.71 (-2.28 - -1.14) |
|  | Female | 56 (26,102) | 4.32 (1.53,8.77) | -0.35 (-0.54 - -0.16) | 557 (475,641) | 42.19 (35.49,49.26) | -0.33 (-0.34 - -0.31) | 0 (0,1) | 0.03 (0.02,0.05) | -2.98 (-4.23 - -1.70) | 84 (64,110) | 6.38 (4.65,8.58) | -1.76 (-2.35 - -1.17) |
|  | Male | 46 (21,84) | 3.35 (1.03,7.22) | -0.33 (-0.48 - -0.18) | 454 (387,522) | 32.82 (27.56,38.22) | -0.35 (-0.38 - -0.34) | 0 (0,1) | 0.03 (0.02,0.05) | -2.48 (-3.68 - -1.26) | 148 (116,190) | 4.63 (3.3,6.48) | -1.70 (-2.33 - -1.07) |
| Peru | Both | 650 (336,1147) | 5.2 (2.08,9.95) | -0.43 (-0.56 - -0.31) | 5667 (4912,6424) | 45.95 (39.29,52.66) | -0.54 (-0.55 - -0.53) | 10 (7,13) | 0.08 (0.05,0.12) | -4.30 (-5.06 - -3.53) | 570 (433,734) | 9.98 (7.02,13.61) | -3.49 (-4.01 - -2.97) |
|  | Female | 350 (184,625) | 5.87 (2.34,11.15) | -0.34 (-0.41 - -0.26) | 3041 (2636,3464) | 52.02 (44.41,60.04) | -0.41 (-0.42 - -0.39) | 5 (3,7) | 0.08 (0.05,0.14) | -3.46 (-4.24 - -2.67) | 669 (497,855) | 11.39 (7.6,16.07) | -2.69 (-3.20 - -2.18) |
|  | Male | 300 (151,524) | 4.6 (1.65,9.19) | -0.52 (-0.69 - -0.35) | 2626 (2269,2997) | 40.48 (34.5,46.95) | -0.67 (-0.70 - -0.64) | 5 (3,6) | 0.07 (0.04,0.12) | -4.79 (-5.64 - -3.92) | 1239 (959,1554) | 8.72 (5.85,12.46) | -4.10 (-4.80 - -3.41) |
| Philippines | Both | 2748 (1518,4640) | 6.14 (2.71,11.18) | 0.02 (-0.06 - 0.10) | 23770 (19215,28988) | 53.04 (41.77,66.11) | -0.32 (-0.35 - -0.30) | 50 (41,61) | 0.11 (0.09,0.15) | -2.60 (-3.10 - -2.11) | 3680 (2915,4545) | 19.11 (14.59,24.38) | -1.69 (-1.99 - -1.38) |
|  | Female | 1635 (921,2818) | 7.55 (3.44,13.87) | 0.14 (0.07 - 0.20) | 14253 (11617,17294) | 65.53 (51.63,81.67) | -0.25 (-0.26 - -0.24) | 25 (20,30) | 0.12 (0.09,0.15) | -2.29 (-2.79 - -1.80) | 4848 (3792,6070) | 22.41 (16.72,29) | -1.33 (-1.59 - -1.07) |
|  | Male | 1113 (586,1913) | 4.82 (1.96,9.09) | -0.12 (-0.25 - -0.00) | 9516 (7658,11748) | 41.26 (32.46,51.58) | -0.40 (-0.42 - -0.37) | 25 (19,32) | 0.11 (0.08,0.15) | -2.79 (-3.33 - -2.23) | 8527 (6824,10430) | 16.01 (12.05,20.35) | -2.03 (-2.35 - -1.71) |
| Poland | Both | 363 (169,653) | 4.71 (1.8,9.25) | -0.67 (-0.87 - -0.48) | 3198 (2543,3927) | 41.34 (32.15,51.63) | -0.46 (-0.49 - -0.44) | 1 (1,1) | 0.01 (0.01,0.01) | -6.15 (-7.37 - -4.91) | 221 (156,297) | 8.43 (5.7,11.62) | -1.62 (-1.92 - -1.33) |
|  | Female | 231 (113,417) | 6.15 (2.39,11.87) | -0.48 (-0.73 - -0.24) | 2077 (1655,2550) | 55.1 (43.05,68.64) | -0.27 (-0.30 - -0.24) | 0 (0,0) | 0.01 (0.01,0.01) | -6.39 (-8.31 - -4.44) | 430 (287,590) | 11.41 (7.43,16.11) | -1.12 (-1.27 - -0.98) |
|  | Male | 132 (57,241) | 3.34 (1.09,6.87) | -0.90 (-1.03 - -0.76) | 1122 (879,1390) | 28.26 (21.58,35.67) | -0.79 (-0.81 - -0.78) | 0 (0,0) | 0.01 (0.01,0.01) | -6.55 (-8.38 - -4.68) | 651 (443,880) | 5.59 (3.91,7.58) | -2.35 (-2.82 - -1.87) |
| Portugal | Both | 64 (27,118) | 3.35 (1.02,6.9) | -0.85 (-0.91 - -0.80) | 739 (618,853) | 36.03 (29.77,42.3) | -0.67 (-0.70 - -0.64) | 0 (0,1) | 0.02 (0.02,0.03) | -8.91 (-10.56 - -7.24) | 57 (44,71) | 6.52 (4.94,8.34) | -5.53 (-5.90 - -5.16) |
|  | Female | 33 (14,61) | 3.58 (1.05,7.32) | -0.66 (-0.70 - -0.62) | 395 (330,459) | 39.57 (32.51,47.13) | -0.55 (-0.58 - -0.52) | 0 (0,0) | 0.03 (0.02,0.03) | -8.07 (-10.31 - -5.78) | 75 (57,95) | 7.62 (5.66,9.91) | -5.07 (-5.74 - -4.38) |
|  | Male | 31 (13,58) | 3.14 (0.88,6.61) | -1.05 (-1.12 - -0.99) | 343 (284,398) | 32.64 (26.67,38.5) | -0.80 (-0.83 - -0.77) | 0 (0,0) | 0.02 (0.01,0.03) | -9.48 (-11.51 - -7.42) | 132 (102,166) | 5.47 (4.15,7) | -6.19 (-7.20 - -5.17) |
| Puerto Rico | Both | 34 (15,60) | 5.52 (2.17,10.65) | -0.20 (-0.30 - -0.10) | 455 (394,520) | 61.91 (53.28,71.01) | -0.14 (-0.17 - -0.12) | 0 (0,0) | 0.01 (0,0.01) | -5.48 (-6.74 - -4.21) | 16 (12,21) | 5.75 (4.03,7.86) | -1.26 (-1.57 - -0.95) |
|  | Female | 21 (10,37) | 6.87 (2.81,13.07) | -0.09 (-0.12 - -0.05) | 272 (236,310) | 75.55 (64.86,86.87) | -0.02 (-0.04 - -0.00) | 0 (0,0) | 0 (0,0.01) | -7.01 (-9.58 - -4.37) | 26 (18,36) | 7.3 (4.75,10.59) | -1.18 (-1.57 - -0.80) |
|  | Male | 13 (5,24) | 4.23 (1.4,8.49) | -0.45 (-0.54 - -0.36) | 183 (156,209) | 48.84 (41.56,56.47) | -0.30 (-0.31 - -0.28) | 0 (0,0) | 0.01 (0.01,0.01) | -4.54 (-6.23 - -2.81) | 42 (30,56) | 4.26 (3.09,5.65) | -1.43 (-2.05 - -0.80) |
| Qatar | Both | 60 (34,97) | 10.05 (4.73,17.84) | -0.56 (-0.61 - -0.50) | 522 (459,584) | 97.3 (84.5,110.09) | -0.67 (-0.72 - -0.61) | 0 (0,0) | 0.06 (0.03,0.08) | -3.72 (-4.84 - -2.59) | 47 (35,60) | 17.73 (11.75,25.48) | -1.85 (-2.08 - -1.62) |
|  | Female | 31 (18,52) | 10.9 (5.15,19.69) | -0.40 (-0.48 - -0.32) | 266 (234,300) | 101.89 (88.37,115.92) | -0.39 (-0.43 - -0.35) | 0 (0,0) | 0.04 (0.02,0.06) | -4.75 (-7.09 - -2.36) | 48 (34,68) | 18.21 (10.3,30.31) | -1.58 (-2.50 - -0.66) |
|  | Male | 28 (16,46) | 9.23 (4.23,16.39) | -0.78 (-0.87 - -0.69) | 256 (225,289) | 92.9 (80.71,105.59) | -0.94 (-0.99 - -0.89) | 0 (0,0) | 0.07 (0.04,0.12) | -3.32 (-4.58 - -2.05) | 95 (70,126) | 17.27 (11.4,24.83) | -1.97 (-2.49 - -1.46) |
| Republic of Korea | Both | 426 (201,777) | 5.14 (1.96,10.02) | -0.66 (-0.80 - -0.52) | 4746 (4120,5400) | 52.58 (45.02,60.63) | -0.64 (-0.66 - -0.62) | 1 (1,2) | 0.02 (0.01,0.02) | -7.10 (-7.58 - -6.62) | 365 (256,490) | 10.05 (6.79,13.84) | -2.70 (-2.86 - -2.54) |
|  | Female | 240 (112,431) | 5.95 (2.31,11.72) | -0.41 (-0.59 - -0.23) | 2712 (2346,3086) | 61.86 (52.93,71.61) | -0.50 (-0.52 - -0.49) | 1 (0,1) | 0.02 (0.01,0.03) | -6.66 (-7.46 - -5.86) | 539 (372,740) | 12.31 (7.66,18.24) | -2.06 (-2.26 - -1.86) |
|  | Male | 186 (77,345) | 4.37 (1.51,8.88) | -0.96 (-1.12 - -0.81) | 2034 (1747,2345) | 43.8 (37.07,51.09) | -0.82 (-0.85 - -0.80) | 1 (0,1) | 0.02 (0.01,0.02) | -7.66 (-8.32 - -6.99) | 903 (646,1189) | 7.92 (5.36,11.22) | -3.43 (-3.69 - -3.17) |
| Republic of Moldova | Both | 39 (19,70) | 5.5 (2.17,10.6) | 0.01 (-0.04 - 0.06) | 331 (286,378) | 46.04 (39.12,53.43) | -0.03 (-0.06 - -0.01) | 0 (0,0) | 0.01 (0.01,0.02) | -5.12 (-6.44 - -3.79) | 25 (18,34) | 9.75 (6.31,14) | -1.21 (-1.42 - -0.99) |
|  | Female | 24 (12,44) | 6.95 (2.89,13.41) | 0.10 (0.02 - 0.19) | 207 (179,237) | 59.3 (50.5,68.64) | 0.02 (-0.01 - 0.06) | 0 (0,0) | 0.02 (0.01,0.02) | -5.10 (-5.87 - -4.33) | 45 (30,66) | 12.99 (7.45,20.14) | -1.10 (-1.40 - -0.81) |
|  | Male | 15 (7,27) | 4.12 (1.38,8.38) | -0.12 (-0.14 - -0.10) | 124 (105,144) | 33.53 (27.94,39.51) | -0.10 (-0.13 - -0.08) | 0 (0,0) | 0.01 (0.01,0.02) | -4.91 (-6.64 - -3.14) | 70 (49,95) | 6.69 (4.51,9.45) | -1.51 (-1.90 - -1.11) |
| Romania | Both | 209 (100,364) | 5.14 (1.88,9.92) | -0.56 (-0.65 - -0.47) | 1950 (1674,2236) | 46.68 (39.56,54.04) | -0.51 (-0.53 - -0.49) | 1 (1,2) | 0.03 (0.02,0.04) | -4.26 (-5.30 - -3.21) | 181 (138,232) | 11.21 (7.72,15.33) | -1.84 (-2.40 - -1.27) |
|  | Female | 129 (63,229) | 6.52 (2.51,12.55) | -0.46 (-0.48 - -0.45) | 1216 (1048,1402) | 59.91 (50.89,69.37) | -0.45 (-0.46 - -0.44) | 1 (0,1) | 0.03 (0.02,0.04) | -4.22 (-5.82 - -2.60) | 285 (187,392) | 14.11 (8.31,21.19) | -1.68 (-2.24 - -1.11) |
|  | Male | 80 (34,146) | 3.83 (1.19,7.91) | -0.68 (-0.75 - -0.60) | 735 (624,846) | 34.18 (28.57,39.85) | -0.59 (-0.61 - -0.57) | 1 (1,1) | 0.03 (0.02,0.05) | -4.18 (-4.53 - -3.82) | 466 (336,607) | 8.47 (6.24,11.28) | -2.35 (-2.45 - -2.25) |
| Russian Federation | Both | 1944 (1016,3343) | 5.76 (2.45,10.73) | -0.56 (-0.63 - -0.48) | 16735 (13406,20665) | 49.45 (38.66,62.13) | -0.49 (-0.51 - -0.46) | 9 (9,10) | 0.03 (0.03,0.03) | -4.19 (-5.43 - -2.93) | 1286 (977,1644) | 11.49 (8.34,15.37) | -1.58 (-2.02 - -1.14) |
|  | Female | 1285 (689,2258) | 7.81 (3.44,14.48) | -0.36 (-0.50 - -0.21) | 11311 (9090,13885) | 68.53 (53.54,85.93) | -0.32 (-0.34 - -0.29) | 5 (4,5) | 0.03 (0.03,0.03) | -4.57 (-5.81 - -3.31) | 2562 (1834,3433) | 15.63 (11.03,21.3) | -1.30 (-1.69 - -0.91) |
|  | Male | 658 (306,1160) | 3.8 (1.32,7.56) | -0.85 (-0.96 - -0.73) | 5425 (4285,6709) | 31.3 (24.12,39.57) | -0.81 (-0.85 - -0.77) | 5 (4,5) | 0.03 (0.03,0.03) | -4.12 (-5.74 - -2.47) | 3848 (2822,5103) | 7.55 (5.65,9.75) | -2.12 (-2.89 - -1.35) |
| Rwanda | Both | 409 (221,716) | 6.36 (2.7,12.06) | -0.68 (-0.81 - -0.55) | 3678 (3202,4164) | 58.75 (50.58,67.16) | -0.58 (-0.62 - -0.54) | 4 (2,6) | 0.06 (0.03,0.1) | -5.17 (-5.56 - -4.79) | 303 (226,417) | 12.48 (8.42,17.86) | -3.28 (-3.57 - -2.99) |
|  | Female | 230 (125,399) | 7.16 (3.09,13.65) | -0.59 (-0.74 - -0.44) | 2157 (1878,2455) | 68.68 (59.43,79.01) | -0.50 (-0.53 - -0.48) | 2 (1,4) | 0.08 (0.03,0.14) | -5.08 (-5.40 - -4.76) | 487 (340,668) | 15.39 (8.56,24.63) | -3.24 (-3.40 - -3.08) |
|  | Male | 179 (92,320) | 5.58 (2.14,10.82) | -0.80 (-0.91 - -0.70) | 1521 (1320,1753) | 48.81 (41.41,56.64) | -0.70 (-0.71 - -0.68) | 1 (1,3) | 0.05 (0.02,0.09) | -5.38 (-6.42 - -4.32) | 790 (602,1022) | 9.55 (6.43,13.9) | -3.44 (-4.16 - -2.72) |
| Saint Kitts and Nevis | Both | 1 (0,1) | 5.95 (2.58,10.89) | -0.55 (-0.65 - -0.46) | 9 (8,11) | 62.83 (54.66,71.6) | -0.66 (-0.68 - -0.64) | 0 (0,0) | 0.06 (0.05,0.08) | -3.51 (-4.38 - -2.64) | 1 (0,1) | 10.3 (7.92,12.97) | -2.44 (-3.02 - -1.85) |
|  | Female | 1 (0,1) | 7.3 (3.28,13.36) | -0.68 (-0.82 - -0.54) | 6 (5,6) | 76.88 (66.83,87.41) | -0.77 (-0.80 - -0.74) | 0 (0,0) | 0.08 (0.05,0.11) | -3.81 (-4.80 - -2.81) | 1 (1,1) | 13.21 (9.78,17.22) | -2.63 (-3.25 - -2.01) |
|  | Male | 0 (0,1) | 4.62 (1.7,8.8) | -0.35 (-0.40 - -0.30) | 4 (3,4) | 48.93 (41.47,56.8) | -0.49 (-0.50 - -0.48) | 0 (0,0) | 0.05 (0.03,0.07) | -2.87 (-4.92 - -0.77) | 2 (1,2) | 7.43 (5.59,9.51) | -2.04 (-3.29 - -0.76) |
| Saint Lucia | Both | 2 (1,4) | 6.08 (2.58,11.24) | -0.70 (-0.84 - -0.57) | 29 (25,33) | 65 (56.52,73.82) | -0.53 (-0.55 - -0.52) | 0 (0,0) | 0.04 (0.03,0.06) | -3.21 (-4.04 - -2.38) | 2 (1,2) | 8.76 (6.59,11.58) | -1.95 (-2.48 - -1.42) |
|  | Female | 2 (1,3) | 7.56 (3.31,13.56) | -0.65 (-0.70 - -0.59) | 17 (15,20) | 80.15 (69.78,90.97) | -0.53 (-0.54 - -0.52) | 0 (0,0) | 0.05 (0.03,0.07) | -3.71 (-4.92 - -2.49) | 2 (2,3) | 11.22 (7.9,15.94) | -2.06 (-2.67 - -1.44) |
|  | Male | 1 (0,2) | 4.66 (1.68,9.29) | -0.69 (-0.80 - -0.57) | 12 (10,13) | 50.65 (43.37,58.36) | -0.48 (-0.50 - -0.46) | 0 (0,0) | 0.04 (0.02,0.05) | -2.05 (-3.11 - -0.98) | 4 (3,5) | 6.42 (4.9,8.27) | -1.36 (-1.78 - -0.94) |
| Saint Vincent and the Grenadines | Both | 2 (1,3) | 5.6 (2.35,10.51) | -0.98 (-1.04 - -0.94) | 21 (18,23) | 58.81 (51.16,67.07) | -0.90 (-0.92 - -0.87) | 0 (0,0) | 0.07 (0.05,0.09) | -2.94 (-3.86 - -2.02) | 1 (1,1) | 10.21 (7.85,12.89) | -2.24 (-2.73 - -1.74) |
|  | Female | 1 (1,2) | 6.98 (3.04,13.01) | -1.11 (-1.21 - -1.02) | 13 (11,14) | 72.89 (63.32,83.44) | -1.02 (-1.05 - -1.00) | 0 (0,0) | 0.1 (0.07,0.13) | -2.88 (-3.83 - -1.93) | 2 (2,3) | 14.34 (10.73,18.63) | -2.25 (-3.01 - -1.49) |
|  | Male | 1 (0,1) | 4.27 (1.51,8.42) | -0.78 (-1.06 - -0.49) | 8 (7,9) | 45.35 (38.78,52.43) | -0.66 (-0.69 - -0.63) | 0 (0,0) | 0.04 (0.03,0.05) | -2.75 (-3.42 - -2.07) | 4 (3,4) | 6.25 (4.73,8.06) | -1.86 (-2.20 - -1.52) |
| Samoa | Both | 10 (6,15) | 9.41 (4.53,16.42) | -0.15 (-0.20 - -0.10) | 96 (85,107) | 100.14 (88.41,112.56) | -0.33 (-0.35 - -0.31) | 0 (0,0) | 0.13 (0.07,0.21) | -2.11 (-2.41 - -1.81) | 13 (9,17) | 28.96 (18.56,42.41) | -1.15 (-1.32 - -0.99) |
|  | Female | 5 (3,8) | 10.86 (5.27,19.03) | -0.17 (-0.22 - -0.12) | 52 (47,59) | 113.34 (99.43,128.53) | -0.38 (-0.39 - -0.36) | 0 (0,0) | 0.14 (0.07,0.24) | -1.94 (-2.19 - -1.70) | 16 (11,22) | 33.71 (18.54,53.87) | -1.05 (-1.20 - -0.90) |
|  | Male | 4 (2,7) | 8.05 (3.68,14.35) | -0.16 (-0.20 - -0.12) | 44 (38,49) | 87.74 (76.44,99.53) | -0.31 (-0.33 - -0.28) | 0 (0,0) | 0.11 (0.05,0.21) | -2.32 (-2.88 - -1.75) | 29 (21,38) | 24.49 (13.47,39.79) | -1.31 (-1.64 - -0.98) |
| San Marino | Both | 0 (0,0) | 4.37 (1.58,8.66) | -0.23 (-0.24 - -0.21) | 3 (3,3) | 44.67 (37.71,51.85) | -0.14 (-0.15 - -0.13) | 0 (0,0) | 0.01 (0.01,0.02) | -6.66 (-6.99 - -6.34) | 0 (0,0) | 6.67 (4.7,8.99) | -2.19 (-2.32 - -2.05) |
|  | Female | 0 (0,0) | 4.71 (1.71,9.37) | -0.17 (-0.27 - -0.06) | 2 (1,2) | 49.87 (42.02,58.17) | -0.07 (-0.09 - -0.05) | 0 (0,0) | 0.01 (0.01,0.02) | -6.06 (-6.48 - -5.63) | 0 (0,0) | 8.07 (5.43,11.26) | -1.92 (-2.04 - -1.80) |
|  | Male | 0 (0,0) | 4.06 (1.36,8.34) | -0.29 (-0.30 - -0.27) | 1 (1,2) | 39.88 (33.24,46.71) | -0.19 (-0.22 - -0.17) | 0 (0,0) | 0.01 (0,0.01) | -7.45 (-7.75 - -7.15) | 0 (0,1) | 5.37 (3.74,7.25) | -2.55 (-2.68 - -2.42) |
| Sao Tome and Principe | Both | 12 (8,19) | 11.95 (6.42,19.97) | 0.00 (-0.02 - 0.02) | 108 (97,119) | 106.15 (94.24,118.03) | -0.49 (-0.50 - -0.48) | 0 (0,0) | 0.17 (0.08,0.3) | -3.72 (-5.25 - -2.17) | 14 (9,22) | 26.84 (16.9,40.07) | -2.73 (-3.71 - -1.75) |
|  | Female | 6 (4,9) | 12.11 (6.39,20.37) | -0.40 (-0.42 - -0.38) | 68 (61,75) | 133.48 (118.21,148.35) | -0.63 (-0.64 - -0.62) | 0 (0,0) | 0.09 (0.04,0.18) | -1.98 (-2.72 - -1.24) | 13 (9,17) | 25.04 (14.29,39.99) | -1.22 (-1.48 - -0.96) |
|  | Male | 6 (4,9) | 11.88 (6.27,20.1) | 0.48 (0.43 - 0.52) | 40 (35,45) | 79.14 (68.74,90.13) | -0.26 (-0.27 - -0.26) | 0 (0,0) | 0.24 (0.1,0.49) | -4.13 (-5.69 - -2.54) | 27 (19,37) | 28.58 (15.42,47.93) | -3.59 (-4.85 - -2.31) |
| Saudi Arabia | Both | 952 (566,1544) | 9.41 (4.52,16.41) | 0.23 (0.12 - 0.34) | 8974 (7973,10026) | 87.11 (76.38,98.12) | 0.16 (0.14 - 0.18) | 13 (9,19) | 0.13 (0.07,0.2) | -5.99 (-6.29 - -5.68) | 1096 (790,1466) | 21.92 (15.48,29.81) | -4.29 (-4.46 - -4.11) |
|  | Female | 490 (290,777) | 10.37 (5.14,17.98) | 0.20 (0.11 - 0.29) | 4421 (3902,4947) | 93.46 (81.47,105.6) | 0.15 (0.13 - 0.16) | 6 (4,10) | 0.14 (0.07,0.23) | -5.71 (-5.94 - -5.48) | 1158 (863,1528) | 24.6 (15.86,35.45) | -3.91 (-4.07 - -3.76) |
|  | Male | 462 (267,771) | 8.6 (3.93,15.38) | 0.29 (0.15 - 0.43) | 4553 (4032,5122) | 81.82 (71.26,93.28) | 0.20 (0.18 - 0.21) | 7 (4,11) | 0.12 (0.06,0.22) | -6.24 (-6.40 - -6.07) | 2254 (1710,2853) | 19.74 (12.88,29.66) | -4.69 (-4.81 - -4.57) |
| Senegal | Both | 917 (582,1435) | 11.28 (5.95,19.05) | -0.58 (-0.70 - -0.46) | 8457 (7569,9389) | 108.5 (96.18,121.73) | -0.63 (-0.65 - -0.61) | 22 (15,30) | 0.27 (0.16,0.42) | -3.63 (-3.98 - -3.28) | 1664 (1101,2363) | 36.01 (24.27,51.01) | -2.96 (-3.23 - -2.69) |
|  | Female | 512 (332,792) | 12.73 (6.78,21.41) | -0.46 (-0.54 - -0.39) | 5441 (4921,6026) | 144.12 (128.4,161.45) | -0.48 (-0.49 - -0.46) | 7 (3,10) | 0.17 (0.08,0.3) | -2.47 (-2.78 - -2.15) | 1261 (921,1642) | 32.88 (19.2,50.38) | -1.60 (-1.77 - -1.44) |
|  | Male | 405 (241,648) | 9.88 (4.87,17.41) | -0.75 (-0.80 - -0.69) | 3016 (2629,3414) | 75.41 (65.06,86.86) | -0.76 (-0.78 - -0.75) | 16 (9,23) | 0.36 (0.19,0.62) | -4.25 (-5.20 - -3.28) | 2925 (2196,3658) | 39.08 (23.23,61.25) | -3.80 (-4.60 - -2.99) |
| Serbia | Both | 92 (45,162) | 4.81 (1.83,9.34) | -1.01 (-1.10 - -0.92) | 1004 (816,1215) | 48.58 (38.64,59.99) | -0.90 (-0.93 - -0.86) | 0 (0,1) | 0.02 (0.01,0.03) | -7.09 (-9.05 - -5.08) | 77 (51,106) | 10.39 (6.47,15.33) | -2.96 (-3.23 - -2.69) |
|  | Female | 56 (29,101) | 6.19 (2.4,11.86) | -0.84 (-0.95 - -0.72) | 636 (511,770) | 65.33 (51.74,80.97) | -0.80 (-0.84 - -0.76) | 0 (0,0) | 0.02 (0.01,0.03) | -6.53 (-7.73 - -5.32) | 137 (87,207) | 14.1 (7.96,22.31) | -2.31 (-2.52 - -2.09) |
|  | Male | 36 (15,65) | 3.56 (1.07,7.14) | -1.10 (-1.17 - -1.03) | 368 (298,442) | 33.64 (26.74,41.45) | -0.90 (-0.92 - -0.88) | 0 (0,0) | 0.02 (0.01,0.03) | -7.59 (-9.42 - -5.73) | 214 (144,302) | 7.07 (4.55,10.24) | -3.95 (-4.83 - -3.06) |
| Seychelles | Both | 2 (1,3) | 5.93 (2.5,10.85) | -0.05 (-0.09 - -0.01) | 16 (14,18) | 53.67 (46.04,61.45) | -0.15 (-0.17 - -0.14) | 0 (0,0) | 0.02 (0.01,0.03) | -0.58 (-1.60 - 0.44) | 1 (1,2) | 11.77 (7.47,16.57) | -0.19 (-0.45 - 0.08) |
|  | Female | 1 (1,2) | 6.85 (2.86,12.85) | -0.05 (-0.07 - -0.02) | 9 (8,10) | 62.15 (53.36,71.5) | -0.10 (-0.12 - -0.09) | 0 (0,0) | 0.02 (0.01,0.04) | -1.29 (-2.16 - -0.41) | 2 (1,3) | 14.67 (8.51,22.27) | -0.54 (-1.08 - 0.01) |
|  | Male | 1 (0,1) | 5.05 (2.03,9.34) | -0.07 (-0.10 - -0.06) | 7 (6,8) | 45.49 (38.33,52.73) | -0.19 (-0.20 - -0.18) | 0 (0,0) | 0.01 (0.01,0.02) | -0.00 (-1.72 - 1.74) | 4 (3,5) | 8.97 (5.76,12.83) | 0.07 (-0.40 - 0.55) |
| Sierra Leone | Both | 536 (357,814) | 11.57 (6.53,18.61) | -0.55 (-0.61 - -0.49) | 4930 (4495,5381) | 114.14 (102.71,125.41) | -0.53 (-0.54 - -0.52) | 43 (19,75) | 0.87 (0.36,1.6) | -2.57 (-3.20 - -1.94) | 2891 (1076,5174) | 88.84 (42.92,152.61) | -2.37 (-2.95 - -1.79) |
|  | Female | 330 (226,493) | 13.85 (8.01,22.13) | -0.32 (-0.40 - -0.24) | 3424 (3123,3721) | 155.93 (140.57,171.24) | -0.34 (-0.36 - -0.32) | 12 (6,23) | 0.49 (0.23,1.04) | -1.82 (-2.40 - -1.23) | 1453 (947,2434) | 61.93 (34.25,112.22) | -1.49 (-1.94 - -1.04) |
|  | Male | 206 (128,329) | 9.38 (4.82,16.2) | -0.86 (-0.91 - -0.82) | 1506 (1338,1685) | 71.96 (62.9,81.47) | -0.93 (-0.98 - -0.87) | 31 (10,57) | 1.24 (0.4,2.42) | -2.83 (-3.52 - -2.12) | 4344 (2208,7185) | 114.97 (41.41,217.29) | -2.75 (-3.44 - -2.07) |
| Singapore | Both | 41 (18,74) | 3.88 (1.35,7.88) | -0.88 (-0.98 - -0.77) | 399 (344,455) | 39.94 (33.89,46.3) | -0.74 (-0.77 - -0.71) | 0 (0,0) | 0.01 (0.01,0.01) | -7.02 (-9.79 - -4.17) | 28 (20,38) | 7.31 (5.18,9.88) | -2.24 (-2.54 - -1.93) |
|  | Female | 22 (10,40) | 4.34 (1.49,8.68) | -0.72 (-0.81 - -0.63) | 223 (192,255) | 45.89 (38.9,53.21) | -0.56 (-0.61 - -0.52) | 0 (0,0) | 0.01 (0.01,0.02) | -6.04 (-8.63 - -3.38) | 45 (32,60) | 9.19 (6.26,12.87) | -2.15 (-2.61 - -1.70) |
|  | Male | 18 (8,35) | 3.43 (1.04,7.26) | -1.06 (-1.18 - -0.94) | 175 (149,204) | 34.28 (28.6,40.62) | -0.97 (-1.01 - -0.94) | 0 (0,0) | 0 (0,0) | -8.22 (-8.63 - -7.81) | 73 (53,97) | 5.55 (3.89,7.38) | -2.33 (-2.49 - -2.17) |
| Slovakia | Both | 63 (33,111) | 5.65 (2.31,10.64) | -0.42 (-0.47 - -0.38) | 568 (491,648) | 50.89 (43.56,58.56) | -0.28 (-0.31 - -0.26) | 0 (0,0) | 0.02 (0.01,0.03) | -3.47 (-4.63 - -2.31) | 47 (33,61) | 11.18 (7.3,15.98) | -1.12 (-1.24 - -1.00) |
|  | Female | 39 (21,69) | 7.11 (2.95,13.55) | -0.28 (-0.34 - -0.22) | 350 (303,398) | 64.32 (55.07,74.19) | -0.17 (-0.18 - -0.16) | 0 (0,0) | 0.02 (0.01,0.04) | -3.42 (-4.84 - -1.98) | 78 (50,108) | 14.34 (8.37,22.13) | -0.88 (-1.10 - -0.67) |
|  | Male | 24 (12,43) | 4.27 (1.52,8.32) | -0.61 (-0.66 - -0.55) | 219 (187,252) | 38.14 (32.12,44.52) | -0.43 (-0.45 - -0.42) | 0 (0,0) | 0.02 (0.01,0.04) | -4.06 (-4.47 - -3.64) | 125 (85,167) | 8.18 (5.61,11.53) | -1.69 (-1.76 - -1.61) |
| Slovenia | Both | 15 (7,27) | 3.65 (1.3,7.36) | -1.25 (-1.34 - -1.15) | 136 (116,156) | 33.74 (28.3,39.22) | -1.12 (-1.16 - -1.08) | 0 (0,0) | 0 (0,0) | -8.16 (-10.85 - -5.40) | 10 (7,13) | 6.52 (4.48,9.05) | -1.66 (-1.76 - -1.55) |
|  | Female | 9 (4,16) | 4.54 (1.59,9) | -1.33 (-1.48 - -1.18) | 83 (71,95) | 42.44 (35.64,49.28) | -1.25 (-1.32 - -1.17) | 0 (0,0) | 0 (0,0) | -7.82 (-8.71 - -6.93) | 17 (12,24) | 8.56 (5.68,12.22) | -1.56 (-1.65 - -1.48) |
|  | Male | 6 (2,11) | 2.82 (0.78,6.13) | -1.10 (-1.22 - -0.99) | 53 (44,62) | 25.54 (21.05,30.39) | -0.90 (-0.91 - -0.88) | 0 (0,0) | 0 (0,0) | -8.31 (-12.13 - -4.32) | 26 (18,36) | 4.59 (3.17,6.15) | -1.83 (-2.08 - -1.59) |
| Solomon Islands | Both | 31 (20,48) | 9.24 (4.72,15.69) | -0.22 (-0.26 - -0.18) | 326 (293,359) | 102.76 (91.38,114.01) | -0.38 (-0.39 - -0.37) | 0 (0,0) | 0.07 (0.04,0.12) | -2.15 (-2.83 - -1.46) | 31 (21,43) | 24.99 (15.33,36.72) | -0.99 (-1.18 - -0.80) |
|  | Female | 18 (11,27) | 11.13 (5.87,18.97) | -0.17 (-0.20 - -0.14) | 185 (165,204) | 121.71 (108.18,135.63) | -0.37 (-0.38 - -0.36) | 0 (0,0) | 0.1 (0.04,0.19) | -2.28 (-2.91 - -1.65) | 50 (35,67) | 32.47 (17.85,50.82) | -1.04 (-1.25 - -0.82) |
|  | Male | 13 (8,21) | 7.5 (3.64,13.07) | -0.30 (-0.34 - -0.25) | 141 (126,156) | 85.29 (74.99,95.76) | -0.39 (-0.41 - -0.37) | 0 (0,0) | 0.04 (0.02,0.09) | -2.21 (-3.37 - -1.04) | 81 (58,106) | 18.11 (9.48,30.31) | -0.85 (-1.14 - -0.57) |
| Somalia | Both | 960 (538,1613) | 7.57 (3.41,13.86) | -0.13 (-0.29 - 0.03) | 7460 (6546,8443) | 65.14 (56.38,74.36) | -0.21 (-0.24 - -0.18) | 10 (6,18) | 0.08 (0.04,0.15) | -2.08 (-2.29 - -1.87) | 760 (475,1228) | 14.79 (9.35,22.59) | -1.22 (-1.35 - -1.09) |
|  | Female | 527 (298,898) | 8.53 (3.92,15.47) | -0.05 (-0.20 - 0.10) | 4201 (3703,4757) | 76.2 (65.99,86.93) | -0.18 (-0.21 - -0.15) | 6 (3,10) | 0.1 (0.03,0.19) | -2.09 (-2.29 - -1.90) | 1057 (698,1515) | 18.03 (9.64,28.76) | -1.17 (-1.32 - -1.02) |
|  | Male | 433 (228,731) | 6.68 (2.85,12.61) | -0.21 (-0.34 - -0.08) | 3260 (2819,3759) | 54.98 (47.03,63.49) | -0.30 (-0.32 - -0.28) | 5 (2,10) | 0.07 (0.02,0.16) | -2.12 (-2.41 - -1.82) | 1817 (1305,2574) | 11.8 (6.62,20.88) | -1.30 (-1.47 - -1.14) |
| South Africa | Both | 1475 (806,2601) | 7.41 (3.21,13.51) | -0.77 (-0.87 - -0.66) | 13992 (11292,17125) | 70.39 (55.69,87.51) | -0.82 (-0.86 - -0.78) | 13 (11,16) | 0.07 (0.05,0.09) | -3.18 (-4.16 - -2.20) | 1094 (890,1335) | 14.59 (10.96,18.87) | -1.95 (-2.33 - -1.57) |
|  | Female | 947 (520,1635) | 9.63 (4.32,17.4) | -0.80 (-0.93 - -0.67) | 9227 (7458,11229) | 93.91 (74.53,116.14) | -0.86 (-0.91 - -0.82) | 7 (5,8) | 0.07 (0.05,0.09) | -3.58 (-5.15 - -2.00) | 1801 (1372,2243) | 18.34 (13.2,24.37) | -1.96 (-2.47 - -1.45) |
|  | Male | 528 (247,917) | 5.24 (2.01,10.12) | -0.68 (-0.88 - -0.47) | 4765 (3835,5899) | 47.41 (36.88,59.51) | -0.63 (-0.66 - -0.61) | 7 (5,8) | 0.07 (0.05,0.09) | -2.60 (-3.05 - -2.16) | 2895 (2280,3584) | 10.93 (8.31,14.04) | -1.84 (-2.08 - -1.61) |
| South Sudan | Both | 369 (210,641) | 6.75 (2.95,12.3) | -0.11 (-0.22 - -0.01) | 3255 (2863,3676) | 61.99 (53.73,70.99) | -0.21 (-0.22 - -0.20) | 12 (8,21) | 0.21 (0.12,0.39) | -0.98 (-1.42 - -0.55) | 668 (440,1022) | 25.97 (16.52,41.89) | -0.78 (-1.03 - -0.54) |
|  | Female | 199 (113,341) | 7.52 (3.3,14.12) | -0.06 (-0.17 - 0.05) | 1819 (1598,2070) | 72.19 (62.75,82.81) | -0.18 (-0.20 - -0.17) | 6 (3,13) | 0.24 (0.11,0.47) | -0.66 (-1.12 - -0.20) | 795 (511,1314) | 29.55 (16.15,51.45) | -0.55 (-0.82 - -0.27) |
|  | Male | 171 (94,291) | 6.03 (2.53,11.13) | -0.15 (-0.26 - -0.04) | 1436 (1248,1632) | 52.61 (44.69,60.65) | -0.24 (-0.25 - -0.24) | 6 (3,10) | 0.19 (0.1,0.37) | -1.28 (-1.68 - -0.89) | 1464 (1016,2109) | 22.67 (13.2,37.91) | -1.06 (-1.38 - -0.74) |
| Spain | Both | 310 (148,518) | 3.54 (1.2,6.83) | -1.40 (-1.57 - -1.22) | 3323 (2883,3799) | 35.72 (30.48,41.47) | -0.79 (-0.82 - -0.76) | 1 (1,1) | 0.01 (0.01,0.01) | -7.39 (-8.25 - -6.52) | 216 (160,285) | 5.59 (4.16,7.32) | -3.23 (-3.54 - -2.92) |
|  | Female | 159 (72,268) | 3.75 (1.25,7.21) | -1.16 (-1.31 - -1.01) | 1782 (1540,2023) | 39.4 (33.55,45.78) | -0.72 (-0.74 - -0.70) | 1 (0,1) | 0.01 (0.01,0.02) | -6.57 (-9.80 - -3.23) | 298 (225,383) | 6.67 (4.96,8.66) | -2.80 (-3.09 - -2.50) |
|  | Male | 150 (70,255) | 3.33 (1.07,6.61) | -1.66 (-1.89 - -1.44) | 1541 (1326,1786) | 32.24 (27.24,37.71) | -0.86 (-0.92 - -0.79) | 0 (0,0) | 0.01 (0.01,0.01) | -8.07 (-10.94 - -5.11) | 514 (384,670) | 4.56 (3.34,6.02) | -3.75 (-4.11 - -3.38) |
| Sri Lanka | Both | 404 (223,685) | 5.82 (2.53,10.84) | -0.50 (-0.55 - -0.45) | 3873 (3388,4398) | 54.02 (46.5,62.04) | -0.56 (-0.61 - -0.52) | 6 (4,8) | 0.08 (0.05,0.12) | -2.75 (-3.38 - -2.12) | 476 (355,616) | 16.47 (11.87,22.3) | -1.65 (-1.94 - -1.35) |
|  | Female | 243 (133,416) | 7.06 (3.11,13.24) | -0.56 (-0.68 - -0.43) | 2311 (2017,2639) | 64.99 (56.01,74.65) | -0.58 (-0.64 - -0.53) | 3 (2,4) | 0.09 (0.05,0.14) | -2.64 (-4.95 - -0.27) | 703 (506,954) | 19.79 (12.97,28.38) | -1.45 (-1.88 - -1.03) |
|  | Male | 161 (86,275) | 4.61 (1.77,8.61) | -0.41 (-0.48 - -0.35) | 1561 (1334,1801) | 43.23 (36.34,50.31) | -0.52 (-0.56 - -0.48) | 3 (2,4) | 0.08 (0.04,0.12) | -2.77 (-3.63 - -1.91) | 1179 (901,1519) | 13.21 (9.12,17.88) | -1.62 (-2.03 - -1.21) |
| Sudan | Both | 2279 (1438,3635) | 10.72 (5.72,18.08) | -0.10 (-0.26 - 0.06) | 20093 (18041,22109) | 96.34 (85.6,107.58) | -0.28 (-0.29 - -0.26) | 127 (78,195) | 0.6 (0.3,1.03) | -3.46 (-3.63 - -3.29) | 6370 (3474,10195) | 62.44 (36.64,99.61) | -3.12 (-3.24 - -3.00) |
|  | Female | 1185 (745,1905) | 11.55 (6.11,19.58) | -0.10 (-0.20 - 0.01) | 10211 (9117,11290) | 101.38 (89.31,114.23) | -0.27 (-0.29 - -0.25) | 66 (33,134) | 0.65 (0.28,1.39) | -4.02 (-4.18 - -3.87) | 6943 (4137,12460) | 67.81 (35.84,129.06) | -3.64 (-3.73 - -3.56) |
|  | Male | 1094 (687,1705) | 9.94 (5.21,16.7) | -0.11 (-0.21 - -0.00) | 9883 (8785,10928) | 91.62 (80.68,102.62) | -0.27 (-0.28 - -0.25) | 61 (28,107) | 0.55 (0.22,1.08) | -2.69 (-2.92 - -2.45) | 13313 (8931,19040) | 57.43 (28.31,103.44) | -2.44 (-2.63 - -2.23) |
| Suriname | Both | 11 (6,18) | 5.99 (2.69,10.72) | -0.55 (-0.66 - -0.44) | 122 (107,136) | 62.95 (55.26,71.2) | -0.60 (-0.63 - -0.57) | 0 (0,0) | 0.09 (0.06,0.14) | -2.45 (-3.46 - -1.42) | 9 (7,12) | 12.82 (9.19,17.44) | -1.91 (-2.51 - -1.31) |
|  | Female | 7 (4,12) | 7.49 (3.41,13.47) | -0.49 (-0.61 - -0.38) | 73 (65,82) | 78 (68.45,88.58) | -0.59 (-0.61 - -0.57) | 0 (0,0) | 0.11 (0.06,0.19) | -2.48 (-3.52 - -1.44) | 15 (12,19) | 16.32 (10.94,23.33) | -1.80 (-2.32 - -1.27) |
|  | Male | 4 (2,7) | 4.57 (1.77,8.57) | -0.58 (-0.74 - -0.42) | 49 (43,56) | 48.85 (42.13,56.36) | -0.60 (-0.63 - -0.57) | 0 (0,0) | 0.07 (0.04,0.13) | -2.51 (-4.66 - -0.31) | 24 (19,30) | 9.53 (6.27,14.23) | -2.05 (-3.44 - -0.64) |
| Sweden | Both | 163 (84,291) | 6.74 (2.67,12.95) | 1.12 (0.99 - 1.24) | 1721 (1359,2119) | 70.23 (54.55,87.59) | 0.98 (0.95 - 1.00) | 0 (0,0) | 0 (0,0.01) | -7.72 (-9.29 - -6.13) | 106 (72,148) | 9.47 (6.2,13.42) | -0.54 (-0.66 - -0.43) |
|  | Female | 78 (38,146) | 6.68 (2.61,13.1) | 1.21 (1.03 - 1.40) | 864 (676,1069) | 72.92 (56.34,91.4) | 1.07 (1.04 - 1.10) | 0 (0,0) | 0.01 (0,0.01) | -7.17 (-9.20 - -5.11) | 126 (83,177) | 10.62 (6.63,15.57) | -0.24 (-0.75 - 0.27) |
|  | Male | 85 (44,154) | 6.79 (2.68,12.99) | 0.98 (0.83 - 1.14) | 857 (681,1060) | 67.7 (52.02,84.58) | 0.89 (0.87 - 0.91) | 0 (0,0) | 0 (0,0.01) | -8.20 (-8.97 - -7.42) | 232 (156,319) | 8.39 (5.45,12.05) | -0.90 (-1.04 - -0.75) |
| Switzerland | Both | 59 (25,105) | 3.35 (1.05,6.81) | -0.84 (-0.95 - -0.72) | 612 (520,705) | 34.64 (28.92,40.5) | -0.53 (-0.55 - -0.52) | 0 (0,0) | 0.01 (0,0.01) | -9.39 (-9.72 - -9.06) | 37 (27,49) | 4.97 (3.53,6.59) | -3.21 (-3.35 - -3.08) |
|  | Female | 31 (14,55) | 3.58 (1.1,7.24) | -0.80 (-0.95 - -0.64) | 326 (278,378) | 38.11 (31.78,44.92) | -0.47 (-0.48 - -0.45) | 0 (0,0) | 0.01 (0,0.01) | -8.79 (-9.23 - -8.35) | 51 (37,67) | 5.91 (4.21,7.89) | -2.76 (-3.11 - -2.40) |
|  | Male | 28 (11,52) | 3.12 (0.83,6.52) | -0.88 (-0.91 - -0.86) | 286 (240,331) | 31.38 (25.9,37.14) | -0.60 (-0.62 - -0.58) | 0 (0,0) | 0 (0,0.01) | -9.81 (-10.30 - -9.31) | 88 (63,115) | 4.08 (2.87,5.45) | -3.71 (-3.88 - -3.53) |
| Syrian Arab Republic | Both | 658 (422,984) | 11.54 (6.44,18.77) | -1.02 (-1.10 - -0.93) | 6073 (5442,6748) | 97.32 (86.19,108.93) | -1.21 (-1.24 - -1.18) | 19 (14,26) | 0.32 (0.2,0.46) | -5.05 (-5.83 - -4.28) | 1171 (863,1569) | 38.1 (27,51.5) | -4.38 (-4.94 - -3.81) |
|  | Female | 332 (212,510) | 11.7 (6.23,19.46) | -0.88 (-0.95 - -0.80) | 3051 (2695,3421) | 98.19 (85.79,111.44) | -1.05 (-1.07 - -1.03) | 9 (6,12) | 0.29 (0.17,0.45) | -5.45 (-6.19 - -4.70) | 1120 (812,1441) | 37.4 (24.41,53.6) | -4.58 (-5.13 - -4.03) |
|  | Male | 326 (215,477) | 11.38 (6.34,18.44) | -1.14 (-1.30 - -0.97) | 3022 (2715,3353) | 96.46 (85.73,108.2) | -1.35 (-1.40 - -1.30) | 10 (7,15) | 0.34 (0.19,0.56) | -4.74 (-5.60 - -3.88) | 2291 (1784,2864) | 38.79 (26.07,57.79) | -4.21 (-4.91 - -3.50) |
| Taiwan (Province of China) | Both | 293 (156,505) | 7.29 (3.06,13.38) | -0.03 (-0.10 - 0.05) | 3051 (2673,3444) | 72.62 (63.12,82.88) | -0.06 (-0.08 - -0.04) | 1 (1,1) | 0.02 (0.02,0.02) | -2.90 (-4.61 - -1.15) | 266 (192,357) | 15.37 (9.87,22.18) | -0.53 (-0.65 - -0.40) |
|  | Female | 167 (90,283) | 8.64 (3.69,15.54) | 0.10 (0.01 - 0.19) | 1755 (1540,1996) | 87.18 (75.51,99.53) | 0.02 (-0.01 - 0.05) | 0 (0,0) | 0.02 (0.01,0.02) | -3.44 (-4.64 - -2.23) | 380 (234,546) | 18.88 (10.15,30.29) | -0.39 (-0.52 - -0.26) |
|  | Male | 126 (64,229) | 6.04 (2.41,11.53) | -0.18 (-0.25 - -0.10) | 1296 (1128,1486) | 59.21 (50.85,68.45) | -0.15 (-0.17 - -0.14) | 1 (0,1) | 0.02 (0.02,0.03) | -2.78 (-4.70 - -0.82) | 646 (441,878) | 12.13 (8.35,17.17) | -0.64 (-0.98 - -0.30) |
| Tajikistan | Both | 239 (124,418) | 5.55 (2.27,10.42) | -0.38 (-0.54 - -0.23) | 1800 (1559,2061) | 44.54 (37.62,51.81) | -0.31 (-0.36 - -0.25) | 4 (3,6) | 0.1 (0.06,0.15) | -2.71 (-3.44 - -1.97) | 280 (201,381) | 16.12 (10.9,22.58) | -1.75 (-2.18 - -1.32) |
|  | Female | 148 (78,268) | 7.1 (3.04,13.32) | -0.24 (-0.37 - -0.11) | 1122 (971,1281) | 57.33 (48.51,66.56) | -0.16 (-0.22 - -0.11) | 2 (1,3) | 0.11 (0.06,0.17) | -2.52 (-3.05 - -1.99) | 393 (281,512) | 19.64 (12.28,28.97) | -1.38 (-1.72 - -1.03) |
|  | Male | 91 (41,162) | 4.1 (1.27,8.26) | -0.55 (-0.72 - -0.38) | 678 (578,788) | 32.52 (27.08,38.37) | -0.48 (-0.49 - -0.47) | 2 (1,3) | 0.09 (0.05,0.15) | -3.03 (-3.82 - -2.24) | 674 (500,862) | 12.81 (8.46,18.61) | -2.21 (-2.81 - -1.62) |
| Thailand | Both | 719 (371,1220) | 5.21 (2.11,9.86) | -0.27 (-0.35 - -0.19) | 7063 (6126,7992) | 48.07 (41.06,55.23) | -0.32 (-0.34 - -0.29) | 7 (5,10) | 0.05 (0.03,0.08) | -2.18 (-2.85 - -1.50) | 865 (630,1137) | 12.88 (8.92,17.45) | -1.04 (-1.33 - -0.75) |
|  | Female | 418 (218,732) | 6.18 (2.57,11.72) | -0.25 (-0.32 - -0.17) | 4096 (3536,4678) | 56.77 (48.33,65.57) | -0.27 (-0.30 - -0.24) | 3 (2,4) | 0.04 (0.02,0.06) | -3.10 (-4.24 - -1.94) | 1028 (704,1408) | 14.33 (8.97,21.57) | -1.14 (-1.44 - -0.84) |
|  | Male | 301 (141,520) | 4.28 (1.51,8.32) | -0.34 (-0.48 - -0.19) | 2967 (2546,3370) | 39.68 (33.65,45.85) | -0.36 (-0.38 - -0.34) | 5 (3,7) | 0.06 (0.03,0.1) | -1.43 (-2.23 - -0.62) | 1892 (1417,2414) | 11.5 (7.98,15.9) | -0.90 (-1.27 - -0.52) |
| Timor-Leste | Both | 37 (20,64) | 5.41 (2.27,9.95) | -0.04 (-0.18 - 0.10) | 320 (279,361) | 47.33 (40.75,54.16) | -0.26 (-0.28 - -0.23) | 1 (1,2) | 0.17 (0.08,0.29) | -2.86 (-3.31 - -2.40) | 75 (41,114) | 22.84 (13.7,33.6) | -2.20 (-2.48 - -1.93) |
|  | Female | 21 (11,37) | 6.31 (2.59,11.92) | -0.00 (-0.17 - 0.16) | 183 (159,209) | 55.73 (47.74,64.33) | -0.20 (-0.21 - -0.19) | 1 (0,1) | 0.16 (0.08,0.27) | -3.36 (-3.85 - -2.87) | 82 (61,106) | 24.54 (15.34,36.3) | -2.42 (-2.71 - -2.12) |
|  | Male | 16 (9,27) | 4.55 (1.77,8.4) | -0.10 (-0.23 - 0.03) | 137 (118,156) | 39.39 (33.67,45.32) | -0.35 (-0.37 - -0.32) | 1 (0,1) | 0.18 (0.06,0.35) | -2.33 (-2.81 - -1.84) | 157 (105,208) | 21.22 (10.3,34.98) | -1.95 (-2.27 - -1.63) |
| Togo | Both | 424 (264,684) | 10.23 (5.44,17.37) | -0.60 (-0.64 - -0.56) | 3769 (3399,4147) | 95.09 (84.84,105.64) | -0.75 (-0.79 - -0.71) | 14 (7,22) | 0.32 (0.15,0.54) | -2.42 (-2.64 - -2.20) | 1033 (574,1568) | 38.5 (22.4,58.64) | -2.05 (-2.37 - -1.73) |
|  | Female | 230 (144,370) | 11.05 (5.74,19.09) | -0.60 (-0.70 - -0.50) | 2369 (2135,2606) | 121.75 (108.63,135.48) | -0.68 (-0.71 - -0.65) | 3 (2,5) | 0.17 (0.07,0.31) | -1.74 (-2.01 - -1.48) | 584 (413,797) | 29.61 (16.44,46.36) | -1.24 (-1.38 - -1.10) |
|  | Male | 194 (119,317) | 9.4 (4.75,16.21) | -0.62 (-0.69 - -0.55) | 1400 (1231,1562) | 69.84 (60.75,78.89) | -0.81 (-0.84 - -0.77) | 10 (5,17) | 0.47 (0.2,0.84) | -2.65 (-2.89 - -2.41) | 1617 (1030,2249) | 47.14 (23.77,78.98) | -2.44 (-2.71 - -2.18) |
| Tokelau | Both | 0 (0,0) | 8.07 (3.8,14.25) | 0.26 (0.23 - 0.29) | 0 (0,1) | 90.21 (79.49,101.67) | 0.01 (-0.04 - 0.07) | 0 (0,0) | 0.63 (0.25,1.11) | 4.13 (1.99 - 6.32) | 0 (0,0) | 69.09 (34.55,111.93) | 2.58 (1.44 - 3.74) |
|  | Female | 0 (0,0) | 9.67 (4.59,16.97) | 0.18 (0.04 - 0.32) | 0 (0,0) | 109.02 (95.93,123.24) | -0.08 (-0.12 - -0.04) | 0 (0,0) | 0.73 (0.3,1.27) | 3.31 (1.65 - 5.01) | 0 (0,0) | 81.11 (42.59,129.91) | 1.95 (0.99 - 2.92) |
|  | Male | 0 (0,0) | 6.65 (2.79,12.45) | 0.41 (0.27 - 0.54) | 0 (0,0) | 73.48 (63.92,83.98) | 0.17 (0.15 - 0.18) | 0 (0,0) | 0.54 (0.15,1.16) | 5.56 (3.35 - 7.83) | 0 (0,0) | 58.62 (22.48,113.91) | 3.64 (2.52 - 4.78) |
| Tonga | Both | 4 (2,7) | 8.6 (4.05,14.82) | -0.15 (-0.18 - -0.12) | 44 (40,49) | 94.17 (82.9,105.55) | -0.25 (-0.26 - -0.23) | 0 (0,0) | 0.1 (0.06,0.15) | -1.40 (-1.92 - -0.87) | 5 (4,7) | 25.53 (16.63,36.87) | -0.66 (-0.85 - -0.48) |
|  | Female | 2 (1,4) | 9.8 (4.66,17.22) | -0.20 (-0.22 - -0.17) | 24 (22,27) | 107.84 (94.31,121.53) | -0.26 (-0.28 - -0.25) | 0 (0,0) | 0.11 (0.05,0.19) | -1.14 (-1.73 - -0.54) | 7 (5,10) | 30.28 (17.02,48.25) | -0.57 (-0.78 - -0.35) |
|  | Male | 2 (1,3) | 7.5 (3.32,13.39) | -0.09 (-0.16 - -0.03) | 20 (18,22) | 81.53 (71.13,92.06) | -0.22 (-0.23 - -0.20) | 0 (0,0) | 0.09 (0.04,0.14) | -1.66 (-2.21 - -1.11) | 12 (9,17) | 21.12 (12.65,32.57) | -0.81 (-1.15 - -0.48) |
| Trinidad and Tobago | Both | 26 (15,43) | 7.2 (3.28,12.88) | -0.42 (-0.51 - -0.32) | 286 (253,319) | 76.29 (66.92,86.07) | -0.43 (-0.45 - -0.41) | 0 (0,0) | 0.05 (0.04,0.07) | -3.54 (-4.40 - -2.68) | 16 (13,20) | 10.63 (8.02,14.08) | -2.06 (-2.41 - -1.72) |
|  | Female | 16 (9,26) | 8.98 (4.27,15.77) | -0.43 (-0.50 - -0.37) | 172 (153,193) | 93.49 (81.97,105.61) | -0.49 (-0.50 - -0.47) | 0 (0,0) | 0.05 (0.04,0.07) | -3.31 (-3.87 - -2.75) | 23 (18,31) | 12.77 (9.03,17.84) | -1.78 (-2.22 - -1.33) |
|  | Male | 10 (5,17) | 5.49 (2.19,10.38) | -0.35 (-0.41 - -0.29) | 114 (99,130) | 59.71 (51.5,68.68) | -0.33 (-0.34 - -0.31) | 0 (0,0) | 0.05 (0.04,0.08) | -3.33 (-4.80 - -1.84) | 39 (31,50) | 8.57 (6.46,11.03) | -2.30 (-3.23 - -1.36) |
| Tunisia | Both | 292 (168,498) | 8.2 (3.81,14.8) | -0.07 (-0.19 - 0.04) | 2609 (2315,2956) | 73.56 (64.2,83.59) | -0.13 (-0.16 - -0.11) | 4 (2,7) | 0.1 (0.05,0.21) | -4.91 (-5.21 - -4.60) | 300 (211,435) | 18.18 (11.74,28.3) | -3.27 (-3.44 - -3.09) |
|  | Female | 157 (90,262) | 9.16 (4.23,16.73) | 0.02 (-0.11 - 0.15) | 1361 (1201,1532) | 79.75 (69.45,90.46) | -0.04 (-0.05 - -0.03) | 2 (1,4) | 0.1 (0.03,0.25) | -5.40 (-5.79 - -5.02) | 342 (219,552) | 20.1 (11.71,33.79) | -3.56 (-3.80 - -3.32) |
|  | Male | 136 (77,237) | 7.31 (3.29,13.41) | -0.17 (-0.27 - -0.08) | 1249 (1096,1424) | 67.81 (58.66,77.64) | -0.23 (-0.25 - -0.21) | 2 (1,3) | 0.1 (0.04,0.2) | -4.23 (-4.39 - -4.07) | 641 (443,949) | 16.41 (10.53,25.55) | -2.96 (-3.06 - -2.87) |
| Turkmenistan | Both | 167 (102,272) | 8.74 (4.45,15.27) | 0.65 (0.59 - 0.71) | 1237 (1100,1384) | 65.99 (57.57,74.7) | 0.22 (0.20 - 0.24) | 4 (3,5) | 0.2 (0.14,0.28) | -0.15 (-1.76 - 1.49) | 222 (164,287) | 27.19 (19.97,35.39) | -0.10 (-1.18 - 0.99) |
|  | Female | 102 (63,166) | 11.01 (5.74,19.22) | 0.88 (0.82 - 0.94) | 740 (654,827) | 82.1 (71.31,93.13) | 0.52 (0.49 - 0.56) | 2 (1,3) | 0.21 (0.14,0.31) | -0.10 (-1.38 - 1.21) | 278 (203,365) | 31.46 (20.83,44.58) | 0.10 (-0.64 - 0.85) |
|  | Male | 66 (37,108) | 6.63 (3,11.88) | 0.34 (0.27 - 0.41) | 497 (434,565) | 51.07 (43.83,58.88) | -0.14 (-0.15 - -0.12) | 2 (1,3) | 0.2 (0.12,0.28) | -0.34 (-1.80 - 1.15) | 500 (387,622) | 23.26 (16.48,31.03) | -0.40 (-1.38 - 0.59) |
| Tuvalu | Both | 0 (0,1) | 7.69 (3.57,13.8) | -0.32 (-0.37 - -0.26) | 4 (3,4) | 78.41 (68.33,88.42) | -0.64 (-0.66 - -0.62) | 0 (0,0) | 0.2 (0.12,0.31) | -4.84 (-5.08 - -4.59) | 1 (1,1) | 31.17 (21.2,43.52) | -3.66 (-3.86 - -3.46) |
|  | Female | 0 (0,0) | 8.87 (4.17,15.99) | -0.40 (-0.46 - -0.34) | 2 (2,2) | 88.89 (76.91,100.73) | -0.76 (-0.80 - -0.73) | 0 (0,0) | 0.2 (0.1,0.34) | -4.17 (-4.36 - -3.97) | 1 (1,1) | 33.68 (20.57,51.47) | -3.09 (-3.41 - -2.76) |
|  | Male | 0 (0,0) | 6.64 (2.92,12.14) | -0.19 (-0.31 - -0.07) | 2 (2,2) | 69.15 (59.97,79.03) | -0.45 (-0.46 - -0.44) | 0 (0,0) | 0.2 (0.11,0.35) | -5.22 (-5.54 - -4.90) | 2 (1,2) | 29 (17.85,44.28) | -4.15 (-4.42 - -3.89) |
| T眉rkiye | Both | 2043 (1152,3475) | 8.28 (3.77,15.3) | -0.90 (-1.02 - -0.78) | 21010 (18317,23796) | 82.75 (71.45,94.38) | -1.01 (-1.05 - -0.97) | 25 (18,34) | 0.1 (0.06,0.16) | -6.32 (-6.75 - -5.89) | 2219 (1644,2874) | 19.59 (13.92,26.55) | -4.49 (-4.94 - -4.04) |
|  | Female | 1136 (654,1963) | 9.45 (4.4,17.16) | -0.85 (-0.97 - -0.73) | 11288 (9868,12704) | 91.36 (79.07,104.33) | -0.99 (-1.02 - -0.96) | 12 (8,17) | 0.1 (0.06,0.17) | -6.66 (-7.33 - -5.98) | 2664 (1960,3471) | 21.92 (14.37,31.79) | -4.70 (-5.28 - -4.11) |
|  | Male | 907 (502,1576) | 7.18 (3.04,13.39) | -0.95 (-1.09 - -0.81) | 9722 (8401,11042) | 74.59 (63.66,85.85) | -1.03 (-1.08 - -0.98) | 13 (8,19) | 0.1 (0.06,0.17) | -5.97 (-6.43 - -5.50) | 4883 (3726,6120) | 17.39 (11.71,24.76) | -4.39 (-4.77 - -4.00) |
| Uganda | Both | 1799 (1019,3082) | 7.25 (3.24,13.35) | -0.44 (-0.56 - -0.33) | 15959 (13993,17941) | 68.41 (59.15,77.82) | -0.54 (-0.58 - -0.50) | 24 (16,35) | 0.09 (0.06,0.15) | -2.67 (-3.11 - -2.24) | 1705 (1234,2319) | 16.53 (11.06,23.61) | -1.79 (-2.09 - -1.49) |
|  | Female | 986 (556,1664) | 8.02 (3.51,14.63) | -0.39 (-0.54 - -0.24) | 9169 (8028,10399) | 79.7 (68.96,90.89) | -0.49 (-0.52 - -0.45) | 13 (7,22) | 0.11 (0.05,0.2) | -2.74 (-3.22 - -2.25) | 2334 (1603,3224) | 19.52 (11.3,30.53) | -1.76 (-2.09 - -1.43) |
|  | Male | 813 (435,1390) | 6.51 (2.85,12.1) | -0.50 (-0.60 - -0.40) | 6789 (5896,7785) | 57.54 (49.19,66.71) | -0.57 (-0.60 - -0.54) | 11 (6,17) | 0.08 (0.04,0.15) | -2.56 (-3.07 - -2.04) | 4039 (3074,5161) | 13.64 (8.43,21) | -1.79 (-2.09 - -1.48) |
| Ukraine | Both | 608 (332,1034) | 7.08 (3.08,13.28) | -0.41 (-0.52 - -0.29) | 5458 (4379,6746) | 60.95 (48.05,76.13) | -0.30 (-0.33 - -0.26) | 1 (1,1) | 0.01 (0.01,0.02) | -3.80 (-5.32 - -2.25) | 363 (253,499) | 12.19 (7.26,18.13) | -0.73 (-0.99 - -0.48) |
|  | Female | 380 (204,651) | 9.17 (4.03,17.14) | -0.30 (-0.45 - -0.16) | 3639 (2905,4471) | 83.76 (65.42,104.44) | -0.15 (-0.17 - -0.14) | 0 (0,1) | 0.01 (0.01,0.01) | -3.42 (-5.06 - -1.76) | 724 (459,1074) | 16.7 (8.21,27.11) | -0.35 (-0.44 - -0.26) |
|  | Male | 228 (117,405) | 5.11 (1.94,9.83) | -0.61 (-0.87 - -0.36) | 1819 (1452,2263) | 39.46 (30.57,49.98) | -0.53 (-0.57 - -0.50) | 1 (0,1) | 0.02 (0.01,0.02) | -4.03 (-6.23 - -1.77) | 1087 (720,1513) | 7.94 (5.25,11.32) | -1.34 (-1.96 - -0.72) |
| United Arab Emirates | Both | 194 (118,304) | 11.53 (5.72,19.69) | -0.98 (-1.08 - -0.88) | 1844 (1658,2055) | 112.86 (99.8,126.88) | -0.88 (-0.90 - -0.86) | 1 (1,2) | 0.07 (0.04,0.11) | -6.24 (-8.20 - -4.24) | 157 (116,201) | 21.06 (14.28,28.77) | -3.28 (-4.30 - -2.24) |
|  | Female | 109 (67,171) | 13.37 (6.8,22.68) | -0.76 (-0.81 - -0.71) | 997 (896,1112) | 125.69 (110.45,142.51) | -0.68 (-0.70 - -0.66) | 0 (0,1) | 0.06 (0.03,0.1) | -6.49 (-7.94 - -5.02) | 185 (129,248) | 23.28 (13.78,34.78) | -2.84 (-3.51 - -2.16) |
|  | Male | 85 (48,136) | 9.79 (4.5,17.17) | -1.24 (-1.37 - -1.11) | 847 (744,947) | 100.72 (87.53,114.35) | -1.11 (-1.14 - -1.09) | 1 (0,1) | 0.08 (0.04,0.14) | -5.94 (-7.83 - -4.01) | 342 (253,439) | 18.96 (12,27.29) | -3.84 (-4.83 - -2.85) |
| United Kingdom | Both | 682 (307,1200) | 4.37 (1.67,8.46) | -1.43 (-1.63 - -1.23) | 7479 (6074,9042) | 46.8 (37.21,57.62) | -1.22 (-1.24 - -1.20) | 1 (1,1) | 0.01 (0.01,0.01) | -9.03 (-10.03 - -8.02) | 425 (303,563) | 6.81 (4.8,9.1) | -3.62 (-3.94 - -3.28) |
|  | Female | 382 (177,679) | 5.02 (1.97,9.68) | -1.30 (-1.39 - -1.20) | 4337 (3520,5264) | 55.54 (44.21,68.58) | -1.09 (-1.11 - -1.07) | 1 (1,1) | 0.01 (0.01,0.01) | -9.01 (-10.25 - -7.76) | 657 (468,877) | 8.46 (5.92,11.54) | -3.21 (-3.56 - -2.86) |
|  | Male | 300 (135,526) | 3.75 (1.33,7.49) | -1.55 (-1.65 - -1.45) | 3142 (2539,3824) | 38.44 (30.23,47.36) | -1.39 (-1.43 - -1.34) | 1 (1,1) | 0.01 (0.01,0.01) | -9.03 (-9.87 - -8.18) | 1082 (777,1437) | 5.24 (3.69,7.04) | -4.21 (-4.59 - -3.84) |
| United Republic of Tanzania | Both | 2671 (1534,4331) | 8.7 (4.12,15.38) | 0.50 (0.37 - 0.62) | 23361 (20679,26363) | 80.23 (70.25,91.09) | 0.36 (0.32 - 0.40) | 40 (24,62) | 0.13 (0.07,0.22) | -0.73 (-0.99 - -0.47) | 3023 (1822,4630) | 20.73 (13.52,30.23) | -0.32 (-0.41 - -0.23) |
|  | Female | 1434 (827,2347) | 9.25 (4.33,16.58) | 0.42 (0.25 - 0.59) | 13154 (11636,14891) | 89.3 (77.71,101.66) | 0.30 (0.28 - 0.33) | 18 (10,29) | 0.12 (0.06,0.21) | -1.45 (-1.58 - -1.33) | 3270 (2284,4337) | 21.58 (12.72,33.69) | -0.67 (-0.77 - -0.58) |
|  | Male | 1237 (692,2015) | 8.16 (3.83,14.43) | 0.59 (0.56 - 0.63) | 10207 (8918,11536) | 71.04 (61.36,80.93) | 0.45 (0.42 - 0.48) | 22 (8,40) | 0.14 (0.05,0.29) | -0.02 (-0.27 - 0.23) | 6294 (4490,8326) | 19.83 (10.3,33.33) | 0.11 (-0.06 - 0.29) |
| United States of America | Both | 5977 (3251,10143) | 7.44 (3.32,13.63) | -1.03 (-1.15 - -0.90) | 87910 (70243,107372) | 103.85 (81.44,129.27) | -0.79 (-1.02 - -0.56) | 32 (29,35) | 0.04 (0.04,0.05) | -4.30 (-5.53 - -3.06) | 6734 (5069,8667) | 17.64 (12.98,23.05) | -2.18 (-2.73 - -1.62) |
|  | Female | 2937 (1569,5020) | 7.48 (3.32,13.81) | -0.95 (-1.10 - -0.80) | 45022 (35768,55151) | 108.78 (84.98,136.22) | -0.64 (-0.89 - -0.39) | 15 (13,16) | 0.04 (0.04,0.04) | -4.64 (-6.26 - -2.98) | 7894 (5812,10281) | 19.42 (14,25.64) | -1.89 (-2.43 - -1.34) |
|  | Male | 3040 (1698,5158) | 7.4 (3.29,13.53) | -1.10 (-1.25 - -0.95) | 42888 (34474,52520) | 99.13 (77.91,122.99) | -0.86 (-1.02 - -0.70) | 17 (15,19) | 0.04 (0.04,0.05) | -4.31 (-5.55 - -3.06) | 14628 (10928,18692) | 15.94 (11.72,20.69) | -2.29 (-2.64 - -1.95) |
| United States Virgin Islands | Both | 1 (0,2) | 5.64 (2.3,10.76) | 0.06 (-0.02 - 0.14) | 11 (10,13) | 59.64 (51.58,68.21) | 0.02 (0.00 - 0.04) | 0 (0,0) | 0.02 (0.01,0.03) | -4.05 (-6.25 - -1.79) | 1 (0,1) | 6.18 (4.3,8.51) | -1.28 (-2.16 - -0.39) |
|  | Female | 1 (0,1) | 7.16 (2.95,13.35) | 0.20 (0.16 - 0.23) | 7 (6,8) | 74.34 (63.98,85.39) | 0.11 (0.09 - 0.12) | 0 (0,0) | 0 (0,0.01) | -9.66 (-11.36 - -7.92) | 1 (0,1) | 7.09 (4.64,10.21) | -1.56 (-1.78 - -1.33) |
|  | Male | 0 (0,1) | 4.22 (1.41,8.49) | -0.13 (-0.36 - 0.10) | 4 (4,5) | 45.6 (38.93,52.79) | -0.08 (-0.11 - -0.04) | 0 (0,0) | 0.03 (0.01,0.05) | -1.79 (-4.88 - 1.40) | 1 (1,2) | 5.35 (3.53,7.94) | -0.99 (-2.56 - 0.61) |
| Uruguay | Both | 39 (17,73) | 4.26 (1.53,8.49) | -1.19 (-1.28 - -1.10) | 389 (333,440) | 41.02 (34.76,47.48) | -0.84 (-0.90 - -0.79) | 0 (0,0) | 0.03 (0.03,0.04) | -5.51 (-5.79 - -5.23) | 34 (27,42) | 8.29 (6.27,10.71) | -3.31 (-3.82 - -2.79) |
|  | Female | 20 (9,37) | 4.61 (1.69,9.19) | -1.23 (-1.35 - -1.09) | 212 (184,245) | 45.94 (39.08,53.57) | -0.90 (-0.93 - -0.86) | 0 (0,0) | 0.03 (0.02,0.04) | -5.82 (-6.06 - -5.57) | 44 (33,58) | 9.62 (6.89,13.17) | -3.15 (-3.77 - -2.52) |
|  | Male | 18 (8,34) | 3.94 (1.26,8.07) | -1.13 (-1.21 - -1.05) | 176 (150,202) | 36.32 (30.46,42.54) | -0.76 (-0.79 - -0.72) | 0 (0,0) | 0.03 (0.02,0.04) | -5.76 (-7.02 - -4.48) | 78 (61,98) | 7.02 (5.42,8.83) | -3.28 (-4.76 - -1.78) |
| Uzbekistan | Both | 746 (410,1291) | 6.13 (2.6,11.45) | -0.04 (-0.14 - 0.06) | 5793 (5013,6596) | 50.52 (43.14,58.35) | -0.02 (-0.05 - 0.00) | 8 (6,10) | 0.07 (0.05,0.09) | -2.83 (-3.79 - -1.86) | 749 (609,928) | 14.8 (10.84,19.8) | -1.33 (-1.80 - -0.85) |
|  | Female | 456 (251,797) | 7.74 (3.35,14.56) | 0.07 (-0.06 - 0.19) | 3586 (3136,4090) | 64.45 (55.16,74.33) | 0.16 (0.12 - 0.20) | 3 (2,4) | 0.06 (0.04,0.08) | -3.59 (-5.50 - -1.64) | 949 (694,1267) | 16.97 (10.94,24.43) | -1.16 (-1.73 - -0.59) |
|  | Male | 290 (142,515) | 4.62 (1.68,9.04) | -0.17 (-0.20 - -0.13) | 2207 (1863,2538) | 37.38 (31.38,43.87) | -0.17 (-0.19 - -0.16) | 5 (4,6) | 0.08 (0.06,0.11) | -1.99 (-3.61 - -0.34) | 1698 (1321,2151) | 12.77 (9.66,16.29) | -1.32 (-2.18 - -0.44) |
| Vanuatu | Both | 17 (11,26) | 11.46 (6.07,19.11) | -0.01 (-0.04 - 0.02) | 168 (152,184) | 119.95 (106.8,133.48) | -0.24 (-0.25 - -0.23) | 0 (0,1) | 0.24 (0.13,0.39) | -1.19 (-2.18 - -0.19) | 27 (19,38) | 42.66 (28.42,60.22) | -0.78 (-1.39 - -0.17) |
|  | Female | 10 (6,14) | 13.32 (7.07,22.02) | 0.02 (-0.03 - 0.07) | 94 (85,104) | 139.34 (124.37,155.66) | -0.21 (-0.22 - -0.21) | 0 (0,0) | 0.27 (0.13,0.47) | -1.15 (-1.77 - -0.52) | 35 (25,45) | 50.03 (29.16,75.66) | -0.68 (-1.05 - -0.30) |
|  | Male | 8 (5,12) | 9.72 (5.07,16.63) | -0.04 (-0.06 - -0.02) | 74 (66,82) | 101.81 (89.62,114.26) | -0.28 (-0.29 - -0.27) | 0 (0,0) | 0.22 (0.1,0.39) | -1.27 (-2.92 - 0.41) | 62 (48,79) | 35.75 (20.44,56.14) | -0.85 (-1.93 - 0.25) |
| Venezuela (Bolivarian Republic of) | Both | 454 (229,812) | 5.19 (2.11,9.93) | -0.44 (-0.58 - -0.30) | 4367 (3770,4966) | 49.67 (42.31,57.2) | -0.46 (-0.48 - -0.43) | 6 (4,8) | 0.07 (0.05,0.1) | -1.61 (-2.44 - -0.78) | 386 (291,499) | 9.56 (7.18,12.35) | -1.45 (-2.55 - -0.34) |
|  | Female | 272 (150,489) | 6.29 (2.62,11.81) | -0.40 (-0.54 - -0.26) | 2525 (2175,2900) | 57.76 (49.29,66.87) | -0.46 (-0.47 - -0.44) | 3 (2,4) | 0.06 (0.04,0.09) | -1.92 (-3.87 - 0.07) | 449 (340,571) | 10.31 (7.44,13.96) | -1.46 (-2.64 - -0.26) |
|  | Male | 182 (80,330) | 4.11 (1.4,8.34) | -0.53 (-0.67 - -0.38) | 1842 (1589,2097) | 41.63 (35.07,48.41) | -0.46 (-0.48 - -0.45) | 3 (2,4) | 0.07 (0.05,0.11) | -1.36 (-2.25 - -0.46) | 835 (657,1036) | 8.8 (6.4,11.99) | -1.44 (-2.80 - -0.06) |
| Viet Nam | Both | 1897 (1019,3243) | 6.06 (2.58,11.17) | 0.08 (-0.09 - 0.26) | 16457 (14411,18565) | 53.56 (45.93,61.01) | -0.06 (-0.07 - -0.05) | 18 (11,27) | 0.06 (0.03,0.1) | -1.47 (-1.63 - -1.31) | 2329 (1631,3127) | 15.01 (10.04,21.03) | -0.65 (-0.73 - -0.57) |
|  | Female | 1067 (571,1893) | 7.08 (2.94,13.52) | 0.18 (0.02 - 0.34) | 8920 (7718,10174) | 60.38 (51.48,69.27) | 0.08 (0.07 - 0.09) | 5 (3,8) | 0.04 (0.02,0.07) | -2.67 (-2.81 - -2.52) | 2246 (1531,3181) | 15.24 (9.07,24.06) | -0.68 (-0.89 - -0.46) |
|  | Male | 830 (429,1350) | 5.11 (2.05,9.48) | -0.05 (-0.26 - 0.17) | 7537 (6595,8524) | 47.23 (40.65,53.88) | -0.20 (-0.22 - -0.19) | 13 (6,21) | 0.08 (0.04,0.15) | -0.78 (-0.97 - -0.60) | 4575 (3378,6010) | 14.8 (9.48,21.44) | -0.59 (-0.64 - -0.54) |
| Yemen | Both | 1389 (826,2296) | 8.01 (3.92,14.08) | -0.49 (-0.64 - -0.34) | 12228 (10806,13633) | 72.82 (63.64,82.03) | -0.53 (-0.55 - -0.50) | 61 (37,97) | 0.35 (0.17,0.64) | -3.09 (-3.35 - -2.84) | 2803 (1780,4166) | 38.98 (23.27,64.26) | -2.70 (-2.92 - -2.48) |
|  | Female | 749 (438,1209) | 8.88 (4.4,15.68) | -0.45 (-0.61 - -0.29) | 6510 (5693,7327) | 79.51 (68.85,90.74) | -0.50 (-0.52 - -0.48) | 36 (20,66) | 0.42 (0.19,0.88) | -3.45 (-3.94 - -2.96) | 4010 (2577,6322) | 47.06 (25.97,86.99) | -3.00 (-3.48 - -2.50) |
|  | Male | 640 (373,1055) | 7.18 (3.34,12.52) | -0.55 (-0.72 - -0.38) | 5718 (5079,6436) | 66.43 (58.07,75.25) | -0.57 (-0.59 - -0.55) | 25 (13,41) | 0.27 (0.12,0.52) | -2.38 (-2.63 - -2.13) | 6814 (4590,10071) | 31.29 (17.46,52.3) | -2.10 (-2.30 - -1.90) |
| Zambia | Both | 655 (349,1141) | 6.3 (2.7,11.85) | -0.25 (-0.34 - -0.16) | 5867 (5118,6641) | 59.22 (51.03,67.73) | -0.31 (-0.32 - -0.29) | 8 (5,12) | 0.08 (0.04,0.13) | -1.71 (-1.94 - -1.47) | 542 (401,742) | 13.85 (9.36,19.83) | -1.07 (-1.19 - -0.94) |
|  | Female | 370 (206,631) | 7.06 (3.13,13.05) | -0.21 (-0.30 - -0.12) | 3486 (3059,3949) | 69.52 (60.25,79.73) | -0.29 (-0.31 - -0.27) | 5 (3,7) | 0.09 (0.04,0.17) | -1.71 (-1.89 - -1.53) | 840 (590,1148) | 16.74 (10.04,26.13) | -1.05 (-1.17 - -0.93) |
|  | Male | 284 (144,510) | 5.55 (2.23,10.8) | -0.28 (-0.37 - -0.20) | 2381 (2032,2723) | 48.75 (40.93,57.01) | -0.34 (-0.35 - -0.32) | 3 (2,5) | 0.06 (0.03,0.12) | -1.64 (-1.86 - -1.43) | 1382 (1037,1805) | 10.89 (7.27,16.09) | -1.11 (-1.26 - -0.96) |
| Zimbabwe | Both | 583 (331,997) | 7.34 (3.3,13.49) | 0.15 (0.02 - 0.27) | 5088 (4446,5728) | 66.82 (57.52,76.11) | -0.05 (-0.07 - -0.04) | 9 (6,15) | 0.12 (0.06,0.21) | 3.55 (2.64 - 4.47) | 518 (361,733) | 18.05 (11.92,26.97) | 1.26 (0.96 - 1.56) |
|  | Female | 371 (213,619) | 9.3 (4.28,16.67) | 0.01 (-0.08 - 0.10) | 3371 (2942,3785) | 87.85 (75.97,99.89) | -0.14 (-0.16 - -0.12) | 5 (3,10) | 0.13 (0.06,0.27) | 3.61 (2.64 - 4.59) | 864 (601,1273) | 22.39 (13.45,37.39) | 1.05 (0.76 - 1.35) |
|  | Male | 212 (107,380) | 5.39 (2.09,10.49) | 0.39 (0.21 - 0.57) | 1716 (1476,1962) | 45.56 (38.44,52.82) | 0.13 (0.10 - 0.16) | 4 (2,7) | 0.11 (0.05,0.2) | 3.53 (2.82 - 4.24) | 1382 (1008,1879) | 13.68 (8.65,21.16) | 1.66 (1.29 - 2.02) |

**DALYs Disability-Adjusted Life Years, ASR Age-Standardized Rate, UI uncertainty interval, CI confidence interval.**

**Table S9 Percent contribution of risk factors in Ischemic Stroke in 1990 and 2021 by sex and age groups.**

| Factor | Age Groups | Sex | Deaths (95% UI) | | DALYs (95% UI) | |
| --- | --- | --- | --- | --- | --- | --- |
|  |  |  | 1990 | 2021 | 1990 | 2021 |
| High temperature | ＜20 | Both | 1.56 (0.35,3.27) | 2.87 (1.04,5.32) | 1.14 (0.25,2.37) | 1.61 (0.58,3.06) |
|  |  | Female | 1.61 (0.28,3.44) | 2.84 (0.83,5.6) | 1.09 (0.18,2.42) | 1.27 (0.39,2.63) |
|  |  | Male | 1.52 (0.43,3.08) | 2.88 (1.21,5.09) | 1.18 (0.34,2.38) | 1.93 (0.74,3.53) |
|  | ＜5 | Both | 1.72 (0.43,3.52) | 3.1 (1.33,5.45) | 1.65 (0.41,3.35) | 2.81 (1.18,4.9) |
|  |  | Female | 1.74 (0.32,3.69) | 3.01 (0.98,5.76) | 1.65 (0.3,3.45) | 2.52 (0.8,4.91) |
|  |  | Male | 1.7 (0.54,3.32) | 3.14 (1.49,5.3) | 1.65 (0.52,3.19) | 2.97 (1.39,4.98) |
|  | 5-9 | Both | 1.52 (0.24,3.41) | 2.99 (1.1,5.54) | 0.84 (0.12,1.84) | 1.26 (0.45,2.42) |
|  |  | Female | 1.55 (0.11,3.51) | 2.99 (0.63,6.08) | 0.77 (0.06,1.81) | 0.84 (0.17,1.77) |
|  |  | Male | 1.49 (0.34,3.23) | 2.99 (1.3,5.28) | 0.92 (0.21,1.97) | 1.7 (0.69,3.01) |
|  | 10-14 | Both | 1.29 (0.14,2.9) | 2.71 (0.8,5.36) | 0.41 (0.05,0.96) | 0.69 (0.2,1.44) |
|  |  | Female | 1.43 (0.15,3.18) | 2.87 (0.76,5.75) | 0.43 (0.04,1.03) | 0.61 (0.16,1.28) |
|  |  | Male | 1.11 (0.13,2.56) | 2.54 (0.84,4.94) | 0.38 (0.05,0.91) | 0.79 (0.25,1.61) |
|  | 15-19 | Both | 1.04 (0.13,2.32) | 2.44 (0.58,4.92) | 0.48 (0.06,1.09) | 1.03 (0.27,2.18) |
|  |  | Female | 1.2 (0.21,2.56) | 2.58 (0.64,5.17) | 0.52 (0.1,1.15) | 1 (0.26,2.14) |
|  |  | Male | 0.84 (0.04,2.09) | 2.28 (0.54,4.72) | 0.42 (0.02,1.05) | 1.06 (0.25,2.21) |
| Low temperature | ＜20 | Both | 4.42 (3.38,5.57) | 2.75 (1.84,3.86) | 3.14 (2.47,4.07) | 1.5 (1.04,2.11) |
|  |  | Female | 4.59 (3.56,5.8) | 3.18 (2.31,4.2) | 3.08 (2.38,4.05) | 1.4 (0.99,1.96) |
|  |  | Male | 4.25 (3.21,5.44) | 2.48 (1.58,3.67) | 3.21 (2.51,4.13) | 1.6 (1.09,2.23) |
|  | ＜5 | Both | 4.09 (3.08,5.26) | 2.25 (1.37,3.38) | 3.92 (2.99,5.03) | 2.04 (1.26,2.99) |
|  |  | Female | 4.42 (3.4,5.69) | 2.79 (1.88,3.94) | 4.18 (3.24,5.4) | 2.33 (1.58,3.36) |
|  |  | Male | 3.79 (2.78,4.95) | 1.98 (1.16,3.08) | 3.67 (2.7,4.79) | 1.88 (1.12,2.89) |
|  | 5-9 | Both | 4.6 (3.43,5.87) | 2.75 (1.81,3.94) | 2.55 (1.91,3.36) | 1.16 (0.74,1.66) |
|  |  | Female | 4.81 (3.61,6.15) | 3.61 (2.72,4.71) | 2.39 (1.76,3.22) | 1.01 (0.66,1.43) |
|  |  | Male | 4.4 (3.34,5.7) | 2.33 (1.41,3.61) | 2.72 (2.04,3.59) | 1.31 (0.83,1.9) |
|  | 10-14 | Both | 4.94 (3.98,6.03) | 3.17 (2.41,4.2) | 1.56 (1.12,2.18) | 0.8 (0.54,1.12) |
|  |  | Female | 4.82 (3.8,5.95) | 3.34 (2.52,4.35) | 1.44 (1.03,2.09) | 0.71 (0.47,1.03) |
|  |  | Male | 5.1 (4.2,6.22) | 3.01 (2.23,4.11) | 1.73 (1.23,2.34) | 0.94 (0.63,1.33) |
|  | 15-19 | Both | 5.45 (4.52,6.51) | 3.58 (2.86,4.52) | 2.53 (1.96,3.26) | 1.51 (1.14,2.02) |
|  |  | Female | 5 (4.04,6.07) | 3.5 (2.69,4.48) | 2.17 (1.66,2.93) | 1.35 (0.99,1.87) |
|  |  | Male | 6 (5.14,7.11) | 3.69 (2.99,4.71) | 3.01 (2.32,3.76) | 1.72 (1.31,2.27) |
| Alcohol use | ＜20 | Both | -0.14 (-0.36,0.1) | -0.15 (-0.42,0.2) | -0.2 (-0.48,0.12) | -0.2 (-0.51,0.25) |
|  |  | Female | -0.11 (-0.29,0.09) | -0.08 (-0.34,0.18) | -0.16 (-0.42,0.13) | -0.13 (-0.43,0.23) |
|  |  | Male | -0.18 (-0.45,0.11) | -0.21 (-0.54,0.21) | -0.25 (-0.57,0.12) | -0.27 (-0.67,0.25) |

**DALYs Disability-Adjusted Life Years, UI uncertainty interval.**

**Table S10 Projection of the Global Burden of Ischemic Stroke for the Next 15 Years Based on the BAPC Model.**

| **Year** | **Incidence** | | **Prevalence** | | **Death** | | **DALYs** | |
| --- | --- | --- | --- | --- | --- | --- | --- | --- |
|  | **Absolute numbers (95%UI)** | **ASR (per 100,000, 95%UI)** | **Absolute numbers (95%UI)** | **ASR (per 100,000, 95%UI)** | **Absolute numbers (95%UI)** | **ASR (per 100,000, 95%UI)** | **Absolute numbers (95%UI)** | **ASR in (per 100,000, 95%UI)** |
| 2022 | 179098 (174276,183920) | 6.79 (6.61,6.97) | 1595893 (1564713,1627073) | 60.46 (59.28,61.64) | 3448 (3231,3666) | 0.13 (0.12,0.14) | 519621 (492415,546826) | 19.69 (18.66,20.71) |
| 2023 | 183779 (176075,191483) | 6.95 (6.66,7.24) | 1602405 (1557654,1647157) | 60.56 (58.87,62.25) | 3290 (3031,3548) | 0.12 (0.12,0.13) | 504559 (471856,537261) | 19.07 (17.83,20.3) |
| 2024 | 188504 (177035,199974) | 7.11 (6.68,7.54) | 1608341 (1546185,1670497) | 60.67 (58.33,63.01) | 3134 (2826,3443) | 0.12 (0.11,0.13) | 488875 (449339,528411) | 18.44 (16.95,19.93) |
| 2025 | 193266 (177272,209261) | 7.28 (6.68,7.88) | 1613655 (1531137,1696173) | 60.79 (57.68,63.9) | 2983 (2620,3345) | 0.11 (0.1,0.13) | 472505 (425308,519702) | 17.8 (16.02,19.58) |
| 2026 | 197926 (176693,219160) | 7.45 (6.65,8.25) | 1618179 (1512681,1723677) | 60.91 (56.94,64.88) | 2835 (2416,3254) | 0.11 (0.09,0.12) | 455439 (400113,510765) | 17.14 (15.06,19.23) |
| 2027 | 202513 (175333,229692) | 7.62 (6.6,8.64) | 1621920 (1490969,1752871) | 61.04 (56.11,65.97) | 2692 (2217,3167) | 0.1 (0.08,0.12) | 437740 (374094,501387) | 16.47 (14.08,18.87) |
| 2028 | 207079 (173270,240888) | 7.8 (6.52,9.07) | 1624930 (1466501,1783359) | 61.18 (55.22,67.15) | 2552 (2022,3082) | 0.1 (0.08,0.12) | 419582 (347713,491451) | 15.8 (13.09,18.5) |
| 2029 | 211660 (170541,252780) | 7.98 (6.43,9.53) | 1627319 (1439618,1815019) | 61.34 (54.26,68.42) | 2416 (1834,2997) | 0.09 (0.07,0.11) | 401000 (321206,480795) | 15.12 (12.11,18.12) |
| 2030 | 216265 (167146,265384) | 8.17 (6.31,10.02) | 1629278 (1410570,1847986) | 61.52 (53.26,69.78) | 2283 (1653,2913) | 0.09 (0.06,0.11) | 382018 (294728,469308) | 14.42 (11.13,17.72) |
| 2031 | 220779 (162978,278580) | 8.35 (6.17,10.54) | 1630949 (1379423,1882475) | 61.72 (52.2,71.24) | 2154 (1480,2829) | 0.08 (0.06,0.11) | 362693 (268424,456963) | 13.72 (10.16,17.29) |
| 2032 | 225230 (158042,292418) | 8.54 (6,11.09) | 1632638 (1346337,1918939) | 61.94 (51.07,72.8) | 2030 (1315,2744) | 0.08 (0.05,0.1) | 343227 (242528,443926) | 13.02 (9.2,16.84) |
| 2033 | 229640 (152357,306924) | 8.74 (5.8,11.68) | 1634126 (1311270,1956981) | 62.18 (49.89,74.46) | 1910 (1159,2660) | 0.07 (0.04,0.1) | 323991 (217424,430557) | 12.33 (8.27,16.38) |
| 2034 | 234025 (145929,322122) | 8.94 (5.57,12.3) | 1635129 (1274059,1996200) | 62.43 (48.64,76.22) | 1794 (1012,2577) | 0.07 (0.04,0.1) | 305280 (193367,417193) | 11.66 (7.38,15.93) |
| 2035 | 238432 (138775,338090) | 9.14 (5.32,12.96) | 1635663 (1234700,2036625) | 62.69 (47.32,78.06) | 1685 (875,2495) | 0.06 (0.03,0.1) | 287347 (170519,404175) | 11.01 (6.54,15.49) |
| 2036 | 242860 (130866,354855) | 9.35 (5.04,13.66) | 1635804 (1193160,2078449) | 62.95 (45.91,79.99) | 1582 (746,2417) | 0.06 (0.03,0.09) | 270361 (148958,391763) | 10.4 (5.73,15.08) |

**DALYs Disability-Adjusted Life Years, ASR Age-Standardized Rate, UI uncertainty interval.**

**Table S11. Comparing children and adolescents (0–14 years) stroke deaths counts and mortality rates in 1999-2020 from Global Burden of Disease (GBD) and the** **Centers for Disease Control and Prevention Wide-Ranging Online Data for Epidemiological Research (CDC WONDER)**

|  | GBD | | CDC Wonder | |
| --- | --- | --- | --- | --- |
| Year | Absolute number | Crude mortality rate | Absolute number | Crude mortality rate |
| 1999 | 280 | 0.46 (0.45-0.47) | 133 | 0.22 (0.18-0.26) |
| 2000 | 260 | 0.43 (0.42-0.44) | 145 | 0.24 (0.20-0.28) |
| 2001 | 247 | 0.41 (0.40-0.42) | 144 | 0.24 (0.20-0.28) |
| 2002 | 245 | 0.40 (0.39-0.41) | 148 | 0.24 (0.21-0.28) |
| 2003 | 220 | 0.36 (0.35-0.37) | 128 | 0.21 (0.17-0.25) |
| 2004 | 212 | 0.35 (0.34-0.36) | 139 | 0.23 (0.19-0.27) |
| 2005 | 217 | 0.35 (0.34-0.36) | 162 | 0.27 (0.23-0.31) |
| 2006 | 216 | 0.35 (0.34-0.36) | 165 | 0.27 (0.23-0.31) |
| 2007 | 217 | 0.35 (0.34-0.36) | 154 | 0.25 (0.21-0.29) |
| 2008 | 216 | 0.35 (0.34-0.36) | 164 | 0.27 (0.23-0.31) |
| 2009 | 200 | 0.32 (0.32-0.33) | 143 | 0.23 (0.20-0.27) |
| 2010 | 191 | 0.31 (0.30-0.32) | 152 | 0.25 (0.21-0.29) |
| 2011 | 186 | 0.30 (0.29-0.31) | 149 | 0.24 (0.20-0.28) |
| 2012 | 178 | 0.29 (0.28-0.30) | 138 | 0.23 (0.19-0.26) |
| 2013 | 171 | 0.28 (0.27-0.28) | 118 | 0.19 (0.16-0.23) |
| 2014 | 164 | 0.27 (0.26-0.27) | 118 | 0.19 (0.16-0.23) |
| 2015 | 170 | 0.28 (0.27-0.28) | 116 | 0.19 (0.16-0.22) |
| 2016 | 182 | 0.30 (0.29-0.30) | 142 | 0.23 (0.19-0.27) |
| 2017 | 179 | 0.29 (0.29-0.30) | 134 | 0.22 (0.18-0.26) |
| 2018 | 166 | 0.27 (0.27-0.28) | 119 | 0.20 (0.16-0.23) |
| 2019 | 164 | 0.27 (0.26-0.28) | 113 | 0.19 (0.15-0.22) |
| 2020 | 152 | 0.25 (0.24-0.27) | 100 | 0.17 (0.13-0.20) |

Data source & case definition

CDC WONDER Underlying Cause of Death: ICD-10 I60-64 and I69.0-69.4 as underlying cause; population 0–14 years; 1999–2020.

GBD Cause of death or injury: United States of America; stroke; 0-14 years; 1999-2020.
