# Supplementary material for: Epidemiological trends and future projections of ischemic stroke in children and adolescents: a global analysis from 1990 to 2021
Source: Front Neurol. 2025 Oct 22;16:1662610. doi: 10.3389/fneur.2025.1662610 (PMC12586142; doi:10.3389/fneur.2025.1662610)
Supplement: Supplementary file 2 [file Table_2.docx]

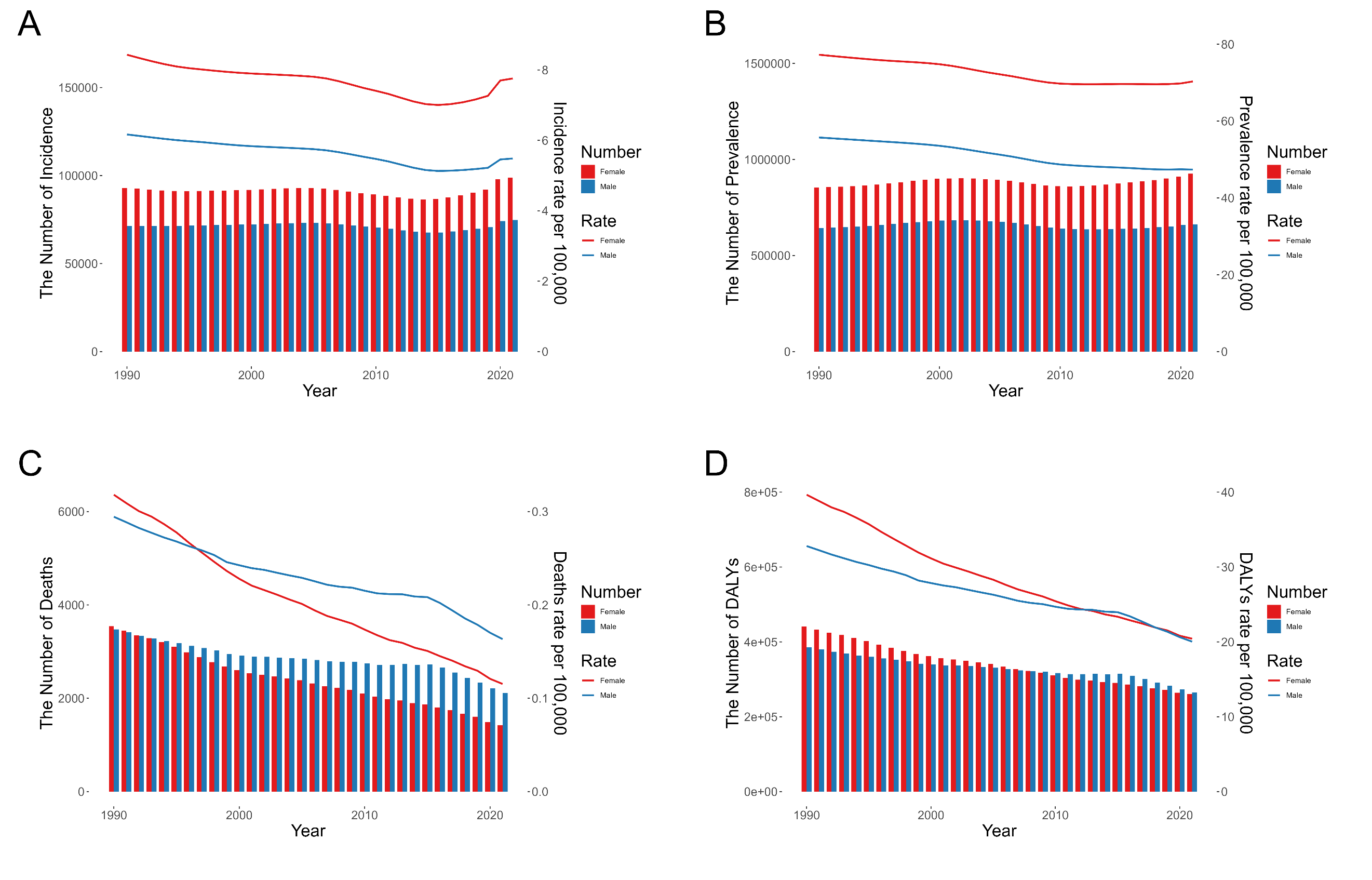


**Fig. S1** The Number and Age-Standardized Rate of the Global Burden of Ischemic Stroke. (A) Incidence. (B) Prevalence. (C) Deaths. (D) DALYs. DALYs: Disability-Adjusted Life Years.


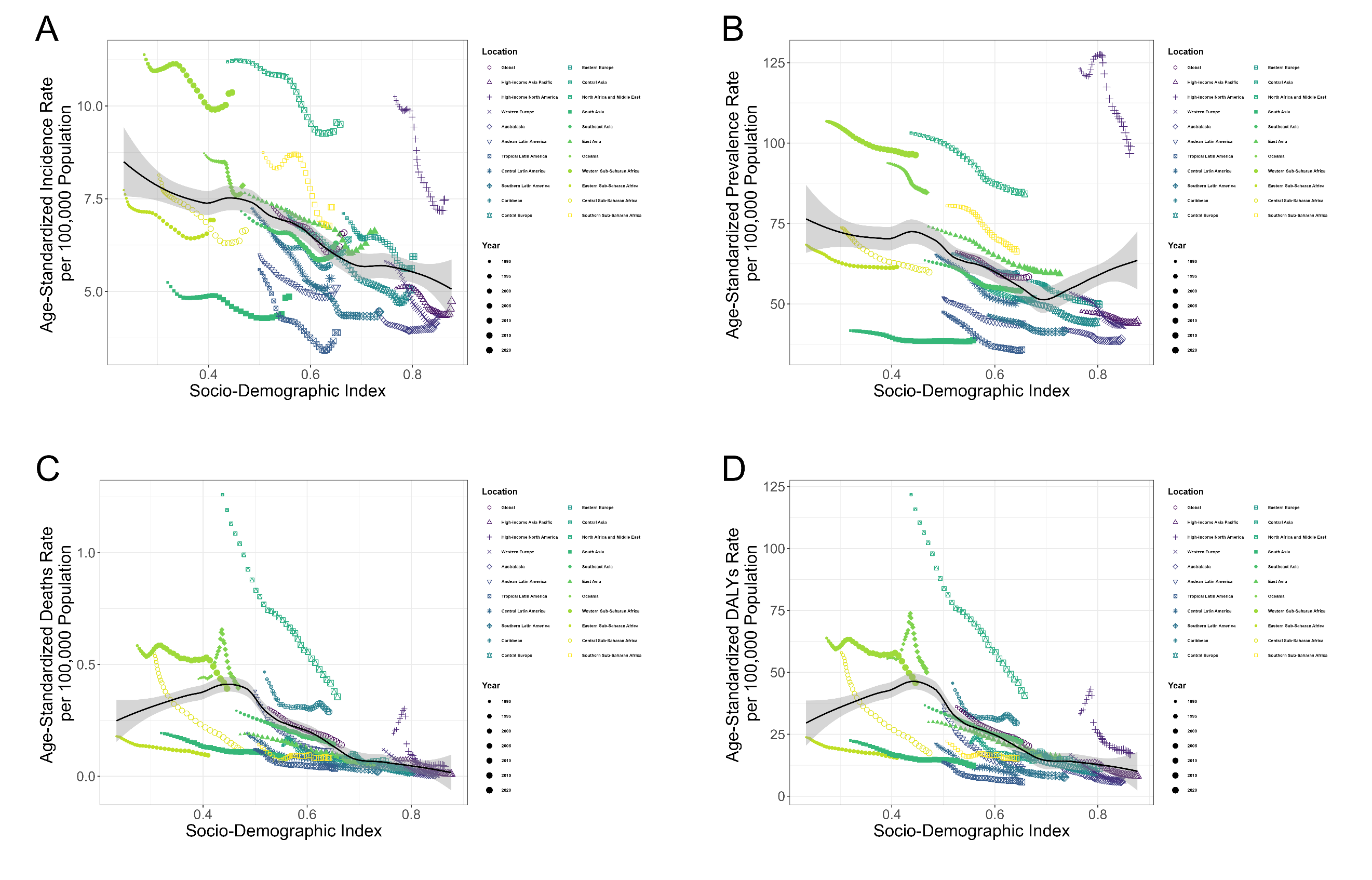


**Fig. S2** Burden of Ischemic Stroke for Children and Adolescents Across 21 Regions by SDI. (A) ASIR for 21 regions by SDI from 1990 to 2021. (B) ASPR for 21 regions by SDI from 1990 to 2021. (C) ASMR for 21 regions by SDI from 1990 to 2021. (D) ASDR for 21 regions by SDI from 1990 to 2021. ASIR Age-Standardized Incidence Rate, ASPR Age-Standardized Prevalence Rate, ASMR Age-Standardized Mortality Rate, ASDR Age-Standardized Disability-Adjusted Life Year Rate, SDI Socio-Demographic Index.


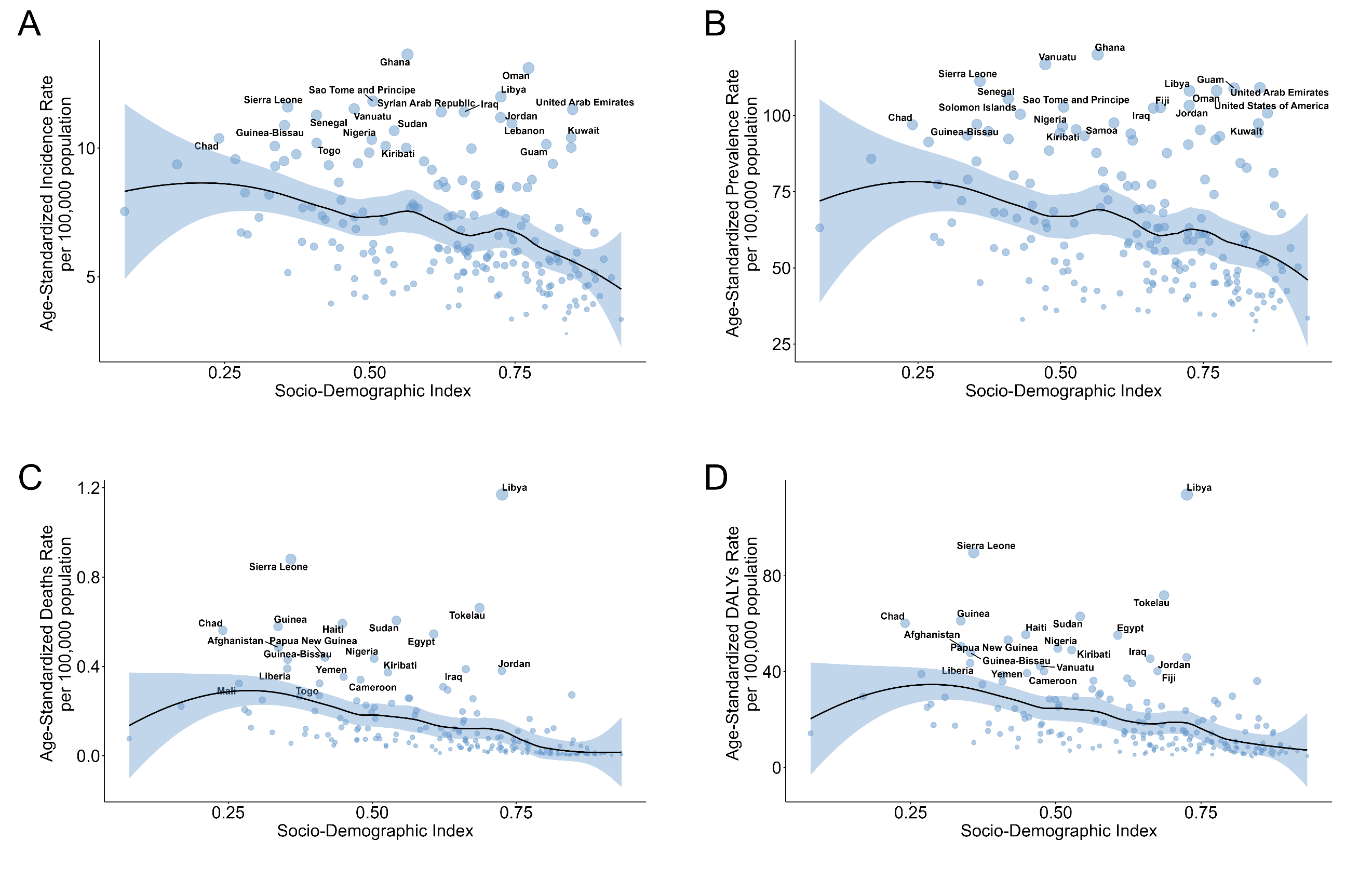


**Fig. S3** Burden of Ischemic Stroke for Children and Adolescents Across 204 countries by SDI. (A) ASIR for 204 countries by SDI from 1990 to 2021. (B) ASPR for 204 countries by SDI from 1990 to 2021. (C) ASMR for 204 countries by SDI from 1990 to 2021. (D) ASDR for 204 countries by SDI from 1990 to 2021. The chart highlights the 15 countries with the heaviest disease burden. ASIR Age-Standardized Incidence Rate, ASPR Age-Standardized Prevalence Rate, ASMR Age-Standardized Mortality Rate, ASDR Age-Standardized Disability-Adjusted Life Year Rate, SDI Socio-Demographic Index.


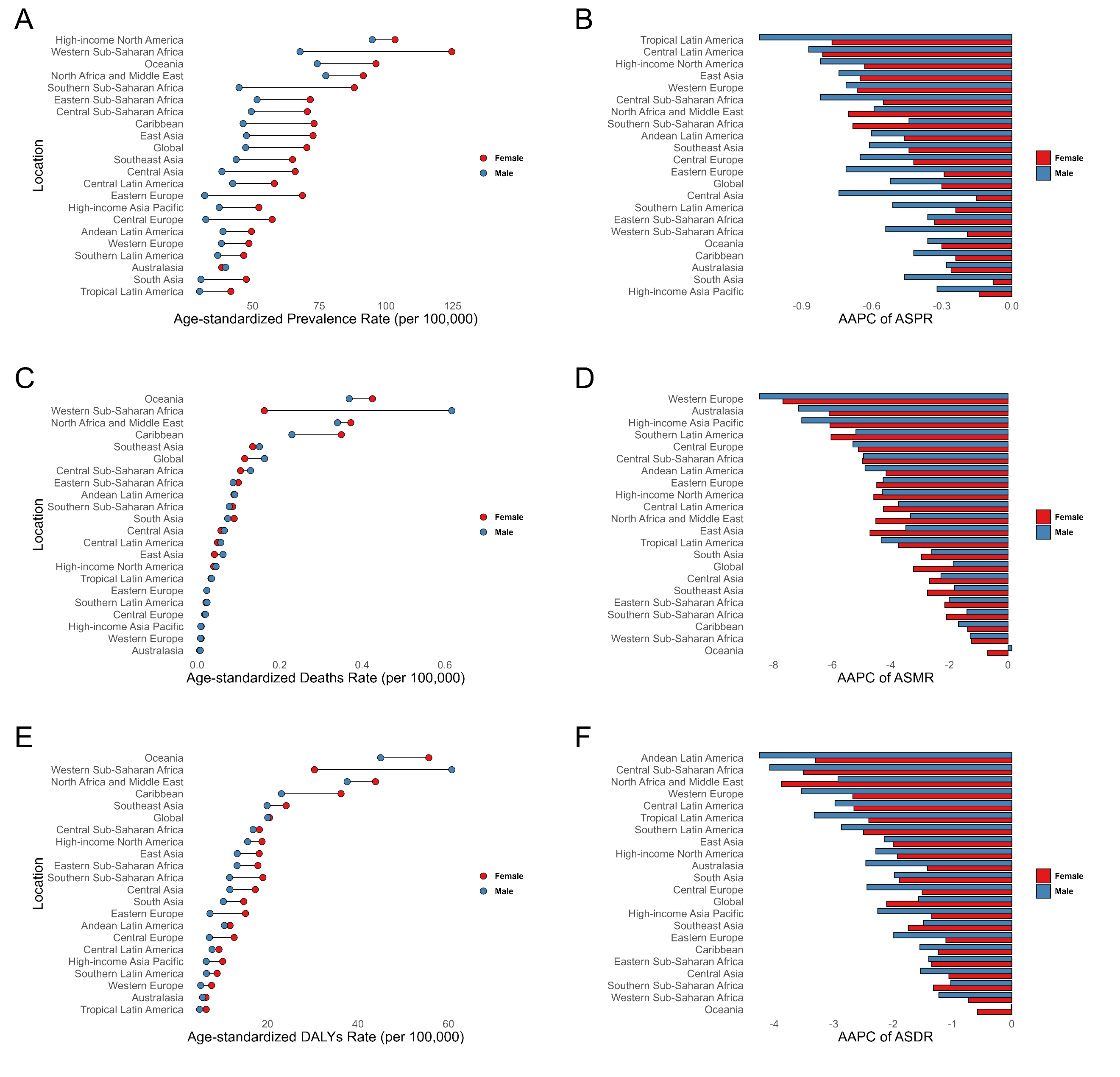


**Fig. S4** Age-Standardized Rates of Prevalence, Deaths, and DALYs, Along with Their Average Annual Percent Change from 1990 to 2021, in Ischemic Stroke Among Children and Adolescents Across 21 Regions. (A) and (B) Prevalence. (C) and (D) Deaths. (E) and (F) DALYs. DALYs: Disability-Adjusted Life Years.


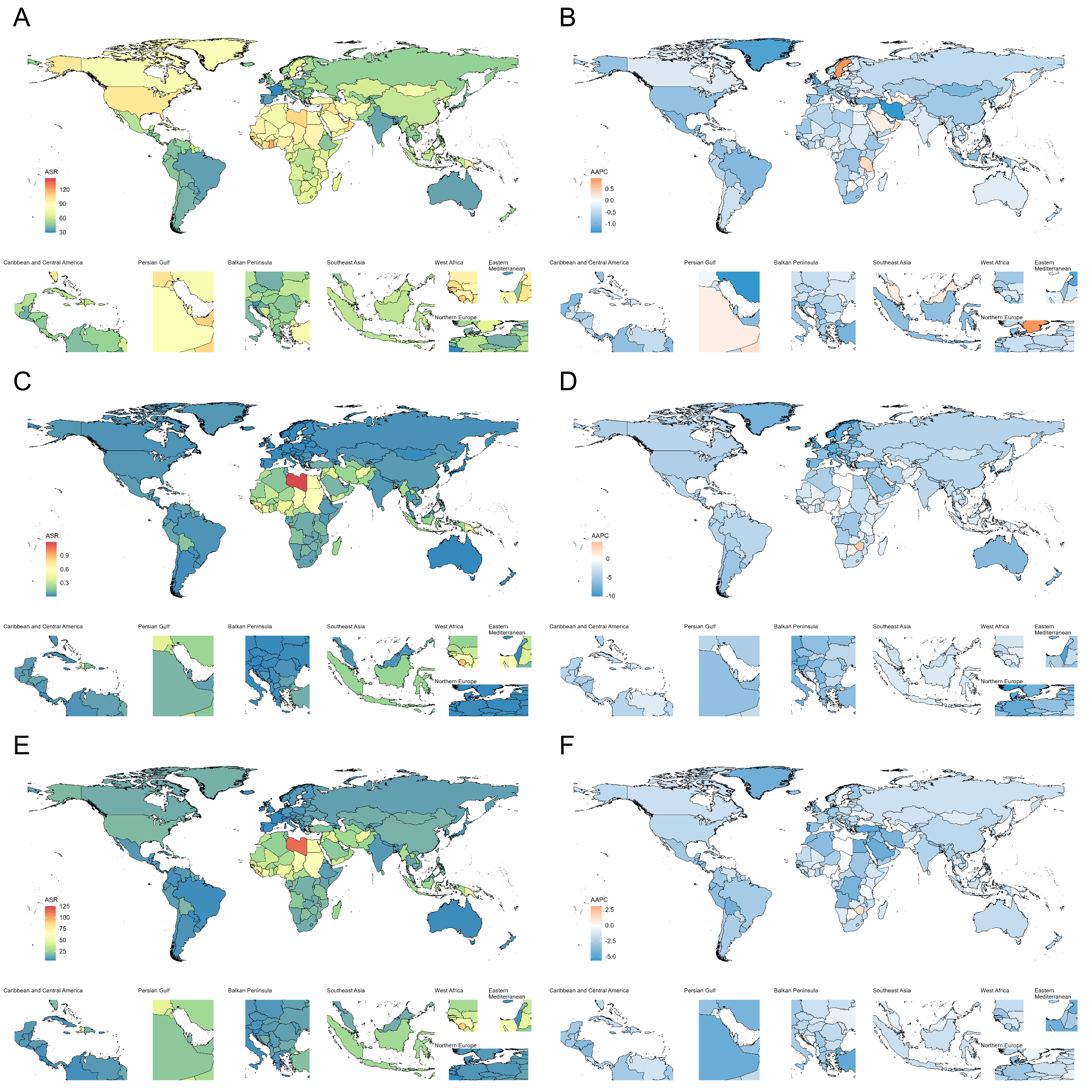


**Fig. S5** Age-Standardized Rates of Prevalence, Deaths, and DALYs, Along with Their Average Annual Percent Change from 1990 to 2021, in Ischemic Stroke Among Children and Adolescents Across countries. (A) and (B) Prevalence. (C) and (D) Deaths. (E) and (F) DALYs. DALYs: Disability-Adjusted Life Years.


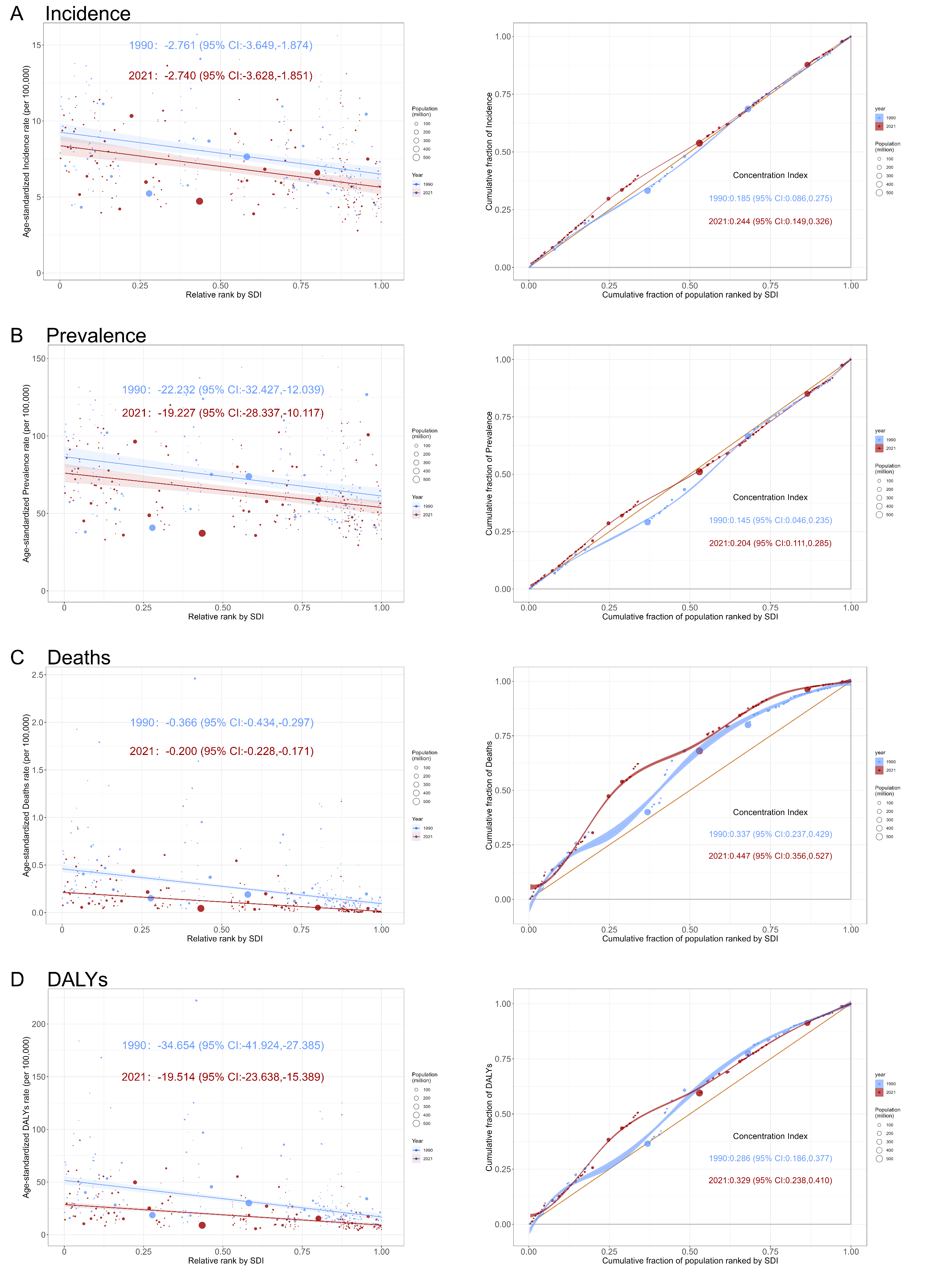


**Fig. S6** Slope Index of Inequality (Left) and Concentration Index (Right) Curves for Ischemic Stroke Among Children and Adolescents in 1990 and 2021. (A) Incidence. (B) Prevalence. (C) Deaths. (D) DALYs. DALYs: Disability-Adjusted Life Years.


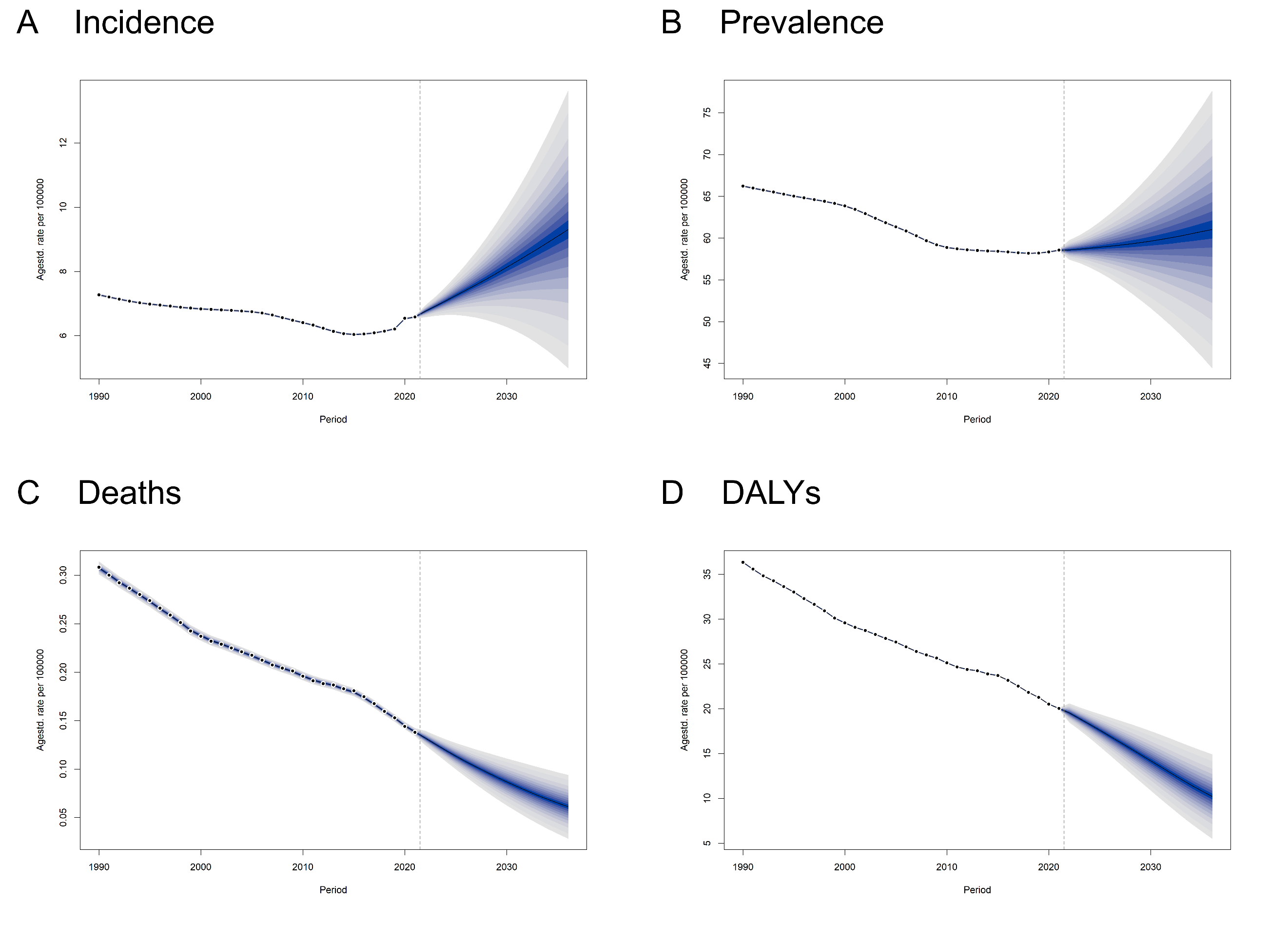


**Fig. S7** Trends and Forecasts of the Burden of Ischemic Stroke for Children and Adolescents Globally Until 2036. (A) Incidence. (B) Prevalence. (C) Deaths. (D) DALYs (Disability-Adjusted Life Years).


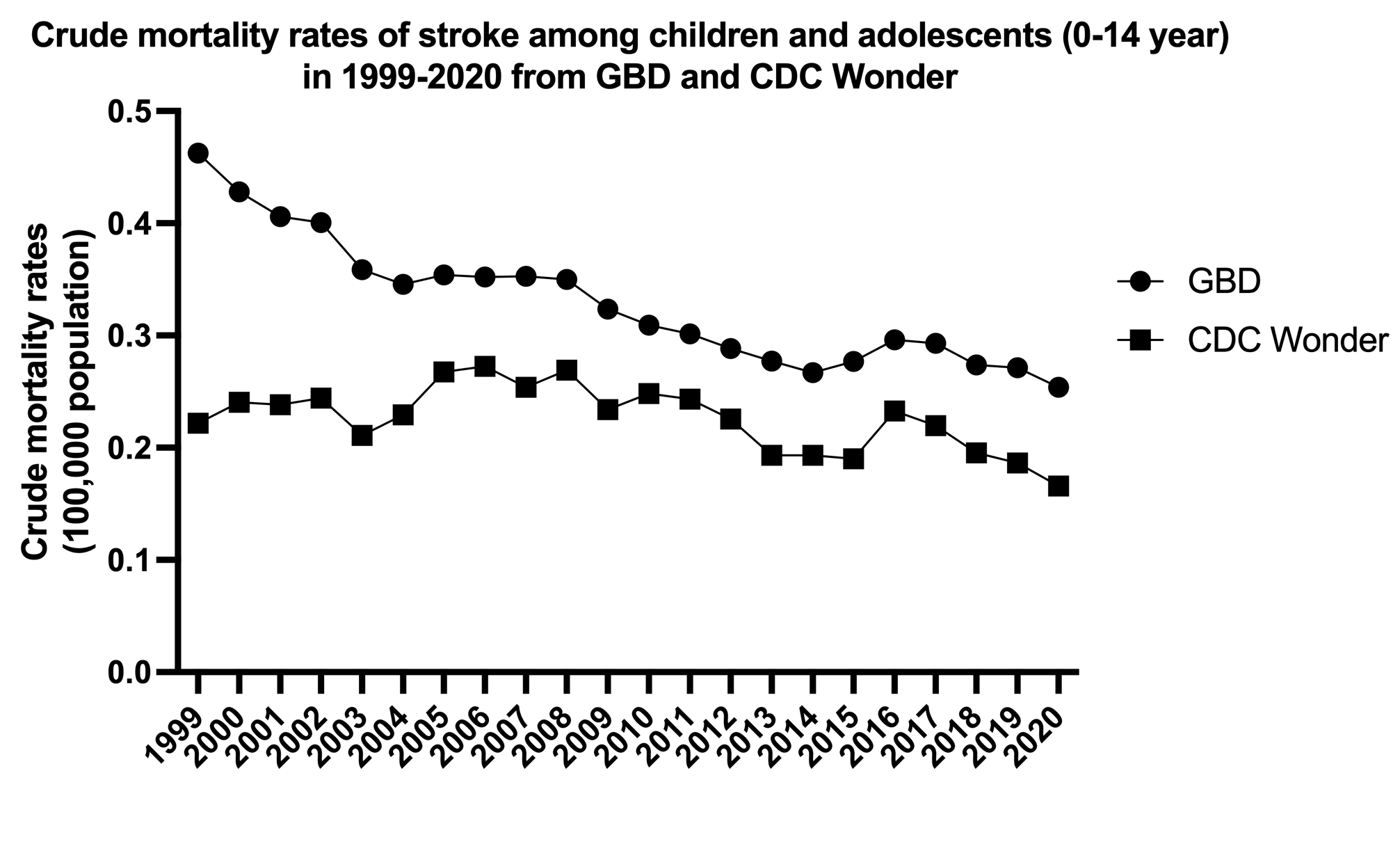


**Fig. S8** Crude mortality rates of stroke among children and adolescents (0-14 year) in 1999-2020 from GBD and CDC Wonder. GBD: Global Burden of Disease; CDC Wonder: the Centers for Disease Control and Prevention Wide-Ranging Online Data for Epidemiological Research.
